# The Optimising Cardiac Surgery ouTcOmes in People with diabeteS (OCTOPuS) randomised controlled trial to evaluate an outpatient pre-cardiac surgery diabetes management intervention: a study protocol

**DOI:** 10.1136/bmjopen-2021-050919

**Published:** 2021-06-09

**Authors:** Richard Ian Gregory Holt, Giorgos Dritsakis, Katharine Barnard-Kelly, Kerensa Thorne, Amy Whitehead, Lauren Cohen, Elizabeth Dixon, Mayank Patel, Philip Newland-Jones, Mark Green, Helen Partridge, Suvitesh Luthra, Sunil Ohri, Kareem Salhiyyah, Joanne Lord, John Niven, Andrew Cook, OCTOPuS study group

**Affiliations:** 1 Human Development and Health, Faculty of Medicine, University of Southampton, Southampton, UK; 2 Southampton National Institute for Health Research Biomedical Research Centre, University Hospital Southampton NHS Foundation Trust, Southampton, UK; 3 Clinical Trial Units, University of Southampton, Southampton, UK; 4 Barnard Health – Health Psychology Research, Fareham, UK; 5 Department of Diabetes and Endocrinology, University Hospital Southampton, Southampton, Hampshire, UK; 6 Diabetes and Endocrinology, Royal Bournemouth and Christchurch Hospitals NHS Foundation Trust, Bournemouth, Bournemouth, UK; 7 Division of Cardiac Surgery, Wessex Cardiothoracic Centre, University Hospital Southampton, Southampton, Hampshire, UK; 8 Middle East University, Amman, Jordan; 9 Southampton Health Technology Assessments Centre, University of Southampton, Southampton, Hampshire, UK; 10 Patient Representative, Southampton, UK; 11 Wessex Institute, University of Southampton, Southampton, Hampshire, UK

**Keywords:** cardiothoracic surgery, diabetes & endocrinology, surgery

## Abstract

**Introduction:**

Cardiothoracic surgical outcomes are poorer in people with diabetes compared with those without diabetes. There are two important uncertainties in the management of people with diabetes undergoing major surgery: (1) how to improve diabetes management in the weeks leading up to an elective procedure and (2) whether that improved management leads to better postoperative outcomes. We previously demonstrated the feasibility of delivering the Optimising Cardiac Surgery ouTcOmes in People with diabeteS (OCTOPuS) intervention, an outpatient intervention delivered by diabetes healthcare professionals for people with suboptimally managed diabetes over 8–12 weeks before elective cardiac surgery. The present study will assess the clinical and cost-effectiveness of the intervention in cardiothoracic centres across the UK.

**Methods and analysis:**

A multicentre, parallel group, single-blinded 1:1 individually randomised trial comparing time from surgery until clinically fit for discharge in adults with suboptimally managed type 1 diabetes or type 2 diabetes undergoing elective surgery between the OCTOPuS intervention and usual care (primary endpoint). Secondary endpoints will include actual time from surgery to discharge from hospital; days alive and either out of hospital or judged as clinically fit for discharge; mortality; time on intensive therapy unit (ITU)/ventilator; infections; acute myocardial infarction; change in weight; effect on postoperative renal function and incidence of acute kidney injury; change in HbA_1c_; frequency and severity of self-reported hypoglycaemia; operations permanently cancelled for suboptimal glycaemic levels; cost-effectiveness; psychosocial questionnaires. The target sample size will be 426 recruited across approximately 15 sites. The primary analysis will be conducted on an intention-to-treat population. A two-sided p value of 0.05 or less will be used to declare statistical significance for all analyses and results will be presented with 95% CIs.

**Ethics and dissemination:**

The trial was approved by the South Central–Hampshire A Research Ethics Committee (20/SC/0271). Results will be disseminated through conferences, scientific journals, newsletters, magazines and social media.

**Trial registration number:**

ISRCTN10170306.

**Strengths and limitations of this study**

- The Optimising Cardiac Surgery ouTcOmes in People with diabeteS (OCTOPuS) intervention was developed according to the Medical Research Council (MRC) framework for complex interventions and successfully piloted in a single cardiothoracic surgical centre.
- This is the first trial to assess whether early contact with a specialist diabetes team in the weeks leading up to surgery improves cardiothoracic surgical outcomes and reduces the excess morbidity and mortality experienced by people with diabetes.
- Hospital length of stay is an important clinical and economic measure of the success of surgery.
- The sample size and number of sites will mean that the results are sufficiently generalisable to the remaining cardiothoracic centres across the UK.
- The start of the study will likely be delayed by COVID-19 because of the effect of the pandemic on elective surgery.

## Introduction

The prevalence of cardiovascular disease is increased approximately twofold in people with diabetes after adjustment for other cardiovascular risk factors.^1^ It affects approximately a third of all people with type 2 diabetes and contributes to over 50% of deaths.^2^ As coronary heart disease in people with diabetes tends to be more diffuse affecting multiple vessels, coronary artery bypass grafting is often the preferred method for revascularisation. Approximately 30%–40% of all people undergoing open cardiac surgery have diabetes.^3^

Surgical outcomes are worse in people with diabetes, with an up to threefold higher risk of postoperative complications which include poor healing, wound complications and renal dysfunction.^4 5^ These complications are associated with longer hospital stay and higher readmission rates. The reasons underlying the poorer outcomes include hyperglycaemia, dyslipidaemia and obesity. Although national and international groups have published detailed guidelines to improve surgical outcomes in people with diabetes, many people with diabetes are poorly prepared for surgery.^6–8^ In the European Multicenter Study on Coronary Artery Bypass Grafting (E-CABG) study, 54% of people with type 2 diabetes treated with non-insulin medications and 67% of those with insulin-treated diabetes had an HbA_1c_ above 53 mmol/mol (7.0%) prior to cardiac surgery.^5^

There are two important uncertainties in the management of people with suboptimally managed diabetes undergoing major surgery: (1) how to improve diabetes management in the weeks leading to elective surgery and (2) whether that improved management is reflected in better surgical outcomes. To address these gaps, the overarching aim of the Optimising Cardiac Surgery ouTcOmes in People with diabeteS (OCTOPuS) project is to develop and test whether a preoperative outpatient intervention to improve diabetes management improves cardiac surgical outcomes.

The development of the intervention is described in detail elsewhere (Holt *et al*,^9^ Under review). In summary, the prototype OCTOPuS intervention was based on a nurse-led outpatient intervention that has been used in Royal Bournemouth Hospital for 7 years and incorporated the findings of two rapid literature reviews. A feasibility study conducted in 17 people with diabetes undergoing cardiothoracic surgery at the University Hospital Southampton showed that it is possible to develop a clinical pathway to deliver the OCTOPuS intervention to improve glycaemic management prior to admission that was acceptable for people with diabetes and clinicians.

The present study will be a multicentre randomised controlled trial (RCT) in cardiothoracic centres across the UK to assess the clinical and cost-effectiveness of the intervention.

## Methods and analysis

### Study design

OCTOPuS is a multicentre, parallel group, single blind, individually randomised controlled trial incorporating a preplanned futility analysis. It will compare time from surgery until an individual is clinically fit for discharge in adults with suboptimally managed type 1 diabetes or type 2 diabetes undergoing elective cardiothoracic surgery between the OCTOPuS intervention and usual care. The provisional planned trial recruitment dates are 1 September 2021–31 August 2023. These are contingent on the reopening of elective cardiothoracic surgery and research capacity following the latest national COVID-19 lockdown.

### Eligibility

#### Inclusion criteria

1. Aged ≥18 years old with type 1 diabetes or type 2 diabetes.
2. Suboptimally managed diabetes defined as an HbA_1c_ >53 mmol/mol (7%) for those ≤75 years old and an HbA_1c_ >64 mmol/mol (8%) for those >75 years old. The higher HbA_1c_ criterion for older people is to minimise the risk of iatrogenic hypoglycaemia.^10^ HbA_1c_will be measured using a near patient test at the cardiothoracic surgery outpatient appointment where the decision to proceed to surgery is made.
3. Awaiting elective open-heart cardiac surgery.
4. Anticipated delay before surgery of at least 2 months.
5. Surgery will take place at a hospital participating in the trial.
6. Ability to give informed consent.
7. Ability to interact with the study documentation and processes.

#### Exclusion criteria

1. Active malignancy, where the malignancy is currently being treated by chemotherapy, surgery or radiotherapy or is likely to cause death within 6 months.
2. Pregnancy.
3. Previous cardiac surgery.
4. Known haemoglobinopathies that affect the measurement of HbA_1c_.
5. Other illnesses or conditions that would preclude engagement with the OCTOPuS intervention.
6. Surgery taking place outside the participating hospitals, for example, at a private hospital.

### Recruitment

#### Screening and consent

Outpatient cardiac surgery appointment clinic lists will be scrutinised ahead of appointments and an information sheet explaining the trial will be sent by post or email as appropriate to people who appear eligible (including contact details to opt out if the person does not want further contact about the trial). Before the outpatient appointment, a researcher will contact the prospective participant to discuss the study at least 24 hours before the appointment allowing time for reflection and discussion. This will permit eligible individuals to be randomised immediately after the outpatient appointment, and where possible receive their first OCTOPuS consultation, on the same day. The treating surgeon will remind eligible patients about the trial if a decision to proceed to surgery is made. If the person wishes to participate, he or she will have the opportunity to discuss the study face-to-face with a research nurse before they give written consent. Final eligibility criteria will be checked prior to recruitment. Patients whose medical records cannot be accessed prior to the appointment to determine eligibility (eg, patients from another hospital) will be given information about the study on the day of the appointment and will be offered the opportunity to attend another day to discuss participation.

#### Randomisation

**Figure 1:**
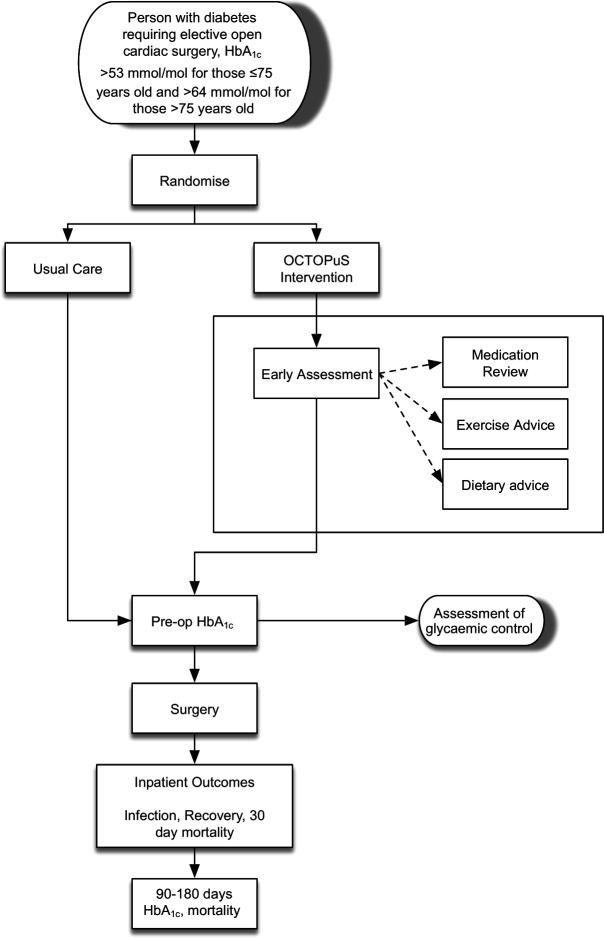
OCTOPuS (Optimising Cardiac Surgery ouTcOmes in People with diabeteS) study flowchart.

Participants will be individually randomised in a 1:1 ratio, stratified by centre and age (≤75 and >75 years old), using permuted blocks. The study flow is illustrated in figure 1.

### Study procedures

#### Baseline measurements

After randomisation, the following data will be collected on participants in both arms: medical history and examination; vital signs; biochemistry; self-reported episodes of hypoglycaemia.

#### The OCTOPuS intervention

##### Initial consultation

Participants randomised to receive the OCTOPuS intervention will have an initial consultation with an OCTOPuS practitioner, who may be a doctor, nurse, pharmacist or other appropriately trained healthcare professional. In this consultation, the participant’s diabetes management will be discussed, as well as the likely benefits of improved glycaemic management prior to surgery. The practitioner and participant will agree actions, tailored to the individual needs and ability, including the following:

- A graded exercise regimen. This may be completely self-delivered, or alternatively by joining a local appropriate exercise scheme, such as a ‘health walk’.
- Dietary advice, supplemented by a consultation with a dietitian if needed.
- Medication review, which may lead to the introduction of insulin or other diabetes medications for people with type 2 diabetes.
- Specific advice about managing expectations, understanding facilitators to achieve change and overcoming barriers to improve medical and psychosocial outcomes.

The exact process and the treatment options are set out in the OCTOPuS intervention manual (online supplemental material 1).

10.1136/bmjopen-2021-050919.supp1 Supplementary data

##### Support calls

After the initial consultation, participants will receive regular review with an OCTOPuS practitioner, usually by telephone, at least once a fortnight until the participant’s diabetes management goals have been reached and no further changes are needed. After this, the frequency of the calls can be reduced at the discretion of the OCTOPuS practitioner and participant to a minimum of every 6 weeks. This contact will be an opportunity to offer encouragement and support and address any issues which have arisen for the participant. One more support contact will be made 1–3 weeks after discharge to ensure the continuity of diabetes management beyond surgery. Where necessary, the OCTOPuS practitioner will liaise with local services, for example, the participant’s GP or a dietitian, to facilitate delivery.

#### Control arm

Participants in the control arm will receive usual care in the cardiac surgery centre attended by the individual. This is likely to contain standardised brief advice from the surgeon to pay attention to their diabetes prior to surgery. Some people may act on this advice, either on their own or in conjunction with their GP. The study will document ‘usual care’ at all recruiting centres and explore with participants in the control arm as part of the qualitative work what actions were taken in response to advice received.

#### Follow-Up visits

After participants are randomised to either the intervention or control arm, data will be collected from them at the following timepoints: presurgery; discharge; 7 days postsurgery; 30 days postsurgery; and at their next routine diabetes care visit between 90 and 180 days postsurgery. In addition to the baseline measures, information about the surgery, infections and surgical complications, mortality and adverse events will be collected (table 1). Presurgery, surgery and discharge data will be collected in hospital. After discharge, data will be collected remotely, for example, over the phone, by post, through inpatient note review or where possible using adult cardiac surgery databases (eg, the SCTS National Adult Cardiac Surgery Audit).

**Table 1:** Summary of data collection during the OCTOPuS study at various timepoints

Timepoint	Screening	Consent	Baseline	Intervention	Support calls (every 2–6 weeks post-op)	Presurgery assessments	Surgery	Discharge	Surgery +7 days	Post-discharge support call	Surgery +30 days	Surgery +90 days	End of study
Notes review	X												
Informed consent		X											
Eligibility evaluation (incl. pregnancy test where appropriate)	X	X	X										
Medical history (incl. smoking status, diabetes and current medications)			X			X							
Physical examination (incl. height, weight and waist circumference)			X (height will only be recorded at baseline)			X							
Vital signs (incl. BP)			X			X							
Biochemistry (incl. HbA_1c_, blood glucose and renal function)			X			X		X (only serum creatinine and renal function to capture acute kidney failure)				X (between 90–180 days post-op)	
Hypoglycaemia			X		X	X							
Infections and surgical complications								x			x	X	
Mortality						x					x	x	x
Intervention				X									
Intervention support phone call (incl. review of diary card and components of intervention used)					X					x			
Practitioner time (cost-effectiveness)			X	X	X					X			
NHS resource use questions (cost-effectiveness)						X							
Surgery (incl. time on ventilator/ITU)							X						
Blinded assessment								X					
Adverse events			X	X	X	X			X	X	X	X	X
EQ-5D-5L			X						X		X	X	
Participant qualitative interview			X									X	
Psychosocial questionnaires			X									X	

BP, blood pressure; ITU, Intensive therapy unit; NHS, National Health Service.

### Endpoints

#### Primary endpoint

Time from surgery until clinically fit for discharge, as judged by the surgical team. Teams will be blinded to pre-hospital diabetes management allocation. This primary outcome was chosen because reduced time in hospital (though not at the expense of safety) is valued by people with diabetes, clinicians and commissioners.

#### Secondary endpoints

- Time from surgery to actual discharge from hospital—this recognises that discharge can be delayed for non-clinical reasons.
- Days alive between surgery and either out of hospital or judged as clinically fit for discharge.
- Preoperative mortality; 30-day mortality; 90-day mortality.
- Time on ITU.
- Time on a ventilator.
- Sternal wound infections, defined according to the National Institute for Health and Care Excellence (NICE) guidance and the Centers for Disease Control and Prevention (CDC) criteria.^11 12^
- Leg wound infections, in those who provide donor veins; graded according to the CDC definitions of surgical site infections.^13^
- Chest infections, defined as a change in typical chest symptoms (cough, increase respiratory rate, shortness of breath) in conjunction with a fever or inflammatory markers.
- Urinary tract infections, defined as ‘clinically diagnosed and treated, whether or not results from a urine culture are available’.
- Acute coronary syndrome.^13^
- Change in weight between randomisation and surgery.
- Effect on postoperative renal function and incidence of acute kidney injury as assessed by measurement of serum creatinine and calculation of estimated glomerular filtration rates.^13^
- HbA_1c_ immediately preoperative, and at between 90 and 180 days post operation.
- Change in HbA_1c_ between baseline and immediately preoperative, and change from preoperative to between 90 and 180 days post operation.
- Operations cancelled for suboptimal glycaemic management.
- Frequency and severity of self-reported overall, minor, severe and nocturnal hypoglycaemia assessed at baseline, during the Support Contact and Pre-surgery.^14^
- EQ-5D at baseline, 7, 30 and 90 days postsurgery.
- Qualitative interviews and psychosocial questionnaires at baseline and 90 days postsurgery to explore participants’ experiences and perceived benefits of the intervention and any changes to their diabetes self-management.
- Cost-effectiveness of intervention, including use of NHS lifestyle improvement programmes and diabetes services; use of medication, time spent by practitioners for training, delivering the intervention and liaising with local services; HbA_1c_ point-of-care and blood glucose monitoring costs.

### Sample size

#### Futility assessment: physiological effect of intervention

To demonstrate that a physiological response is plausible, we need to show an HbA_1c_ reduction of 5 mmol/mol in the intervention group presurgery compared with baseline. Previous experience shows the mean initial HbA_1c_ in our study population is approximately 72 mmol/mol, with a SD of 15 mmol/mol.^15 16^ For an expected change in HbA_1c_ from baseline of 5 mmol/mol in the intervention group, and assuming a correlation of 50% between baseline and presurgery, a sample size in the intervention group of 50 participants would allow a margin of error of 4.16 below the mean for a 95% CI and would, therefore, allow us to exclude a difference of zero if the treatment difference of 5 was observed.

#### Intervention effectiveness: clinical outcomes

The primary outcome is the time from surgery to when the responsible consultant considers the participant clinically fit for discharge. We will not consider the actual discharge date in the primary analysis, as currently many elective cardiothoracic surgical patients remain in hospital longer than clinically indicated due to their social situation. Discussions with clinicians and commissioners suggest that a mean improvement of half a day would be clinically worthwhile.

The current mean duration postsurgery until clinically fit for discharge is 7 days, with an SD of 1.5 days. To demonstrate an improvement of 0.5 days with 90% power and 5% significance with 1:1 randomisation between intervention and control arms would require a total of 382 participants (nQuery V.7.0). We will allow for a 5% loss to follow-up, and 5% for deaths post randomisation inflating the final target sample size to 426 participants. Participants will be recruited across approximately 15 UK cardiothoracic centres.

### Interim analysis

Futility will be assessed, and the trial could be stopped early for one of two main reasons:

#### Recruitment and delivery

There are several threats to recruitment and delivery of this trial:

- Being unable to recruit and initiate sufficient centres.
- Centres being unable to recruit sufficient participants.
- Centres being unable to deliver the OCTOPuS intervention.

Therefore throughout the trial, we will review progress against criteria at three timepoints, grading trial progress as red, amber or green each time (online supplemental material 2).

10.1136/bmjopen-2021-050919.supp2 Supplementary data

#### Physiological effect of intervention

It is believed that the OCTOPuS intervention will have its clinically relevant effects through improvement of clinical measures, including change in body weight, exercise, lipid profile and blood pressure. However, the main target of the intervention is to improve glycaemic management; if no physiological effect can be demonstrated on glycaemic measures, continuation of the trial would be considered futile. After the first 100 participants have had their surgery, we will assess the effect of the intervention on preoperative HbA_1c_. If there is no discernible effect (defined as a change of HbA_1c_ of <5 mmol/mol), we will ask the trial steering committee to review the trial’s viability.

### Statistical analysis

Baseline participant demographics and characteristics will be summarised between the two arms.^17^ The primary analysis will be conducted using analysis of covariance (ANCOVA) adjusted for randomisation stratification factors on an intention-to-treat population. Continuous data will be presented as means and SD and analysed using ANCOVA (or presented as medians and ranges and analysed using Mann-Whitney U tests if data are skewed). Binary data will be reported in terms of ORs and analysed using logistic regression modelling. Analysis of time-to-event outcomes will include presenting Kaplan-Meier graphs by arm and analysed using Cox proportional hazards regression (or competing risk regression as discussed below). A two-sided p value of 0.05 or less will be used to declare statistical significance for all analyses and results will be presented with 95% CIs. Subgroups will be investigated, including those with HbA_1c_ above or below 69 mmol/mol at presentation, type of diabetes, age above or below 75 years. The cut-off of 69 mmol/mol has been chosen as the level above which the Joint British Diabetes Societies recommend specific action to improve preoperative glycaemic management. The cut-off for age has been chosen to reflect the different HbA_1c_ entry criteria for those above and below 75 years.

It is possible that a small proportion of participants will receive the intervention/usual care but will not actually undergo the planned surgery due to death, or clinically directed surgery cancellation. A small proportion may also undergo urgent revascularisation due to myocardial infarction after they have received their allocated treatment. A further group may undergo surgery but die before they are fit for discharge and thus not meet the primary endpoint. It is expected that these events will occur in fewer than 5% of participants. These individuals will be excluded from the primary analysis but the prevalence of each of these outcomes will be monitored and recorded by treatment arm separately to assess if there is an excess of any of these outcomes in either group. A sensitivity analysis will be considered, looking at a competing risks model, where these outcomes and functional recovery are competing risks. This sensitivity analysis will also be performed if the total prevalence of these events exceeds 5%.

### Economic evaluation

Quality-adjusted life years (QALYs) will be estimated from EQ-5D-5L and mortality data using the area-under-the-curve method. Similarly, costs will be estimated at the patient level. Mean between-group differences in QALYs and costs will be estimated using a regression-based approach, including adjustment for baseline covariates and interaction terms for predefined subgroups, and allowing for clustering at hospital and/or practitioner level. Results will be presented as an incremental cost-effectiveness ratio (ICER) if appropriate. Non-parametric bootstrapping will be used to estimate CIs around estimated cost differences and ICERs.

A simple modelling approach will also be used to estimate the costs and health impacts of surgical complications over a lifetime horizon. This extrapolation is necessary to reflect any mortality or lasting quality of life decrement associated with surgical complications. There will be no attempt to estimate the long-term impact of improved diabetes management related to the intervention, as it will be difficult to predict the duration over which any improvements will be maintained. This is likely to be a conservative assumption that will underestimate the QALY gain and cost-effectiveness of intervention if it proves effective. Model parameters will be estimated from the trial and from other published sources. Long-term resource use, mortality and utility decrements associated with key surgical complications will be identified by systematic review of HTAs, NICE guidelines and published literature.

### Qualitative and psychosocial evaluation

#### Interviews

Fifty participants receiving the intervention will be recruited across all participating sites balanced for age, gender, HbA_1c_, socioeconomic status and ethnicity. Baseline interviews will take place within 2 weeks of participants’ starting the intervention and follow-up interviews will be conducted with the same participants at 90 days postsurgery. Key personnel involved in the delivery will be interviewed once around 12 months after the start of trial in their centre.

Interview data analysis will include (1) comparisons between participants’ baseline and follow-up interviews to identify changes in their perceptions, experiences and diabetes self-management practices over time, and the reasons for these; (2) comparison of participant and health professional accounts to identify similarities and differences in their understandings and any impact on diabetes self-management practices; (3) cross-comparison of participants’ accounts to identify common issues and experiences as well differences in diabetes self-management practices between subgroups of participants (eg, men vs women, participants of different ages, etc), and the reasons for these.

#### Psychosocial questionnaires

The following questionnaires will be completed by participants at baseline and at 3 months postsurgery:

- Diabetes Empowerment Scale (short form): an 8-item questionnaire assessing diabetes-related psychosocial self-efficacy.
- PAID5: a 5-item self-reported measure of diabetes-related distress with high internal consistency.
- Patient Health Questionnaire (PHQ-2): ultra-brief depression screener, variant of PHQ-9. It is not used to establish a final diagnosis or to monitor depression severity but rather to screen for depression as a ‘first step’ approach.
- Brief Illness Perception Questionnaire (B-IPQ): an 8-item measure assessing cognitive illness representations, emotional representations, illness comprehensibility and perceived causal factors for illness.
- Summary of Diabetes Self-Care Activities scale (SDSCA): a 15-item self-report questionnaire of diabetes self-management that includes items assessing the following aspects of the diabetes regimen: general diet, specific diet, exercise, blood-glucose testing, foot care and smoking.

The analysis of the questionnaire responses will aim to answer the following questions:

1. What effect does baseline score (categorised as high/low, etc, as appropriate) have on study outcomes, that is, days until considered fit for surgery?
2. What effect does the study intervention have on change in score assessed as a continuous variable from baseline to 90 days postsurgery?
3. Does the treatment work better or less well in people depending on their baseline score (categorised)?

## Safety

Standard definitions and reporting procedures of adverse events, serious adverse events (SAEs), seriousness will be used (online supplemental material 3). For the purposes of this study, the following SAEs will not require reporting to Southampton Clinical Trials Unit:

10.1136/bmjopen-2021-050919.supp3 Supplementary data

- Hospitalisations for elective treatment of a pre-existing condition.

Also, the following SAEs will not require reporting if they occur between ‘Surgery’ and ‘Discharge’:

- Arrhythmia, including atrial fibrillation.
- Immediate postoperative surgical bleeding.
- Pneumonia.

Expectedness assessments are made against the list of expected events below:

- Minor musculoskeletal aches and pains.
- Myocardial infarction.
- Respiratory tract infection.

## Patient and public involvement

The trial has been developed in collaboration with the study patient and public involvement advisory group and local branch of Diabetes UK. The trial includes two patient representatives as a member of the Trial Steering Committee (TSC) and a member of the Trial Management group. Both individuals have been involved in the development of this protocol and have attended meetings regularly. To date, they have had an active role in assessing the study progress to date and both will be involved in resolving any issues that may arise.

## Ethics

Ethics approval was obtained by the South Central–Hampshire A Research Ethics Committee on 25 August 2020 (20/SC/0271). University Hospital Southampton NHS Foundation Trust will sponsor the study (RHM MED1718). The study is funded by the National Institute of Health Research *Health Technology Assessment* (*HTA*) Programme (16/25/12). The day-to-day management of the trial will be coordinated through the Southampton Clinical Trials Unit and oversight will be maintained by the Trial Steering Committee. The study will be conducted in accordance with WMA Declaration of Helsinki and as revised and recognised by governing laws and EU Directives.

All participants may withdraw at any time without providing a reason. Investigators will explain the value of remaining in study follow-up and allowing these data to be used for trial purposes. Where possible, those who have withdrawn from study treatment should remain in follow-up as per the trial schedule. If participants additionally withdraw consent for this, they will revert to standard clinical care. The study team will continue to collect standard follow-up data unless the participant explicitly states otherwise.

## Dissemination

Results will be disseminated through national and international conferences, scientific journals, newsletters, magazines and social media. Target audiences include diabetes specialist teams, cardiac surgeons, primary care team and medical professionals or scientists overall, as well as people with diabetes. This study addresses an important clinical question and is the first to assess whether early contact with a specialist diabetes team in the weeks leading up to surgery improves cardiothoracic surgical outcomes and reduces the excess morbidity and mortality experienced by people with diabetes. We further believe that the sample size and number of sites will mean that the results are sufficiently generalisable to broader cardiothoracic practice across the UK and internationally.

## Supplementary Material

Reviewer comments

Author's manuscript
