# Supplementary material for: The Optimising Cardiac Surgery ouTcOmes in People with diabeteS (OCTOPuS) randomised controlled trial to evaluate an outpatient pre-cardiac surgery diabetes management intervention: a study protocol
Source: BMJ Open. 2021 Jun 9;11(6):e050919. doi: 10.1136/bmjopen-2021-050919 (PMC8191627; doi:10.1136/bmjopen-2021-050919)
Supplement: Supplementary data [file bmjopen-2021-050919supp001.pdf]

Southampton Clinical Trials Unit

OCTOPuS

Optimising Cardiac Surgery ouTcOmes in People with diabeteS

OCTOPUS Intervention Manual

Version Number 6
Date: 9-Jun-2020

IRAS reference number: 283351

Holt RIG, *et al. BMJ Open* 2021; 11:e050919. doi: 10.1136/bmjopen-2021-050919

OCTOPuS Intervention Manual

Welcome to the OCTOPuS Intervention Manual

The OCTOPuS intervention has been designed for people with diabetes who have been listed for cardiac surgery involving a sternotomy. The aim of the intervention is to improve diabetes control in the run up to surgery, with the goal of improving the surgical and post-surgical experience. Although the focus of the intervention is to improve glucose control, the intervention also includes the management of other aspects of diabetes, such as weight, that are known to affect surgical outcomes.

The intervention begins once an individual has been accepted for cardiac surgery and continues until the individual has had their cardiac surgery or the surgery is cancelled. Following discharge or if the surgery is cancelled, an individual's diabetes care will revert to their usual care prior to listing for surgery.

The OCTOPuS intervention will be tested in a randomised controlled trial, funded by the National Institute for Health Research Health Technology Assessment programme.

It is hoped that if the intervention is successful in improving surgical outcomes, it can be implemented in the National Health Service and adapted for other major surgery.

Southampton Clinical Trials Unit

Change Control

Date	Version	Activity	Who
**19th Dec 2018**	2	Clarification on how many support calls are required.	Liz Dixon
**15th August 2019**	3	Clarification on timing of intervention and managing surgical queries.	Liz Dixon
**14th October 2019**	4	1. Additional support call post-surgery<br>2. Note regarding retinopathy	Giorgos Dritsakis, Richard Holt
**16th January 2020**	5	Changes regarding frequency of telephone calls and HbA1c measurement	Richard Holt
**9th June 2020**	6	Addition of remote initial consultation option	Richard Holt

Southampton Clinical Trials Unit

Contents

1 ABOUT OCTOPUS AND THIS MANUAL 5

1.1 WHAT IS THE OCTOPUS INTERVENTION? 5
1.2 WHY IS THE OCTOPUS INTERVENTION NEEDED? 5
1.3 WHO IS THE OCTOPUS INTERVENTION FOR? 6
1.4 ABOUT THIS MANUAL 6

2 THE OCTOPUS INTERVENTION 7

2.1 THE OCTOPUS PRACTITIONER 7
2.2 GLUCOSE MANAGEMENT 7
2.2.1 Glucose monitoring 8
2.2.2 Lifestyle modification 9
2.2.3 Drug therapy for people with type 2 diabetes not currently receiving antidiabetes medication 10
2.2.4 People with type 2 diabetes currently receiving oral antidiabetes medication 11
2.2.5 People with type 2 diabetes already on insulin 11
2.2.6 People with type 1 diabetes 11
2.2.7 Summary of antidiabetes medication options 12
2.2.8 Diabetes management during admission for cardiac surgery 14
2.3 LIPID MANAGEMENT 15
2.4 HYPERTENSION MANAGEMENT 15
2.5 WEIGHT MANAGEMENT 16
2.6 EXERCISE 16
2.7 SMOKING CESSATION 17
2.8 INVOLVEMENT OF SPOUSES, OR OTHER RELATIVES AND FRIENDS 17

3 DELIVERING THE OCTOPUS INTERVENTION 18

3.1 THE SCHEDULE OF EVENTS 18
3.1.1 The Initial Assessment 18
3.1.2 The Fortnightly Phone Call 20
3.1.3 Surgery and Beyond 21

APPENDIX 1: THE OCTOPUS RANDOMISED CONTROLLED TRIAL 22

APPENDIX 2: BIBLIOGRAPHY 23

Figures

Figure 1 - Diabetes medication flowchart for people with type 2 diabetes; based on the 2018 American Diabetes Association care standards 12
Figure 2 - A high-level flowchart of the OCTOPuS Intervention 18

Tables

Table 1 - Framework for Initial Assessment 19
Table 2 - Framework for Fortnightly Phone Call 20

1 About OCTOPuS and this manual

1.1 What is the OCTOPuS Intervention?

The OCTOPuS intervention is a set of elements, designed to improve the management of a person with diabetes over the weeks preceding scheduled major surgery. The components of the OCTOPuS intervention represent current best clinical practice and are endorsed by NICE or other guidelines. Suitably qualified and trained clinicians will deliver OCTOPuS.

The planned randomised evaluation (see Appendix 1) has been designed to assess the use of the OCTOPuS intervention for people with inadequately controlled diabetes undergoing cardiothoracic surgery, and this manual assumes that setting.

1.2 Why is the OCTOPuS intervention needed?

There are approximately 4 million people living with diagnosed and undiagnosed diabetes mellitus in the UK. Since 1996, the number of people diagnosed with diabetes has increased from 1.4 million to around 3.5 million. Diabetes increases the risk of cardiovascular disease by approximately two fold after adjustment for other cardiovascular risk factors. Ischaemic heart disease is by far the leading cause of death in people with diabetes accounting for approximately two-thirds of all deaths in those aged >65 years. Coronary heart disease tends to be more diffuse and progresses more rapidly in people with diabetes, which may explain why up to 35% of those presenting for elective cardiac revascularisation have diabetes.

The increasing number of people with diabetes will increase the demand for cardiac surgery in the future. These individuals have longer lengths of hospital stay and higher re-admission rates, placing a large financial burden on the NHS.

Poor glycaemic control increases the risk of wound and chest infections, renal impairment and death, especially following cardiac surgery. The Joint British Diabetes Societies for in-patient care has provided recommendations to improve the management of adults with diabetes undergoing surgery. As poor peri-operative glycaemic control is associated with an increased risk of all surgical complications, the guidelines recommend improving glycaemic control to optimise surgical outcomes.

Hyperglycaemia, however, does not wholly explain poorer surgical outcomes of people with diabetes; other important risk factors, such as obesity, hypertension and dyslipidaemia, are also more common in people with diabetes. Furthermore, lack of knowledge and training of the nursing and medical teams might also contribute.

If the pre-operative intervention is successful in improving glycaemic control and addressing other risk factors, this may reduce the complication rate and improve the clinical outcomes. It may also prove cost effective and even cost saving.

1.3 Who is the OCTOPuS intervention for?

The intervention can be offered to any person with sub-optimally controlled diabetes, which is defined as an HbA_{1c} >53 mmol/mol (>7%) using a point-of-care test at the cardiothoracic outpatient appointment where the decision to proceed to surgery is made.

The intervention described here assumes there is a period of at least 10 to 12 weeks before the scheduled surgery, but patients may derive benefit from a shorter intervention. In some circumstances, surgery may be delayed beyond 12 weeks. In this instance the pre-operative OCTOPuS intervention should continue until the patient is admitted surgery.

OCTOPuS is not suitable for people with malignancy, women who are pregnant or those with other illnesses or conditions that would preclude engagement with the intervention.

1.4 When should this manual be used?

The OCTOPuS intervention should begin as soon as possible after an individual has been accepted for cardiac surgery. The intervention continues until the individual has had their cardiac surgery or the surgery is cancelled. Following discharge or if the surgery is cancelled, an individual's diabetes care will revert to their usual care prior to listing for surgery.

1.5 About this manual

This manual describes the OCTOPuS intervention. At the time of writing, the intervention is still under development. The latest version of the manual can be obtained from the SCTU website (www.southampton.ac.uk/ctu) or from the OCTOPuS trial manager (octopus@soton.ac.uk).

This section has described the background and justification for the intervention.

Section 2 describes the components of the intervention, with a discussion of how each component might be delivered individually.

Section 3 discusses how these components are brought together and delivered as a coherent intervention.

2 The OCTOPuS Intervention

In this section, we describe the individual components of the intervention. How these components are tied together and delivered to the patient is described in section 3.

The OCTOPuS intervention comprises several elements, which are brought together in a systematic way. It is the role of the OCTOPuS practitioner to work with the patient awaiting surgery to decide which elements of the programme are applicable to the individual.

The recommendations in this manual represent the views of the OCTOPuS research team and are presented after careful consideration of the evidence and currently available NICE and international guidelines. When making treatment decisions with the participants, OCTOPuS practitioners are expected to take this manual into account, alongside the individual needs, preferences and values of their trial participants. It is not mandatory to apply the recommendations in the manual, and the guideline does not override the responsibility to make decisions appropriate to the circumstances of the individual, in consultation with them and their families and carers or guardian.

Given the short duration of the intervention and limited number of contacts, it may not be feasible to implement all actions and changes suggested by this manual. The OCTOPuS practitioner should work with the patient to implement as much as possible, planning to bring the patient to the best clinical status prior to surgery, but not delaying the planned surgical procedure in order to deliver OCTOPuS.

The sections below provide guidance in how to decide which elements might benefit each individual and guide the decision-making process. In general, the management below should follow NICE guidance for the management of diabetes, hypertension, dyslipidaemia and obesity unless specifically described otherwise.

2.1 The OCTOPuS practitioner

The OCTOPuS practitioner is a clinically qualified health care worker with expertise in diabetes. They are most likely to be a diabetes nurse specialist, but might, for example, be a pharmacist, dietitian or physician. The OCTOPuS practitioner will receive additional specific training about the OCTOPuS intervention.

Once a plan has been agreed, the practitioner supports the patient through regular contact (at least fortnightly until optimised), encouragement and counselling, signposting, and referral to key services.

Each practitioner will work with several patients awaiting surgery, providing initial advice, then remote telephone follow-up over the 3 months or so until the cardiac procedure. The OCTOPuS practitioner will need to provide advice about medication regimens, direct patients to local services, to advocate on patient’s behalf, and to provide a listening ear to the patient.

2.2 Glucose Management

All patients eligible for the intervention will have an HbA_{1c} of >53mmol/mol (7.0%) and may benefit from improved glucose control. The challenge is to improve control without inducing episodes of hypoglycaemia, or excessive weight gain. The decision to intensify therapy should be made on an individual basis; for example, a more relaxed target may be appropriate for a

person with significant co-morbidities or where the risk and consequence of hypoglycaemia are high. There is a risk of worsening retinopathy in people with existing retinopathy if there is a rapid decline in HbA1c. The OCTOPuS practitioner should enquire whether the participant has a history of retinopathy and whether their retinal screening is up-to-date. The OCTOPuS practitioner should consider retinopathy when discussing treatment changes.

Improving glucose control may require lifestyle intervention, medication change or a combination of these strategies. How they can be delivered will be determined by local policies and funding. If the OCTOPuS practitioner is able to prescribe then they may undertake any initial changes themselves; otherwise, they should make recommendations to the patient's GP or local specialist diabetes team.

The patient's diabetes management and medication should be considered at every OCTOPuS interaction. The process is outlined in this section and summarised in Figure 1 on page 13. The OCTOPuS practitioner should provide the patient with a Diabetes UK Information Prescription about glycaemic management at each visit, if appropriate, to support glycaemic management.

2.2.1 Glucose monitoring

If the patient is not already monitoring their blood glucose, the OCTOPuS practitioner should provide the patient with a capillary glucose monitor and sufficient strips for the duration of the intervention (approximately 100 strips). They should teach the patient how to monitor and interpret their glucose using a standardised education package. All participants with Type 2 diabetes should be given a copy of 'Your Guide to Type 2 Diabetes' education booklet produced by Diabetes UK to keep for their personal reference and use throughout the study. Where applicable, participants should be shown online software management systems such as the Accu-Chek 360° Diabetes Management System. Although glucose monitoring is not usually recommended for routine use in people not using insulin or sulfonylureas, this is an important component of the intervention as short-term improvements in glucose control may not be apparent from changes in HbA_{1c} because of the short duration of the intervention.

In addition to any glucose monitoring that the patient is already undertaking prior to the intervention, patients should be advised to check their glucose levels 4 times a day (before meals and before bed) on the 3 days prior to the next OCTOPuS contact. The OCTOPuS practitioner should provide the patient with a glucose and diet diary so that the results can be recorded prior to the consultation. The OCTOPuS practitioner should encourage the patient to record what they eat in the diary so that any relationship between glucose readings and reported diet can be discussed at the consultation.

As most consultations will be remote, e.g. by telephone or Skype, where possible, the results should be sent to the OCTOPuS practitioner before the consultation, e.g. by email or Diasend (where available).

Following discussion between the OCTOPuS practitioner and patient, the glucose targets should be individualised, taking into account the risk of hypoglycaemia. However, typical glucose targets would be:

- Fasting and pre-meal glucose values: 4.0-6.0 mmol/L
- Post-meal and before bed glucose values: 4.0-7.8 mmol/L (for people using insulin who are prepared to test after meals)

2.2.2 Lifestyle modification

Dietary and exercise advice should follow the 2018 Diabetes UK nutritional guidelines (https://www.diabetes.org.uk/professionals/position-statements-reports/food-nutrition-lifestyle/evidence-based-nutrition-guidelines-for-the-prevention-and-management-of-diabetes). Where necessary, a healthcare professional with specific expertise and competencies in nutrition should see the patient.

Encourage high-fibre, low-glycaemic-index sources of carbohydrate in the diet, such as fruit, vegetables, wholegrains and pulses; include low-fat dairy products and oily fish; and control the intake of foods containing saturated and trans fatty acids. Encourage the patients to avoid excessive alcohol consumption.

The following leaflets may be of benefit:

British Heart Foundation:
Food labelling guidance: https://www.bhf.org.uk/publications/healthy-eating-and-drinking/this-label-could-change-your-life

Weight Loss advice:
https://www.bhf.org.uk/publications/healthy-eating-and-drinking/facts-not-fads---your-simple-guide-to-healthy-weight-loss

Nutrition and Diet Resource
Weight loss advice: describes 80kcal portions of different food groups so that the participants can decide how many of each they can have i.e. 1500kcal
https://www.ndr-uk.org/item/81/WeightManagement/Weight-Loss-You-Can-See-with-guidelines.html
This is a paid resource but could be useful for more visual patients

Carbs and cals – a variety of recipes, carb values, and low calorie meal options:
https://www.carbsandcals.com/weight-loss/weight-loss

British Dietetic Association:
Basic diet sheets for glycaemic index, healthy eating and weight loss
https://www.bda.uk.com/foodfacts/GIDiet.pdf
https://www.bda.uk.com/foodfacts/HealthyEating.pdf
https://www.bda.uk.com/foodfacts/Want2LoseWeight.pdf

For individuals who are overweight or obese, weight loss should be encouraged (see below).

The OCTOPuS practitioner should encourage the patient to become more physically active (see below).

2.2.3 Drug therapy for people with type 2 diabetes not currently receiving anti-diabetes medication

Almost all patients will need anti-diabetes drug therapy because of the elevated HbA_{1c} and short window-of-opportunity to improve the glucose control. The OCTOPuS practitioner should use their clinical judgement in the choice of medication, which should be individualised in discussion with the patient. All prescribing advice should be within the drug licence and take account of the individual Summary of Product Characteristics.

In line with NICE and 2018 American Diabetes Association guidance, the first line treatment of choice is standard release metformin, unless contraindicated. Metformin should be used according to its licence. Slow release metformin can be considered if the standard released metformin is not tolerated.

Where the initial HbA_{1c} is ≥75 mmol/mol (9%), consideration should be given to starting dual therapy from the outset as recommended by the 2018 American Diabetes Association guidance.

If metformin is insufficient to achieve adequate glycaemic control (as judged by capillary glucose monitoring described above), a second agent should be added. Because of the short-time scale of the intervention, it is not possible to base treatment changes on the measurement of HbA_{1c} for most patients. As all patients will have existing atherosclerotic cardiovascular disease, in line with the 2018 American Diabetes Association guidance, the first treatment intensification should usually be with a drug that has proven cardiovascular benefit, e.g. an SGLT-2 inhibitor (e.g. empagliflozin or canagliflozin) or a GLP-1 receptor agonist (e.g. liraglutide), unless contraindicated. The second treatment intensification should be the other class of drug (i.e. if an SGLT2 inhibitor was the first intensification then the second should be GLP-1 receptor agonist or vice versa), unless contraindicated. OCTOPuS practitioners will need to take account of drug-specific and patient factors as well as local formulary requirements.

The 2018 American Diabetes Association guidance diverges from current NICE guidance as the latter has not yet been updated in light of the latest cardiovascular outcome trials. However, there is a need to avoid weight gain and hypoglycaemia in this group of patients and there is a need to avoid provoking cardiovascular events as occurred in the ACCORD study.

Where these drugs are contraindicated, alternative agents, such as DPP-4 inhibitors, pioglitazone or sulfonylureas, e.g. gliclazide, can be used according to NICE guidance.

If adequate glycaemic control is not achieved with three non-insulin therapies, insulin should be initiated according to NICE guidance (see below). Where the initial HbA_{1c} is ≥86 mmol/mol

(10%), consideration should be given to starting insulin therapy from the outset as recommended by the 2018 American Diabetes Association guidance.

2.2.4 People with type 2 diabetes currently receiving oral anti-diabetes medication

Lifestyle factors, such as diet and physical activity, should be explored but it is likely that drug therapy will need to be intensified.

The OCTOPuS practitioner should explore whether the patient is taking the medication as prescribed. It is known that fewer than 50% of people receiving oral anti-diabetes treatments, antihypertensive agents and statins persist with their medication 2 years after treatment initiation and up to 20% never start treatment. The barriers to adherence should be discussed with the patient.

Where drug therapy intensification is needed, this should be done as described in the previous section 2.2.3. If the individual is on other combinations of oral anti-diabetes agents, the OCTOPuS practitioner should consider whether to change these to treatments with proven cardiovascular benefit.

2.2.4.1 Commencement of Insulin in people with type 2 diabetes

If adequate glycaemic control (judged by capillary glucose monitoring or presenting HbA_{1c}) is not achieved with three non-insulin therapies, insulin should be initiated according to NICE guidance. Basal insulin alone is the most convenient initial insulin regimen, beginning at 10 units per day or 0.1–0.2 units/kg/day, depending on the degree of hyperglycaemia.

As hypoglycaemia is a major risk factor for cardiovascular events, the OCTOPuS practitioner should have a low threshold to initiate insulin analogues instead of NPH insulin because of the lower risk of hypoglycaemia seen with the use of insulin analogues.

The OCTOPuS practitioner will need to liaise with local health services to ensure that the patient is offered sufficient training to use the insulin effectively. Given the urgency of treatment, in many instances, this will need to be outside usual channels.

Intensification of insulin is usually by the addition of prandial insulin or switch to pre-mixed insulin. The options should be discussed with the patient and a management plan agreed with the patient.

2.2.5 People with type 2 diabetes already on insulin

The OCTOPuS practitioner should review the current insulin regimen, injection technique and sites with the patient. The OCTOPuS practitioner should offer advice about the doses and types of injection as necessary.

2.2.6 People with type 1 diabetes

It is likely that the OCTOPuS practitioner will see people on a variety of insulin regimens, including both multiple daily injection and insulin pump therapy. The OCTOPuS practitioner

should review the current insulin regimen, injection technique and sites with the patient. The OCTOPuS practitioner should offer advice about the doses and types of injection as necessary.

If the patient has not attended a structured education course, this should be offered where possible.

A detailed description of insulin therapy is beyond the scope of this manual and the OCTOPuS practitioner should refer to the NICE type 1 diabetes guidance.

2.2.7 Summary of anti-diabetes medication options

The OCTOPuS practitioner should provide the patient with a Diabetes UK Information Prescription about glycaemic management at each visit to support glycaemic management.

Figure 1 summarises the options available to improve glycaemic control prior to surgery. The OCTOPuS practitioner should discuss progress with the patient at every visit or fortnightly phone call and management plan adjusted accordingly.

Holt RIG, *et al*. *BMJ Open* 2021; 11:e050919. doi: 10.1136/bmjopen-2021-050919

Southampton Clinical Trials Unit

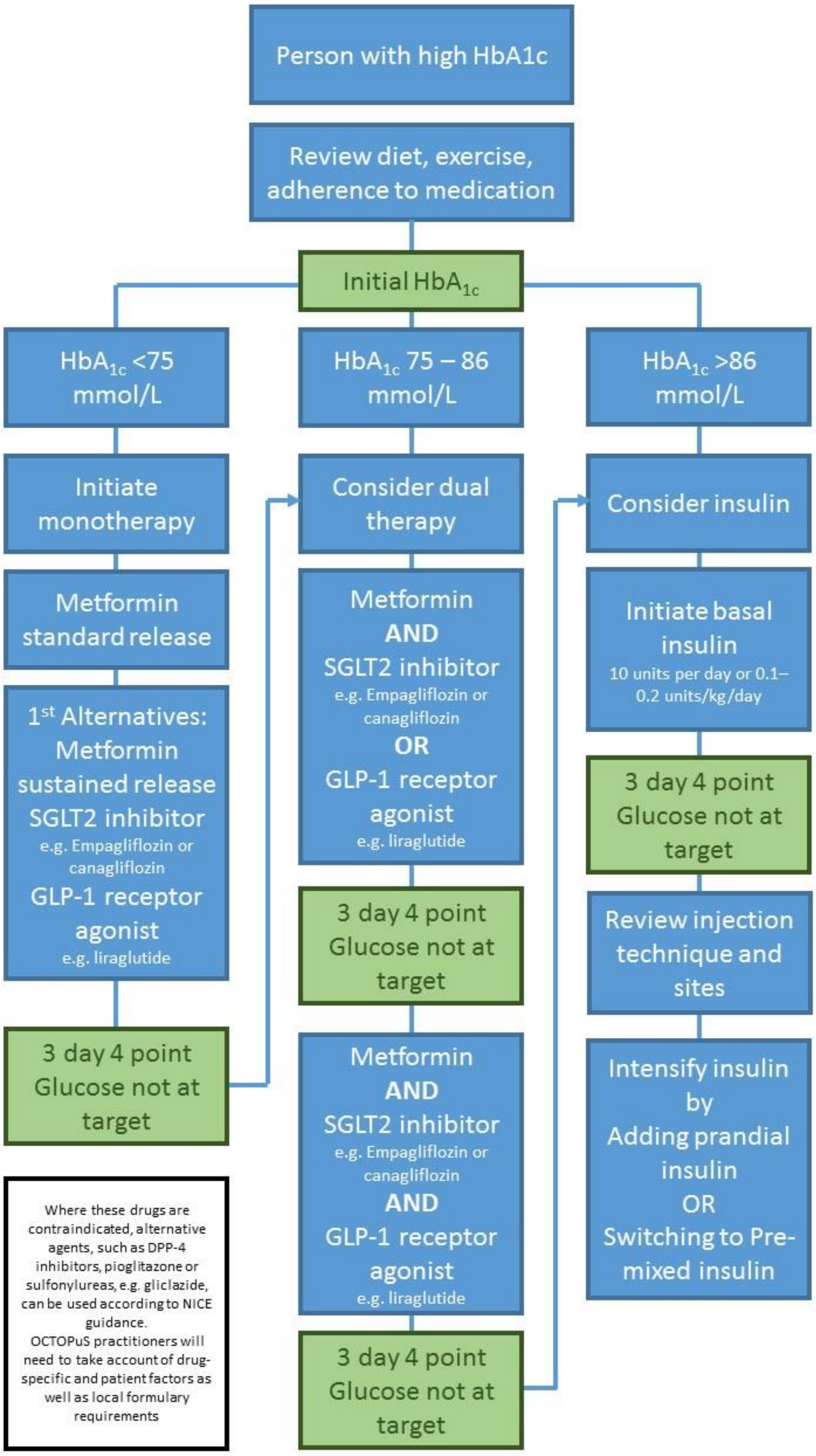

Figure 1 - Diabetes medication flowchart for people with type 2 diabetes; based on the 2018 American Diabetes Association care standards.

Holt RIG, *et al. BMJ Open* 2021; 11:e050919. doi: 10.1136/bmjopen-2021-050919

2.2.8 Diabetes management during admission for cardiac surgery

The OCTOPuS practitioner will need to provide advice about medication and glucose management when the patient is admitted for surgery. Where possible, the OCTOPuS practitioner should arrange to see the patient after admission but prior to surgery to provide advice about diabetes management during the admission.

The OCTOPuS practitioner may need to liaise with the diabetes in-patient team in the cardiothoracic centre, if not already a member of this team. Local protocols and JBDS guidance should be followed (http://www.diabetologists-abcd.org.uk/JBDS/Surgical_guidelines_2015_full_FINAL_amended_Mar_2016.pdf). There may be specific questions about cardiac surgery or management of diabetes during the operation that the OCTOPuS practitioner is unable to answer. If this is the case, the OCTOPuS practitioner should alert the cardiac surgeon so that the surgical team can answer these questions.

The OCTOPuS practitioner should advise the patient to continue to monitor their glucose, where appropriate. They should warn the patient of the risk of hypoglycaemia during fasting prior to admission. The OCTOPuS practitioner should advise the patient that oral anti-diabetes medications and GLP-1 receptor agonists need to be omitted on the day of surgery. The OCTOPuS practitioner should provide advice about adjustments to insulin doses. Suggested adjustments to insulin doses are as follows:

**Insulin**	**Day before procedure**	**Day of procedure**
Once daily (evening) (e.g. Lantus, Levemir, Tresiba, Abasalaglar Insulatard or Humulin I, Toujeo)	Take 80% of usual insulin dose at usual time.	Take 80% of usual insulin dose in the evening after the procedure
Once daily (morning) (e.g. Lantus, Levemir, Tresiba, Insulatard or Humulin I)	Take usual time.	Take 80% of usual insulin dose in the evening after the procedure
Twice daily (e.g. Novomix 30, Humulin M3, Humalog Mix 25 or 50, Lantus, Levemir)	Take usual time	Omit morning dose
Meal time injection	Take usual time	Omit all rapid insulin
Insulin pump	Please inform specialist pump team before admission for personalised advice. Continue with usual basal rates and continue to bolus depending on carbohydrate intake	Continue with usual basal rates and continue to bolus depending on carbohydrate intake

Following surgery but prior to discharge, the OCTOPuS practitioner should see the patient to discuss post-operative diabetes management. In some situations, it may be appropriate to discontinue insulin therapy. Any treatment changes and on-going management plans should be communicated to the patient's GP and specialist diabetes team, if necessary, following discharge after surgery. This information could be included in the discharge summary or communicated separately depending on local arrangements.

2.3 Lipid Management

Most patients will already be taking lipid-lowering therapy. However, if they are not taking a statin, the OCTOPuS practitioner should discuss the benefits of statin therapy and recommend that this is initiated if there are no contraindications or the person has previously not tolerated treatment with statins.

For those already taking lipid-lowering therapy, the OCTOPuS practitioner should review the patient's latest lipid profile. If a greater than 40% reduction in non-HDL cholesterol has not been achieved, the OCTOPuS practitioner should discuss adherence and timing of dose, optimise adherence to diet and lifestyle measures and consider increasing the statin dose if the participant is taking less than atorvastatin 80 mg. The OCTOPuS practitioner should consider the addition of ezetimibe. In some circumstances, these measures may be insufficient to control the lipid profile and in this situation, the OCTOPuS practitioner should considering recommending a referral to a specialist lipid clinic for consideration of PCSK9 inhibitors if this is available within the timeframe of the intervention.

Where changes have been recommended, the OCTOPuS practitioner should provide the patient with a Diabetes UK Information Prescription about lipid management.

2.4 Hypertension Management

Hypertension and endothelial dysfunction are common in people with diabetes. The OCTOPuS practitioner should measure the blood pressure or record the clinic blood pressure measurement as part of the initial assessment.

Preliminary data suggest that preoperative use of an antagonist of renin-angiotensin system (ACE inhibitor or angiotensin receptor blocker) in people undergoing CABG is associated with decreased in-hospital mortality. Unless contraindicated, the OCTOPuS practitioner should consider an antagonist of renin-angiotensin system (ACE inhibitor or angiotensin receptor blocker) for all patients after discussion with the local cardiothoracic surgical team. The dose should be titrated against the patient's blood pressure, which should be measured in the patient's general practice or by the patient at home. Further agents may be added in accordance with NICE guidance (CG127) as necessary. When an ACE inhibitor or angiotensin receptor blocker is added, the OCTOPuS practitioner should advise the measurement of urea and electrolytes and estimated glomerular filtration rate according to standard clinical

practice. At each contact, the OCTOPuS practitioner should advise the patients to withhold ACE inhibitors or angiotensin receptor blockers from 5 days prior to surgery.

Bear in mind that the surgical team may wish to commence a beta blocker prior to surgery, so if a second line agent is needed the OCTOPuS practitioner should consider commencing a beta blocker. This decision should be discussed with the local cardiothoracic surgical team.

Where changes have been recommended, the OCTOPuS practitioner should provide the patient with a Diabetes UK Information Prescription about blood pressure management.

2.5 Weight Management

Obesity and being overweight are associated with poor surgical outcomes. People with a BMI >25 Kg/m^2 are therefore likely to benefit from weight reduction, both to improve their diabetes control and surgical outcome.

The OCTOPuS practitioner should measure the patient's height and weight and calculate their BMI.

For recommendations on weight management, see the NICE guidelines on: preventing excess weight gain, weight management, and obesity. For most patients undertaking OCTOPuS, the major element of weight reduction will be through diet. Exercise is discussed in section 2.6 below.

The OCTOPuS practitioner should refer the patient or facilitate referral to a local NHS weight reduction programme or dietitian if this can be accessed quickly enough to achieve a worthwhile effect before surgery (e.g. the programme can be started within 4 weeks).

As described above, Diabetes UK and the British Heart Foundation both produce excellent leaflets about healthy eating and the OCTOPuS practitioner should provide the patient with a copy of these or let the patient know how to access them.

If an NHS option is not available, then other weight reduction options should be explored. This could include commercial programmes, such as Weight Watchers, or a self-managed diet.

2.6 Exercise

Physical activity has profound benefits for people with diabetes, including improved fitness, reduced insulin requirement and better glycaemic control, lower cardiovascular risk (lower blood pressure and improved lipid profile) and improved survival. People with diabetes should take at least 150 minutes of exercise per week spread over a minimum of 3 days with a mixture of aerobic exercise and resistance training.

However, care is needed in this group of patients as those awaiting cardiac revascularisation surgery may experience angina on exertion and have a limited capacity to provide oxygenated blood to cardiac muscle. Similarly, those awaiting valve surgery may not have significant capacity to exercise. Therefore, this component of the intervention will require the OCTOPuS practitioner to tailor any recommendation to the capacity of the individual.

It is also important to ascertain whether the individual has diabetes microvascular complications before starting to exercise. People with a history of active foot ulcers should avoid weight-bearing exercise and appropriate footwear should be worn.

However, where possible, the OCTOPuS practitioner should encourage patients to take gentle exercise, such as walking or dancing. The use of pedometers can support a gradual increase in physical activity. The best exercise is the one that the person enjoys. Practical advice should be given to help the person with diabetes find ways to become more physically active.

In the case of Type 1 diabetes, exercise can lead to unstable glucose levels during and immediately after exercise and a later risk of severe hypoglycaemia. Patients should be advised to avoid exercise of this intensity. As prior hypoglycaemia blunts the catecholamine response, people should be advised to avoid exercise within 24 hours of a severe hypoglycaemic episode and 1 hour of a self-treated episode. Exercise should also be avoided if blood ketone levels are increased. The OCTOPuS practitioner should consider whether a referral to a consultant diabetologist is required to address this complex area.

In type 2 diabetes, exercise does not usually cause hypoglycaemia and so carbohydrate supplementation is not required.

2.7 Smoking Cessation

All patients should be encouraged and supported to stop smoking, where possible. For recommendations on smoking cessation, see the NICE guidelines on: smoking: brief interventions and referrals, stop smoking services, and smoking: harm reduction.

2.8 Involvement of spouses, or other relatives and friends

The involvement of people important in the patient's life may lead to greater adherence to the components of the OCTOPuS intervention. Encouragement and support from friends and family will make the changes in medication, diet, and other activity, more sustainable over the few weeks that the intervention is delivered.

Therefore, if possible friends and relatives should be involved in the initial consultation, and consideration should be given to including them in the fortnightly phone calls.

3 Delivering the OCTOPuS Intervention

The elements described in section 2 are brought together in the OCTOPuS intervention.

3.1 The Schedule of Events

The intervention follows a series of events, shown in Figure 1. At each stage, the practitioner works with the patient to agree a set of goals and actions.

Figure 1 - A high-level flowchart of the OCTOPuS Intervention

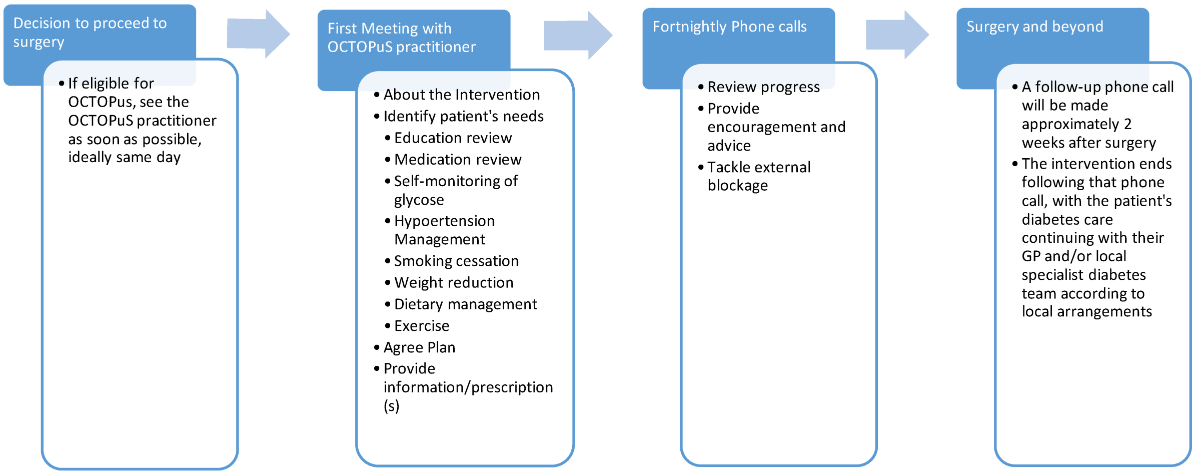

3.1.1 The Initial Assessment

The initial assessment is key to the OCTOPuS intervention. It is where the practitioner establishes a trust relationship, which will enable the patient to take the greatest advantage of the programme. We anticipate that the initial consultation will be usually be conducted face-to–face. However, alternative remote delivery options (video or telephone consultations) may be considered when these are necessary, for example, when the patient is unable to attend the hospital or to comply with COVID-19 social distancing.

Table 1 - Framework for Initial Assessment

Phase	Activity
**Assessment**	Explore the patient's understanding and experience of diabetes.<br>Explore any concerns the patient has relating to diabetes and their prospective surgery.<br>Establish whether there are relevant co-morbidities<br>Establish any significant people in the patient's life who could provide support in the run up to surgery.
**Explanation**	Explain the OCTOPuS intervention, and its goals, in the light of information elicited in the assessment phase.
**Measurement**	Make any clinical assessments which may be required, that weren't done in the most recent cardiovascular outpatient appointment<br>• HbA_{1c}<br>• Blood Pressure<br>• Height, Weight -> BMI
**Review**	Review the patient's situation in the areas set out in section 2 of this manual.<br>• Glucose Management<br>• Hypertension Management<br>• Weight Reduction<br>• Smoking Status<br>• Exercise<br>Agree a plan, where appropriate, with the patient for each of these elements
**Follow-up**	Schedule the first fortnightly phone call. You may like to schedule multiple calls for the complete period up to the planned surgery date.<br>Obtain permission from the patient to contact their GP or other services to support the patient's action plan. Liaise with local clinical team where necessary<br>Put into place any arrangements needed to support the patient's action plan

Holt RIG, *et al. BMJ Open* 2021; 11:e050919. doi: 10.1136/bmjopen-2021-050919

3.1.2 The Fortnightly Phone Call

At least every fortnight, the OCTOPuS practitioner will contact the patient until the patient's diabetes management is optimised or no further changes are possible. After this has been achieved, the frequency of the calls can be reduced at the discretion of the OCTOPuS practitioner to a minimum of every 6 weeks. We envisage this being by phone but other methods (face-to-face, Skype etc.) could be used by mutual agreement.

This is an opportunity for the practitioner to review progress against goals, and for the patient to raise any queries they might have.

The OCTOPuS practitioner should ensure that the patient has the contact details of the OCTOPuS team so that they can contact the team if necessary.

Table 2 - Framework for Fortnightly Phone Call

Phase	Activity
**Assessment**	Explore the patient's activity and progress against the goals agreed at the previous initial assessment or fortnightly phone call<br>Explore whether there has been any changes health, e.g. infection, that might affect the management plan.
**Measurement**	Make any clinical assessments, which may be required. This will generally involve the patients reporting over the phone or by alternative means of communication, such as email, DIASEND, Freestyle Libreview etc.<br>• Recent self-monitoring of blood glucose<br>• Weight<br>• Smoking status
**Listen**	To any problems or concerns that the patient raises

Phase	Activity
**Review**	Review the patient's situation in the areas set out in section 2 of this manual.<br>• Glucose Management – where surgery is delayed beyond 3 months, the OCTOPuS practitioner should arrange for a further HbA1c measurement every 3 months<br>• Hypertension Management<br>• Weight Reduction<br>• Smoking Status<br>• Exercise<br>Agree a plan, where appropriate, with the patient for each of these elements, in the light of the review and listening phases.<br>Liaise with local clinical team where necessary
**Follow up**	Schedule the next fortnightly phone call, if not already done.

3.1.3 Surgery and Beyond

Surgery will proceed according to local protocols.

Approximately two weeks after surgery, the OCTOPuS practitioner will contact the patient again. This phone call will follow the framework in Table 2 above. During the final support call, the OCTOPuS practitioner should undertake a review of the elements of the intervention and develop a future diabetes management plan.

All patients who have completed the OCTOPuS intervention and their surgical treatment should then return to routine diabetes care with their GP or local diabetes specialist team.

Appendix 1: The OCTOPuS Randomised Controlled Trial

The OCTOPuS trial has been funded by the NIHR HTA programme to evaluate the OCTOPuS intervention, and to determine whether it adds value to patient care.

Approximately 426 people with poorly controlled diabetes undergoing cardiac surgery will be randomised to either the OCTOPuS intervention or to usual care. The outcomes of interest include time from surgery until clinically for hospital discharge, 30 & 90 day mortality, wound infections, chest infections, renal impairment, HbA_{1c} pre-op and, 90 days post op, cost-effectiveness, procedures cancelled due to glycaemic control, quality of life, patient satisfaction & experience.

More information is available at
https://www.journalslibrary.nihr.ac.uk/programmes/hta/162512/#/

Appendix 2: Bibliography

1 Zheng J, Cheng J, Wang T, *et al.* Does HbA1c Level Have Clinical Implications in Diabetic Patients Undergoing Coronary Artery Bypass Grafting? A Systematic Review and Meta-Analysis. *Int J Endocrinol* 2017;**2017**:1537213–8. doi:10.1155/2017/1537213

2 Rollins KE, Varadhan KK, Dhatariya K, *et al.* Systematic review of the impact of HbA1c on outcomes following surgery in patients with diabetes mellitus. *Clinical Nutrition* 2016;**35**:308–16. doi:10.1016/j.clnu.2015.03.007

3 Boreland L, Scott-Hudson M, Hetherington K, *et al.* The effectiveness of tight glycemic control on decreasing surgical site infections and readmission rates in adult patients with diabetes undergoing cardiac surgery: A systematic review. *Heart Lung* 2015;**44**:430–40. doi:10.1016/j.hrtlng.2015.06.004

4 Cheng X, Tong J, Hu Q, *et al.* Meta-analysis of the effects of preoperative renin–angiotensin system inhibitor therapy on major adverse cardiac events in patients undergoing cardiac surgery. *Eur J Cardiothorac Surg* 2015;**47**:958–66. doi:10.1093/ejcts/ezu330

5 NICE. *Hypertension in adults: diagnosis and management*. 2011.

6 NICE. *Preventing excess weight gain*. NICE 2015.

7 NICE. *Weight management: lifestyle services for overweight or obese adults*. NICE 2014.

8 NICE. *Obesity: identification, assessment and management*. NICE 2014.
