# Supplementary material for: The Optimising Cardiac Surgery ouTcOmes in People with diabeteS (OCTOPuS) randomised controlled trial to evaluate an outpatient pre-cardiac surgery diabetes management intervention: a study protocol
Source: BMJ Open. 2021 Jun 9;11(6):e050919. doi: 10.1136/bmjopen-2021-050919 (PMC8191627; doi:10.1136/bmjopen-2021-050919)
Supplement: Supplementary data [file bmjopen-2021-050919supp002.pdf]

PROGRESS GRADING

Actions to be taken depending on progress grade

Grade	Action
Green	Continue trial, keeping an eye on accrual.
Amber	Working with governance committees (TSC, TMG, PPI Committees), seek root cause for under performance. Consider whether these can be mitigated through work with organisations or individuals within the study.
Red	Review the study with governance committees, taking steps as detailed under amber, but also explicitly considering recommending study closure.

Progress grading time points and criteria

Assessment Point	Green	Amber	Red
After 100 patients have had surgery (50 intervention and 50 control)	HbA_{1c} reduction in intervention group >5mmol/mol	HbA_{1c} reduction in intervention group <5mmol/mol	HbA_{1c} reduction in intervention group not consistent with physiological effect

Holt RIG, *et al. BMJ Open* 2021; 11:e050919. doi: 10.1136/bmjopen-2021-050919
