# Supplementary material for: The Optimising Cardiac Surgery ouTcOmes in People with diabeteS (OCTOPuS) randomised controlled trial to evaluate an outpatient pre-cardiac surgery diabetes management intervention: a study protocol
Source: BMJ Open. 2021 Jun 9;11(6):e050919. doi: 10.1136/bmjopen-2021-050919 (PMC8191627; doi:10.1136/bmjopen-2021-050919)
Supplement: Supplementary data [file bmjopen-2021-050919supp003.pdf]

SAFETY CONSIDERATIONS

Definitions

Adverse Event (AE): any untoward medical occurrence in a participant or clinical study participant which does not necessarily have a causal relationship with study treatment or participation. An AE can therefore be any unfavourable and unintended sign (including an abnormal laboratory finding), symptom, or disease temporally associated with the study treatment or participation (regardless of causality assessments).

Serious Adverse Event (SAE) is any untoward medical occurrence or effect that:

- Results in death
- Is life-threatening, i.e. the participant was at risk of death at the time of the event
- Requires hospitalisation (regardless of length of stay), or prolongation of existing hospitalisation (>30 days post- cardiothoracic surgery)
- Results in persistent or significant disability or incapacity
- Is a congenital anomaly or birth defect
- Other important medical events (if they jeopardise the participant or require an intervention to prevent one of the above consequences).

It is the responsibility of the PI or delegate to grade an event as ‘not serious’ (AE) or ‘serious’ (SAE).

Seriousness

A complete assessment of the seriousness must always be assessed by a medically qualified doctor who is registered on the delegation of responsibility log; this is usually the investigator. All SAEs must be reported immediately by the PI at the participating centre to the SCTU.

Causality & Expectedness

A complete assessment of the causality must always be assessed by a medically qualified doctor who is registered on the delegation of responsibility log; this is usually the investigator. The nature or severity should be considered when making the assessment of expectedness. If these factors are not consistent with the current information available then the AE should be recorded as ’unexpected’.

Reporting Procedures

All adverse events should be reported until the End of Study as defined in 3.3. SAEs should be reported to SCTU within 24 hours of site becoming aware of the event. Additional information should be provided as soon as possible if the event has not resolved at the time of reporting. The reporting requirement for all AEs and SAEs affecting participants applies for all events occurring up to 90 days following cardiac surgery.

All unresolved adverse events should be followed by the investigator until resolved, the participant is lost to follow-up, or the adverse event is otherwise explained. At the last scheduled visit, the investigator should instruct each participant to report any subsequent event(s) that the participant, or the participant's general practitioner, believes might reasonably be related to participation in this study. The investigator should notify the study sponsor of any death or adverse event occurring at any time after a participant has discontinued or terminated study participation that may reasonably be related to this study.

Medically significant pre-existing conditions (those which are present prior to informed consent) should not be reported as an AE unless the conditions worsens during the trial. The condition, however, must be reported on the Medical History eCRF. Any adverse events which occur after informed consent taken should be recorded on the AE eCRF as per safety reporting section.

All SAEs should be reported within 24 hours of the local site becoming aware of the event. The SAE Non-CTIMP Form asks for nature of event, date of onset, severity, corrective therapies given, outcome, causality (i.e. unrelated, unlikely, possible, probably, definitely) and expectedness. The responsible investigator should assign the causality and expectedness of the event with reference to the events listed in Section 6.4.1.
