# Supplementary material for: The Optimising Cardiac Surgery ouTcOmes in People with diabeteS (OCTOPuS) randomised controlled trial to evaluate an outpatient pre-cardiac surgery diabetes management intervention: a study protocol
Source: BMJ Open. 2021 Jun 9;11(6):e050919. doi: 10.1136/bmjopen-2021-050919 (PMC8191627; doi:10.1136/bmjopen-2021-050919)
Supplement: Author's manuscript [file bmjopen-2021-050919.draft_revisions.pdf]

BMJ Open

BMJ Open is committed to open peer review. As part of this commitment we make the peer review history of every article we publish publicly available.

When an article is published we post the peer reviewers' comments and the authors' responses online. We also post the versions of the paper that were used during peer review. These are the versions that the peer review comments apply to.

The versions of the paper that follow are the versions that were submitted during the peer review process. They are not the versions of record or the final published versions. They should not be cited or distributed as the published version of this manuscript.

BMJ Open is an open access journal and the full, final, typeset and author-corrected version of record of the manuscript is available on our site with no access controls, subscription charges or pay-per-view fees (http://bmjopen.bmj.com).

If you have any questions on BMJ Open's open peer review process please email info.bmjopen@bmj.com

BMJ Open

Evaluation of a pre-cardiac surgery diabetes management intervention: a randomised-controlled trial

Journal:	*BMJ Open*
Manuscript ID	bmjopen-2021-050919
Article Type:	Protocol
Date Submitted by the Author:	04-Mar-2021
Complete List of Authors:	Holt, Richard; University of Southampton, Human Development and Health Academic Unit; University Hospital Southampton NHS Foundation Trust, Southampton National Institute for Health Research Biomedical Research Centre<br>Dritsakis, Giorgos; University of Southampton, Clinical Trials Unit<br>Barnard-Kelly, Katharine; Barnard Health – Health Psychology Research<br>Thorne, Kerensa; University of Southampton, Clinical Trial Units<br>Whitehead, Amy<br>Cohen, Lauren; Barnard Health<br>Dixon, Elizabeth; University of Southampton, Clinical Trial Units<br>Patel, Mayank; University Hospital Southampton, Department of Diabetes and Endocrinology<br>Newland-Jones, Philip; University Hospital Southampton, Department of Diabetes and Endocrinology<br>Green, Mark; University Hospital Southampton<br>Partridge, Helen; Royal Bournemouth and Christchurch Hospitals NHS Foundation Trust, Diabetes and Endocrinology<br>Luthra, Suvitesh; University Hospital Southampton, Division of Cardiac Surgery, Wessex Cardiothoracic Centre<br>Ohri, Sunil; University Hospital Southampton, Division of Cardiac Surgery, Wessex Cardiothoracic Centre<br>Salhiyyah, Kareem; Middle East University<br>Lord, Joanne; University of Southampton, Southampton Health Technology Assessments Centre<br>Niven, John; Patient Representative<br>Cook, Andrew; University of Southampton, Wessex Institute
Keywords:	Cardiothoracic surgery < SURGERY, DIABETES & ENDOCRINOLOGY, SURGERY

SCHOLARONE™
Manuscripts

I, the Submitting Author has the right to grant and does grant on behalf of all authors of the Work (as defined in the below author licence), an exclusive licence and/or a non-exclusive licence for contributions from authors who are: i) UK Crown employees; ii) where BMJ has agreed a CC-BY licence shall apply, and/or iii) in accordance with the terms applicable for US Federal Government officers or employees acting as part of their official duties; on a worldwide, perpetual, irrevocable, royalty-free basis to BMJ Publishing Group Ltd ("BMJ") its licensees and where the relevant Journal is co-owned by BMJ to the co-owners of the Journal, to publish the Work in this journal and any other BMJ products and to exploit all rights, as set out in our licence.

The Submitting Author accepts and understands that any supply made under these terms is made by BMJ to the Submitting Author unless you are acting as an employee on behalf of your employer or a postgraduate student of an affiliated institution which is paying any applicable article publishing charge ("APC") for Open Access articles. Where the Submitting Author wishes to make the Work available on an Open Access basis (and intends to pay the relevant APC), the terms of reuse of such Open Access shall be governed by a Creative Commons licence – details of these licences and which Creative Commons licence will apply to this Work are set out in our licence referred to above.

Other than as permitted in any relevant BMJ Author's Self Archiving Policies, I confirm this Work has not been accepted for publication elsewhere, is not being considered for publication elsewhere and does not duplicate material already published. I confirm all authors consent to publication of this Work and authorise the granting of this licence.

For peer review only

Evaluation of a pre-cardiac surgery diabetes management intervention: a randomised-controlled trial

Richard IG Holt[1-2], Giorgos Dritsakis[3], Katharine Barnard-Kelly[4,5], Kerensa I. Thorne[3], Amy Whitehead[3], Lauren Cohen[4], Elizabeth Dixon[3], Mayank Patel[6], Philip Newland-Jones[6], Mark Green[6], Helen Partridge[7], Suvitesh Luthra[6], Sunil Ohri[6], Kareem Salhiyyah[8], Jo Lord[9], John Niven[10], Andrew Cook[3,6] on behalf of the OCTOPuS study group

[1] Human Development and Health, Faculty of Medicine, University of Southampton, Southampton, UK

[2] Southampton National Institute for Health Research Biomedical Research Centre, University Hospital Southampton NHS Foundation Trust, Southampton, UK

[3] Clinical Trials Unit, Faculty of Medicine, University of Southampton, Southampton, UK

[4] Barnard Health – Health Psychology Research, Fareham, UK

[5] Faculty of Health & Social Science, Bournemouth University

[6] University Hospital Southampton, Southampton, UK

[7] Royal Bournemouth and Christchurch Hospitals NHS Foundation Trust, Bournemouth, UK

[8] Middle East University, Amman, Jordan

[9] Southampton Health Technology Assessments Centre, University of Southampton, UK

[10] Patient and public representative

Name and email address of corresponding author: Professor Richard Holt

R.I.G.Holt@soton.ac.uk

For peer review only - http://bmjopen.bmj.com/site/about/guidelines.xhtml

Abstract

Introduction: Cardiothoracic surgical outcomes are poorer in people with diabetes compared with those without diabetes. There are two important uncertainties in the management of people with diabetes undergoing major surgery; 1) how to improve diabetes management in the weeks leading up to an elective procedure, and 2) whether that improved management leads to better post-operative outcomes. We previously demonstrated the feasibility of delivering the OCTOPuS intervention, an outpatient intervention delivered by diabetes healthcare professionals for people with sub-optimally managed diabetes over 8-12 weeks before elective cardiac surgery. The present study will assess the clinical and cost-effectiveness of the intervention in cardiothoracic centres across the UK.

Methods and Analysis: A multicentre, parallel group, single-blinded 1:1 individually randomised trial comparing time from surgery until clinically fit for discharge in adults with sub-optimally managed type 1 or type 2 diabetes undergoing elective surgery between the OCTOPuS intervention and usual care. The target sample size will be 426 recruited across approximately 15 sites, with a pre-planned futility analysis after the first 100 participants have had their surgery based on an observed change in pre-operative HbA_{1c}. The primary analysis will be conducted on an intention to treat population. A two-sided p-value of 0.05 or less will be used to declare statistical significance for all analyses and results will be presented with 95% confidence intervals.

Ethics and dissemination. The trial was approved by the South Central - Hampshire A Research Ethics Committee (20/SC/0271). Results will be disseminated through conferences, scientific journals, newsletters, magazines and social media.

Trial Registration: ISRCTN10170306

Keywords: diabetes, cardiothoracic surgery, OCTOPuS intervention, randomised-clinical trial, post-operative outcomes

Word count: 3683

For peer review only - http://bmjopen.bmj.com/site/about/guidelines.xhtml

Article summary

Strengths and limitations of the study

- The OCTOPuS intervention was developed according to the MRC Framework for complex interventions and successfully piloted in a single cardiothoracic surgical centre.
- This is the first trial to assess whether early contact with a specialist diabetes team in the weeks leading up to surgery improves cardiothoracic surgical outcomes and reduces the excess morbidity and mortality experienced by people with diabetes.
- Hospital length of stay is an important clinical and economic measure of the success of surgery
- The sample size and number of sites will mean that the results are sufficiently generalisable to the remaining cardiothoracic centres across the UK
- The start of the study will likely be delayed by Covid-19 because of the effect of the epidemic on elective surgery.

For peer review only - http://bmjopen.bmj.com/site/about/guidelines.xhtml

1 INTRODUCTION

The prevalence of cardiovascular disease is increased approximately 2-fold in people with diabetes after adjustment for other cardiovascular risk factors.[1] It affects approximately a third of all people with type 2 diabetes and contributes to over 50% of deaths.[2] As coronary heart disease in people with diabetes tends to be more diffuse affecting multiple vessels, coronary artery bypass grafting is often the preferred method for re-vascularisation. Approximately 30-40% of all people undergoing open cardiac surgery have diabetes.[3]

Surgical outcomes are worse in people with diabetes with an up to three-fold higher risk of post-operative complications which include poor healing, wound complications, and renal dysfunction.[4,5] These complications are associated with longer hospital stay and higher re-admission rates. The reasons underlying the poorer outcomes include hyperglycaemia, dyslipidaemia and obesity. Although national and international groups have published detailed guidelines to improve surgical outcomes in people with diabetes, many people with diabetes are poorly prepared for surgery.[6-8] In the E-CABG study, 54% of people with type 2 diabetes treated with non-insulin medications and 67% of those with insulin-treated diabetes had an HbA_{1c} above 53 mmol/mol (7.0%) prior to cardiac surgery.[5]

There are two important uncertainties in the management of people with sub-optimally managed diabetes undergoing major surgery; 1) how to improve diabetes management in the weeks leading to elective surgery, and 2) whether that improved management is reflected in better surgical outcomes. To address these gaps, the overarching aim of the Optimising Cardiac Surgery ouTcOmes in People with diabeteS (OCTOPuS) project is to develop and test whether a pre-operative out-patient intervention to improve diabetes management improves cardiac surgical outcomes.

The development of the intervention is described in detail elsewhere (Holt et al. *under review*). In summary, the prototype OCTOPuS intervention was based on a nurse-led outpatient intervention that has been used in Royal Bournemouth Hospital for 7 years and incorporated the findings of two rapid literature reviews. A feasibility study conducted in 17 people with diabetes undergoing cardiothoracic surgery at University Hospital Southampton showed that it is possible to develop a clinical pathway to deliver the OCTOPuS intervention to improve glycaemic management prior to admission that was acceptable for people with diabetes and clinicians.

For peer review only - http://bmjopen.bmj.com/site/about/guidelines.xhtml

The present study will be a multicentre randomised controlled trial (RCT) in cardiothoracic centres across the UK to assess the clinical and cost-effectiveness of the intervention.

2 STUDY DESIGN

OCTOPuS is a multicentre, parallel group, single blind, individually randomised RCT incorporating a pre-planned futility analysis. It will compare time from surgery until an individual is clinically fit for discharge in adults with sub-optimally managed type 1 diabetes or type 2 diabetes undergoing elective cardiothoracic surgery between the OCTOPuS intervention and usual care.

3 ELIGIBILITY

3.1 INCLUSION CRITERIA

1. Aged ≥ 18 years old with type 1 diabetes or type 2 diabetes
2. Sub-optimally managed diabetes defined as an HbA_{1c} >53 mmol/mol (7%) for those ≤75 years old and an HbA_{1c} >64 mmol/mol (8%) for those >75 years old. The higher HbA_{1c} criterion for older people is to minimise the risk of iatrogenic hypoglycaemia. This will be measured using a near patient test at the cardiothoracic surgery outpatient appointment where the decision to proceed to surgery is made.
3. Awaiting elective open-heart cardiac surgery
4. Anticipated delay before surgery of at least 2 months.
5. Surgery will take place at a hospital participating in the trial
6. Ability to give informed consent.
7. Ability to interact with the study documentation and processes.

3.2 EXCLUSION CRITERIA

1. Active malignancy, where the malignancy is currently being treated by chemotherapy, surgery or radiotherapy or is likely to cause death within 6 months
2. Pregnancy
3. Previous cardiac surgery
4. Known haemoglobinopathies that affect the measurement of HbA_{1c}

For peer review only - http://bmjopen.bmj.com/site/about/guidelines.xhtml
For peer review only

5. Other illnesses or conditions that would preclude engagement with the OCTOPuS intervention
6. Surgery taking place outside the participating hospitals, e.g. at a private hospital

4 RECRUITMENT

4.1 SCREENING & CONSENT

Outpatient cardiac surgery appointment clinic lists will be scrutinised ahead of appointments and an information sheet explaining the trial will be sent by post or email as appropriate to people who appear eligible (including contact details to opt out if the person does not want further contact about the trial). Before the outpatient appointment, a researcher will contact the prospective participant to discuss the study at least 24 hours before the appointment allowing time for reflection and discussion. This will permit eligible individuals to be randomised immediately after the outpatient appointment, and where possible receive their OCTOPuS consultation, on the same day. The treating surgeon will remind eligible patients about the trial if a decision to proceed to surgery is made. If the person wishes to participate, they will have the opportunity to discuss the study face-to-face with a research nurse before they give written consent. Final eligibility criteria will be checked prior to recruitment. Patients whose medical records cannot be accessed prior to the appointment to determine eligibility (e.g. patients from another hospital), will be given information about the study on the day of the appointment and will be offered the opportunity to attend another day to discuss participation.

4.2 RANDOMISATION

Participants will be individually randomised in a 1:1 ratio, stratified by centre and age (≤75 years old and >75 years old), using permuted blocks. The study flow is illustrated in Figure 1.

[Figure 1 here]

For peer review only - http://bmjopen.bmj.com/site/about/guidelines.xhtml

5 STUDY PROCEDURES

5.1 BASELINE MEASUREMENTS

After randomisation, the following data will be collected on participants in both arms: medical history and examination; vital signs; biochemistry; self-reported episodes of hypoglycaemia

5.2 THE OCTOPUS INTERVENTION

5.2.1 Initial consultation

Participants randomised to receive the OCTOPuS intervention will have an initial consultation with an OCTOPuS practitioner, who may be a doctor, nurse, pharmacist, or other appropriately trained healthcare professional. In this consultation the participant's diabetes management will be discussed, as well as the likely benefits of improved glycaemic management prior to surgery. The practitioner and participant will agree actions, tailored to the individual needs and ability, including:

- A graded exercise regimen. This may be completely self-delivered, or alternatively by joining a local appropriate exercise scheme – such as a 'health walk'.
- Dietary advice, supplemented by a consultation with a dietitian if needed
- Medication review, which may lead to the introduction of insulin or other diabetes medications for people with type 2 diabetes.
- Specific advice about managing expectations, understanding facilitators to achieve change and overcoming barriers to improve medical and psychosocial outcomes

The exact process and the treatment options are set out in the OCTOPuS intervention manual (Supplementary Material).

5.2.2 Support calls

After the initial consultation, participants will receive regular review with the OCTOPuS practitioner, usually by telephone, at least once a fortnight until the participant's diabetes

For peer review only - http://bmjopen.bmj.com/site/about/guidelines.xhtml

management goals have been reached and no further changes are needed. After this, the frequency of the calls can be reduced at the discretion of the OCTOPuS practitioner and participant to a minimum of every 6 weeks. This contact will be an opportunity to offer encouragement and support and address any issues which have arisen for the participant. One more support contact will be made 1-3 weeks after discharge to ensure the continuity of diabetes management beyond surgery. Where necessary the OCTOPuS practitioner will liaise with local services, e.g. the participant's GP or a dietitian, to facilitate delivery.

5.3 CONTROL ARM

Participants in the control arm will receive usual care in the cardiac surgery centre attended by the individual. This is likely to contain standardised brief advice from the surgeon to pay attention to their diabetes prior to surgery. Some people may act on this advice, either on their own or in conjunction with their GP. The study will document 'usual care' at all recruiting centres and explore with participants in the control arm as part of the qualitative work what actions were taken in response to advice received.

5.4 FOLLOW UP VISITS

After participants are randomised to either the intervention or control arm, data will be collected from them at the following timepoints: pre-surgery; discharge; 7 days post-surgery; 30 days post-surgery; and at their next routine diabetes care visit between 90 and 180 days post-surgery. In addition to the baseline measures, information about the surgery, infections and surgical complications, mortality and adverse events will be collected (***TABLES Table* 1**). Pre-surgery, surgery and discharge data will be collected in hospital. After discharge, data will be collected remotely, e.g. over the phone, by post, through in-patient note review or where possible using adult cardiac surgery databases (e.g. the SCTS National Adult Cardiac Surgery Audit).

[Table 1 here]

For peer review only - http://bmjopen.bmj.com/site/about/guidelines.xhtml
For peer review only

6 ENDPOINTS

6.1 PRIMARY ENDPOINT

Time from surgery until clinically fit for discharge, as judged by the surgical team. Teams will be blinded to pre-hospital diabetes management allocation. This primary outcome was chosen because reduced time in hospital (though not at the expense of safety) is valued by people with diabetes, clinicians, and commissioners.

6.2 SECONDARY ENDPOINTS

- Time from surgery to actual discharge from hospital – this recognises that discharge can be delayed for non-clinical reasons
- Days alive between surgery and either out of hospital or judged as clinically fit for discharge
- Pre-operative mortality; 30 day mortality; 90 day mortality
- Time on ITU
- Time on a ventilator
- Sternal Wound Infections, defined according to the NICE guidance and the CDC criteria[10,11]
- Leg wound infections, in those who provide donor veins; graded according to the Centers for Disease Control and Prevention definitions of surgical site infections.[12]
- Chest infections, defined as a change in typical chest symptoms (cough, increase respiratory rate, shortness of breath) in conjunction with a fever or inflammatory markers.
- Urinary tract infections, defined as "clinically-diagnosed and treated, whether or not results from a urine culture are available''
- Acute Coronary Syndrome[12]
- Change in weight between randomisation and surgery
- Effect on post-operative renal function and incidence of acute kidney injury as assessed by measurement of serum creatinine and calculation of estimated glomerular filtration rates[12]
- HbA_{1c} immediately preoperative, and at between 90 and 180 days post operation.

For peer review only - http://bmjopen.bmj.com/site/about/guidelines.xhtml

- Change in HbA_{1c} between baseline and immediately preoperative, and change from preoperative to between 90 and 180 days post operation
- Operations cancelled for sub-optimal glycaemic management
- Frequency and severity of self-reported overall, minor, severe and nocturnal hypoglycaemia assessed at Baseline, during the Support Contact and Pre-surgery.[13]
- EQ-5D at baseline, 7, 30 and 90 days post-surgery.
- Qualitative interviews and psychosocial questionnaires at baseline and 90 days post-surgery to explore participants' experiences and perceived benefits of the intervention and any changes to their diabetes self-management.
- Cost effectiveness of intervention, including: use of NHS lifestyle improvement programs and diabetes services; use of medication, time spent by practitioners for training, delivering the intervention and liaising with local services; HbA_{1c} point-of-care and blood glucose monitoring costs

7 SAMPLE SIZE

7.1 FUTILITY ASSESSMENT – PHYSIOLOGICAL EFFECT OF INTERVENTION

To demonstrate that a physiological response is plausible we need to show an HbA_{1c} reduction of 5 mmol/mol in the intervention group pre-surgery compared to baseline. Previous experience shows the mean initial HbA_{1c} in our study population is approximately 72 mmol/mol, with a standard deviation of 15 mmol/mol.[14,15] For an expected change in HbA_{1c} from baseline of 5 mmol/mol in the intervention group, and assuming a correlation of 50% between baseline and pre-surgery, a sample size in the intervention group of 50 participants would allow a margin of error of 4.16 below the mean for a 95% confidence interval (CI) and would, therefore, allow us to exclude a difference of zero if the treatment difference of 5 was observed.

7.2 INTERVENTION EFFECTIVENESS – CLINICAL OUTCOMES

The primary outcome is the time from surgery to when the responsible consultant considers the participant clinically fit for discharge. We will not consider the actual discharge date in

For peer review only - http://bmjopen.bmj.com/site/about/guidelines.xhtml

the primary analysis, as currently many elective cardiothoracic surgical patients remain in hospital longer than clinically indicated due to their social situation. Discussions with clinicians and commissioners suggest that a mean improvement of half a day would be clinically worthwhile.

The current mean duration post-surgery until clinically fit for discharge is 7 days, with a standard deviation of 1.5 days. To demonstrate an improvement of 0.5 days with 90% power and 5% significance with 1:1 randomisation between intervention and control arms would require a total of 382 participants (nQuery v7.0). We will allow for a 5% loss to follow-up, and 5% for deaths post randomisation inflating the final target sample size to 426 participants. Participants will be recruited across approximately 15 UK cardiothoracic centres.

8 INTERIM ANALYSIS

Futility will be assessed, and the trial could be stopped early for one of two main reasons:

8.1 RECRUITMENT AND DELIVERY

There are several threats to recruitment and delivery of this trial:

- Being unable to recruit and initiate sufficient centres.
- Centres being unable to recruit sufficient participants
- Centres being unable to deliver the OCTOPuS intervention

Therefore throughout the trial, we will review progress against criteria at three time points, grading trial progress as red, amber, or green each time (Supplementary material).

For peer review only - http://bmjopen.bmj.com/site/about/guidelines.xhtml

8.2 PHYSIOLOGICAL EFFECT OF INTERVENTION

It is believed that the OCTOPuS intervention will have its clinically relevant effects through improvement of clinical measures, including change in body weight, exercise, lipid profile and blood pressure. However, the main target of the intervention is to improve glycaemic management; if no physiological effect can be demonstrated on glycaemic measures, continuation of the trial would be considered futile. After the first 100 participants have had their surgery, we will assess the effect of the intervention on pre-operative HbA_{1c}. If there is no discernible effect (defined as a change of HbA_{1c} of <5 mmol/mol) we will ask the trial steering committee to review the trial's viability.

9 STATISTICAL ANALYSIS

Baseline participant demographics and characteristics will be summarised between the two arms (16). The primary analysis will be conducted using ANCOVA adjusted for randomisation stratification factors on an intention to treat population. Continuous data will be presented as means and standard deviations and analysed using ANCOVA (or presented as medians and ranges and analysed using Mann-Whitney U tests if data are skewed). Binary data will be reported in terms of odds ratios and analysed using logistic regression modelling. Analysis of time-to-event outcomes will include presenting Kaplan-Meier graphs by arm and analysed using Cox proportional hazards regression (or competing risk regression as discussed below). A two-sided p-value of 0.05 or less will be used to declare statistical significance for all analyses and results will be presented with 95% confidence intervals. Subgroups will be investigated, including those with HbA_{1c} above or below 69 mmol/mol at presentation; type of diabetes; age above or below 75 years. The cut-off of 69 mmol/mol has been chosen as the level above which the Joint British Diabetes Societies recommend specific action to improve pre-operative glycaemic management. The cut-off for age has been chosen to reflect the different HbA_{1c} entry criteria for those above and below 75 years.

It is possible that a small proportion of participants will receive the intervention/usual care but will not actually undergo the planned surgery due to death, or clinically directed surgery cancellation. A small proportion may also undergo urgent revascularisation due to myocardial infarction after they have received their allocated treatment. A further group may undergo

For peer review only - http://bmjopen.bmj.com/site/about/guidelines.xhtml

surgery but die before they are fit for discharge and thus not meet the primary endpoint. It is expected that these events will occur in fewer than 5% of participants. These individuals will be excluded from the primary analysis but the prevalence of each of these outcomes will be monitored and recorded by treatment arm separately to assess if there is an excess of any of these outcomes in either group. A sensitivity analysis will be considered, looking at a competing risks model, where these outcomes and functional recovery are competing risks. This sensitivity analysis will also be performed if the total prevalence of these events exceeds 5%.

10 ECONOMIC EVALUATION

Quality adjusted life years (QALYs) will be estimated from EQ-5D-5L and mortality data, using the area-under-the-curve method. Similarly, costs will be estimated at the patient level. Mean between-group differences in QALYs and costs will be estimated using a regression-based approach, including adjustment for baseline covariates and interaction terms for pre-defined sub-groups, and allowing for clustering at hospital and/or practitioner level. Results will be presented as an Incremental Cost-Effectiveness Ratio (ICER) if appropriate. Non-parametric bootstrapping will be used to estimate confidence intervals around estimated cost differences and ICERs.

A simple modelling approach will also be used to estimate the costs and health impacts of surgical complications over a lifetime horizon. This extrapolation is necessary to reflect any mortality or lasting quality of life decrement associated with surgical complications. There will be no attempt to estimate the long-term impact of improved diabetes management related to the intervention, as it will be difficult to predict the duration over which any improvements will be maintained. This is likely to be a conservative assumption that will under-estimate the QALY gain and cost-effectiveness of intervention if it proves effective. Model parameters will be estimated from the trial and from other published sources. Long-term resource use, mortality and utility decrements associated with key surgical complications, will be identified by systematic review of HTAs, NICE guidelines and published literature.

For peer review only - http://bmjopen.bmj.com/site/about/guidelines.xhtml
For peer review only

11 QUALITATIVE & PSYCHOSOCIAL EVALUATION

11.1.1 Interviews

Fifty participants receiving the intervention will be recruited across all participating sites balanced for age, gender, HbA_{1c}, socioeconomic status and ethnicity. Baseline interviews will take place within 2 weeks of participants' commencing the intervention and follow-up interviews will be conducted with the same participants at 90 days post-surgery. Key personnel involved in the delivery will be interviewed once around 12 months after the start of trial in their centre.

Interview data analysis will include: (a) comparisons between participants' baseline and follow-up interviews to identify changes in their perceptions, experiences and diabetes self-management practices over time, and the reasons for these; (b) comparison of participant and health professional accounts to identify similarities and differences in their understandings and any impact on diabetes self-management practices; (c) cross-comparison of participants' accounts to identify common issues and experiences as well differences in diabetes self-management practices between subgroups of participants (e.g. men versus women, participants of different ages etc.), and the reasons for these.

11.1.2 Psychosocial Questionnaires

The following questionnaires will be completed by participants at baseline and at 3 months post-surgery:

- Diabetes Empowerment Scale (short form): an 8-item questionnaire assessing diabetes-related psychosocial self-efficacy.
- PAID5: a 5-item self-reported measure of diabetes related distress with high internal consistency.
- Patient Health Questionnaire (PHQ-2): ultra-brief depression screener, variant of PHQ-9. It is not used to establish a final diagnosis or to monitor depression severity but rather to screen for depression as a 'first step' approach.

For peer review only

For peer review only - http://bmjopen.bmj.com/site/about/guidelines.xhtml

- Brief Illness Perception Questionnaire (B-IPQ): an 8-item measure assessing cognitive illness representations, emotional representations, illness comprehensibility and perceived causal factors for illness.
- Summary of Diabetes Self-Care Activities scale (SDSCA): a 15-item self-report questionnaire of diabetes self-management that includes items assessing the following aspects of the diabetes regimen: general diet, specific diet, exercise, blood-glucose testing, foot care, and smoking.

The analysis of the questionnaire responses will aim to answer the following questions:

1. What effect does baseline score (categorised as high/low etc. as appropriate) have on study outcomes, i.e. days until considered fit for surgery?
2. What effect does the study intervention have on change in score assessed as a continuous variable from baseline to 90 days post-surgery?
3. Does the treatment work better or less well in people depending on their baseline score (categorised)?

12 SAFETY

Standard definitions and reporting procedures of adverse events, serious adverse events (SAEs), seriousness will be used (Supplementary material). For the purposes of this study, the following saes will not require reporting to Southampton Clinical Trials Unit:

- Hospitalisations for elective treatment of a pre-existing condition

Also, the following SAEs will not require reporting if they occur between 'Surgery' and 'Discharge':

- Arrhythmia, including atrial fibrillation
- Immediate postoperative surgical bleeding
- Pneumonia

Expectedness assessments are made against the list of expected events below:

- Minor musculoskeletal aches and pains
- Myocardial infarction
- Respiratory tract infection

For peer review only - http://bmjopen.bmj.com/site/about/guidelines.xhtml

13 PATIENT AND PUBLIC INVOLVEMENT

The trial has been developed in collaboration with the study patient and public involvement advisory group and local branch of Diabetes UK. The trial includes two patient representatives as a member of the Trial Steering Committee (TSC) and a member of the Trial Management group. Both individuals have been involved in the development of this protocol and have attended meetings regularly. To date, they have had an active role in assessing the study progress to date and both will be involved in resolving any issues that may arise.

14 ETHICS

Ethics approval was obtained by the South Central - Hampshire A Research Ethics Committee on 25 August 2020 (20/SC/0271). University Hospital Southampton NHS Foundation Trust will sponsor the study (RHM MED1718). The study is funded by the National Institute of Health Research *Health Technology Assessment* (*HTA*) Programme (16/25/12). The day-to-day management of the trial will be co-ordinated through the Southampton Clinical Trials Unit and oversight will be maintained by the Trial Steering Committee. The study will be conducted in accordance with WMA Declaration of Helsinki and as revised and recognised by governing laws and EU Directives.

All participants may withdraw at any time without providing a reason. Investigators will explain the value of remaining in study follow-up and allowing these data to be used for trial purposes. Where possible, those who have withdrawn from study treatment should remain in follow-up as per the trial schedule. If participants additionally withdraw consent for this, they will revert to standard clinical care. The study team will continue to collect standard follow-up data unless the participant explicitly states otherwise.

15 DISSEMINATION

Results will be disseminated through national and international conferences, scientific journals, newsletters, magazines and social media. Target audiences include diabetes

For peer review only

For peer review only - http://bmjopen.bmj.com/site/about/guidelines.xhtml

specialist teams, cardiac surgeons, primary care team and medical professionals or scientists overall as well as people with diabetes. This study addresses an important clinical question and is the first to assess whether early contact with a specialist diabetes team in the weeks leading up to surgery improves cardiothoracic surgical outcomes and reduces the excess morbidity and mortality experienced by people with diabetes. We further believe that the sample size and number of sites will mean that the results are sufficiently generalisable to broader cardiothoracic practice across the UK and internationally.

Author statement

RIGH is the chief investigator of the OCTOPuS study. He wrote the first draft of the protocol and will act as guarantor for the study. GD and ED will be the trial managers; GD further had significant contribution in editing and finalising the paper. KBK and LC wrote the qualitative sections of the protocol. KIT and AW wrote the statistical and data analysis sections of the protocol; AW was also involved in the original grant application and the design of the study. MP, PNJ and MG were involved in the design of the study. MP will lead the 'clinical' OCTOPuS intervention team and PNJ and MG will be members of the team. HP was involved in the design of the study and intervention. SL and SO were involved in the design of the study from the cardiothoracic perspective. JL wrote the health economics aspects of the protocol. JN is a patient representative on the trial management team and supported the development of the protocol. AC is associate director of the Clinical Trials Unit and was involved in the design of the study. KS was involved in the original grant application and the design of the study. All authors have critically revised the paper for intellectual content and approved the final draft. All authors agree to be accountable for all aspects of the work by ensuring that questions related to the accuracy or integrity of any part of the work are appropriately investigated and resolved.

Funding

The OCTOPuS project is funded by the UK National Institute for Health Research (NIHR) [Health Technology Assessment programme (grant number 16/25/12)]. The views expressed are those of the authors and not necessarily those of the NIHR or the Department of Health and Social Care.

For peer review only - http://bmjopen.bmj.com/site/about/guidelines.xhtml

Conflicts of interest

The authors have no competing interest to report.

Data statement

Participant data will be entered remotely at site and retained in accordance with current Data Protection Regulations. The PI will be responsible for ensuring the accuracy, completeness, and timeliness of the data entered. The participant data are pseudo-anonymised and the site retains a participant identification code list which is only available to site staff. Trained personnel with specific roles assigned will be granted access to the electronic case report forms (eCRF).

SCTU operate a transparent data sharing request process for results that are available in the public domain. As a minimum, anonymous data will be available for request from three months after publication of an article, to researchers who provide a completed Data Sharing request form that describes a methodologically sound proposal, for the purpose of the approved proposal and if appropriate a signed Data Sharing Agreement. Data will be shared once all parties have signed relevant data sharing documentation.

For peer review only

For peer review only - http://bmjopen.bmj.com/site/about/guidelines.xhtml

16 REFERENCES

1. Rao Kondapally Seshasai S, Kaptoge S, Thompson A, et al. Diabetes mellitus, fasting glucose, and risk of cause-specific death. *N Engl J Med* 2011;364(9):829-41. doi: 10.1056/NEJMoa1008862 [published Online First: 2011/03/04]
2. Einarson TR, Acs A, Ludwig C, et al. Prevalence of cardiovascular disease in type 2 diabetes: a systematic literature review of scientific evidence from across the world in 2007-2017. *Cardiovasc Diabetol* 2018;17(1):83. doi: 10.1186/s12933-018-0728-6 [published Online First: 2018/06/10]
4. Raza S, Blackstone EH, Sabik JF, 3rd. The diabetes epidemic and its effect on cardiac surgery practice. *J Thorac Cardiovasc Surg* 2015;150(4):783-4. doi: 10.1016/j.jtcvs.2015.07.037 [published Online First: 2015/08/25]
5. Biancari F, Giordano S. Glycated Hemoglobin and the Risk of Sternal Wound Infection After Adult Cardiac Surgery: A Systematic Review and Meta-Analysis. *Semin Thorac Cardiovasc Surg* 2019;31(3):465-67. doi: 10.1053/j.semtcvs.2019.02.029 [published Online First: 2019/03/10]
6. Nicolini F, Santarpino G, Gatti G, et al. Utility of glycated hemoglobin screening in patients undergoing elective coronary artery surgery: Prospective, cohort study from the E-CABG registry. *Int J Surg* 2018;53:354-59. doi: 10.1016/j.ijsu.2018.04.021 [published Online First: 2018/04/18]
7. Dhatariya K, Levy N, Kilvert A, et al. NHS Diabetes guideline for the perioperative management of the adult patient with diabetes. *Diabet Med* 2012;29(4):420-33. doi: 10.1111/j.1464-5491.2012.03582.x [published Online First: 2012/02/01]
8. Lazar HL, McDonnell M, Chipkin SR, et al. The Society of Thoracic Surgeons practice guideline series: Blood glucose management during adult cardiac surgery. *Ann Thorac Surg* 2009;87(2):663-9. doi: 10.1016/j.athoracsur.2008.11.011 [published Online First: 2009/01/24]
9. O'Neill F, Carter E, Pink N, et al. Routine preoperative tests for elective surgery: summary of updated NICE guidance. *BMJ* 2016;354:i3292. doi: 10.1136/bmj.i3292 [published Online First: 2016/07/16]
10. Older Adults: Standards of Medical Care in Diabetes-2019. American Diabetes Association. Diabetes Care 2019 Jan; 42 (Supplement 1): S139-S147. https://doi.org/10.2337/dc19-S012

For peer review only - http://bmjopen.bmj.com/site/about/guidelines.xhtml

11. https://www.nice.org.uk/guidance/ng125/evidence/full-guideline-pdf-6727105694
12. Garner JS, Jarvis WR, Emori TG, Horan TC, Hughes JM. CDC definitions for nosocomial infections, 1988. Am J Infect Control. 1988;16(3):128-40.
13. Biancari, F., Ruggieri, V.G., Perrotti, A., Svenarud, P., Dalén, M., Onorati, F., Faggian, G., Santarpino, G., Maselli, D., Dominici, C. and Nardella, S., 2015. European multicenter study on coronary artery bypass grafting (E-CABG registry): study protocol for a prospective clinical registry and proposal of classification of postoperative complications. *Journal of cardiothoracic surgery*, *10*(1), p.90.
14. Chakrapani Balijepalli, Eric Druyts, Gaye Siliman, Michel Joffres, Kristian Thorlund and Edward J Mills 2017 Hypoglycemia: a review of definitions used in clinical trials evaluating antihyperglycemic drugs for diabetes. Clin Epidemiol, 9: 291–296.
15. Hirst JA, Stevens RJ, Farmer AJ. Changes in HbA_{1c} level over a 12-week follow-up in patients with type 2 diabetes following a medication change. Barengo NC, editor. PLoS ONE. Public Library of Science; 2014;9(3):e92458.
16. Cohen O, Dankner R, Chetrit A, Luxenburg O, Langenauer C, Shinfeld A, et al. Multidisciplinary intervention for control of diabetes in patients undergoing coronary artery bypass graft (CABG). Cardiovasc Surg. 2003 Jun;11(3):195–200.
17. Moher D, Hopewell S, Schulz KF, Montori V, Gøtzsche PC, Devereaux PJ, et al. CONSORT 2010 Explanation and Elaboration: updated guidelines for reporting parallel group randomised trials. 2010;340:c869.

For peer review only

For peer review only - http://bmjopen.bmj.com/site/about/guidelines.xhtml

17 TABLES

Table 1. Summary of data collection during the OCTOPuS study at various time-points

Time-point	Screening	Consent	Baseline	Intervention	Support calls (every 2-6 weeks post-op)	Pre-Surgery assessments	Surgery	Discharge	Surgery +7 days	Post-discharge support call	Surgery +30 days	Surgery +90 days	End of study
Notes review	X												
Informed Consent		X											
Eligibility evaluation (incl. pregnancy test where appropriate)	X	X	X										
Medical History (incl. smoking status, diabetes and current medications)			X			X							
Physical Exam (incl. height, weight and			X (height will only be recorded			X							

peer review only

For peer review only - http://bmjopen.bmj.com/site/about/guidelines.xhtml

Time-point	Screening	Consent	Baseline	Intervention	Support calls (every 2-6 weeks post-op)	Pre-Surgery assessments	Surgery	Discharge	Surgery +7 days	Post-discharge support call	Surgery +30 days	Surgery +90 days	End of study
waist circumference)			at Baseline)										
Vital Signs (incl. BP)			X			X							
Biochemistry (incl HbA_{1c}, blood glucose and renal function)			X			X		X (only serum creatinine and renal function to capture acute kidney failure)				X (between 90-180 days post-op)	
Hypoglycaemia			X		X	X							
Infections and surgical complications								x			x	X	
Mortality						x					x	x	x
Intervention				X									

For peer review only

For peer review only - http://bmjopen.bmj.com/site/about/guidelines.xhtml

Time-point	Screening	Consent	Baseline	Intervention	Support calls (every 2-6 weeks post-op)	Pre-Surgery assessments	Surgery	Discharge	Surgery +7 days	Post-discharge support call	Surgery +30 days	Surgery +90 days	End of study
Intervention support phone call (incl. review of diary card and components of intervention utilised)					X					x			
Practitioner time (cost-effectiveness)			X	X	X					X			
NHS resource use questions (cost-effectiveness)						X							
Surgery (inc. time on ventilator/ITU)							X						
Blinded assessment								X					

For peer review only

Time-point	Screening	Consent	Baseline	Intervention	Support calls (every 2-6 weeks post-op)	Pre-Surgery assessments	Surgery	Discharge	Surgery +7 days	Post-discharge support call	Surgery +30 days	Surgery +90 days	End of study
Adverse Events			X	X	X	X			X	X	X	X	X
EQ-5D 5L			X						X		X	X	
Participant qualitative interview			X									X	
Psychosocial questionnaires			X									X	

For peer review only

For peer review only - http://bmjopen.bmj.com/site/about/guidelines.xhtml

18 FIGURE LEGENDS

Figure 1. OCTOPuS study flowchart.

For peer review only

For peer review only - http://bmjopen.bmj.com/site/about/guidelines.xhtml

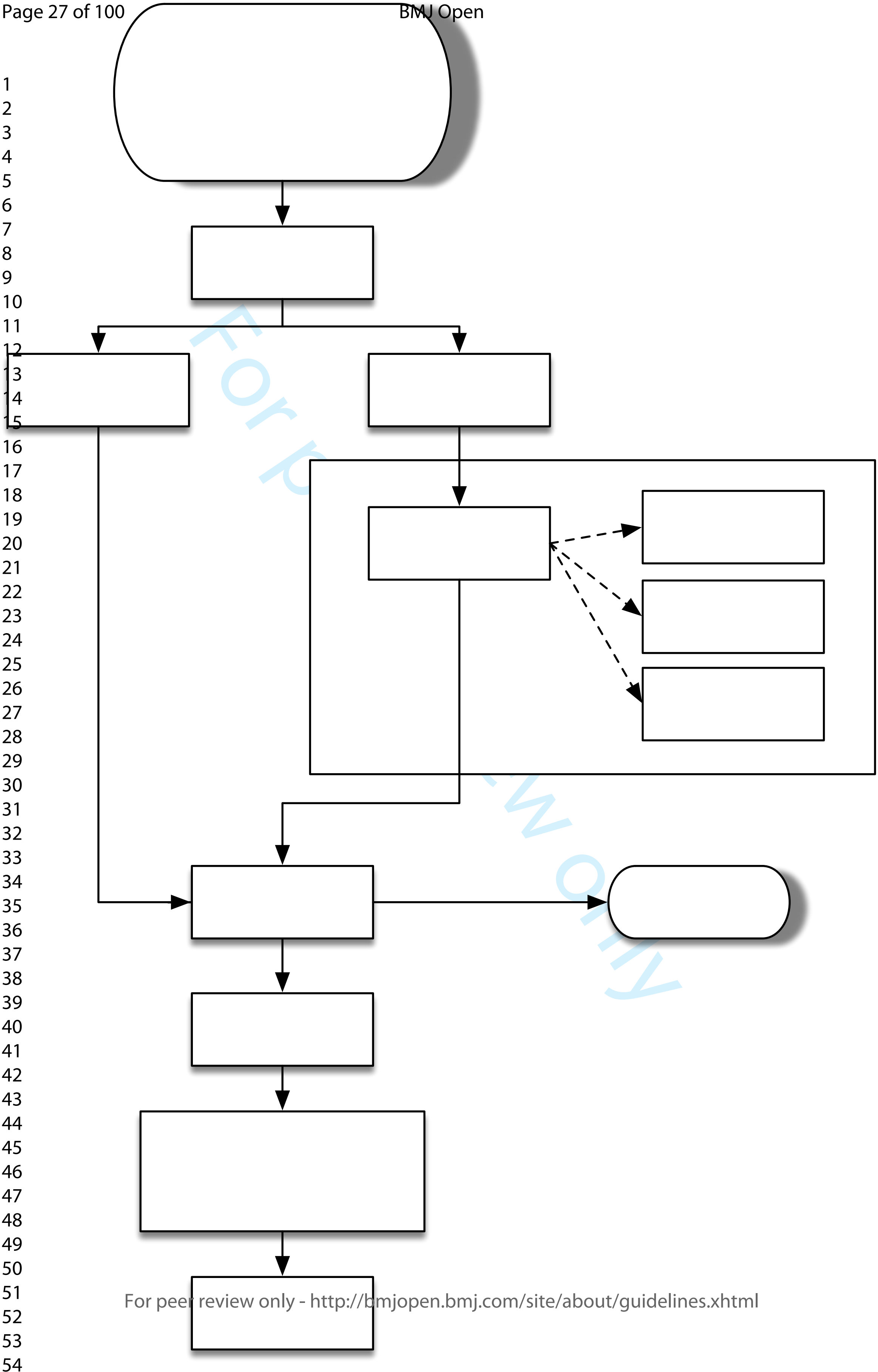

For peer review only - http://bmjopen.bmj.com/site/about/guidelines.xhtml

Southampton Clinical Trials Unit

OCTOPuS

Optimising Cardiac Surgery ouTcOmes in People with diabeteS

OCTOPUS Intervention Manual

Version Number 6
Date: 9-Jun-2020

IRAS reference number: 283351

For peer review only

For peer review only - http://bmjopen.bmj.com/site/about/guidelines.xhtml

OCTOPuS Intervention Manual

Welcome to the OCTOPuS Intervention Manual

The OCTOPuS intervention has been designed for people with diabetes who have been listed for cardiac surgery involving a sternotomy. The aim of the intervention is to improve diabetes control in the run up to surgery, with the goal of improving the surgical and post-surgical experience. Although the focus of the intervention is to improve glucose control, the intervention also includes the management of other aspects of diabetes, such as weight, that are known to affect surgical outcomes.

The intervention begins once an individual has been accepted for cardiac surgery and continues until the individual has had their cardiac surgery or the surgery is cancelled. Following discharge or if the surgery is cancelled, an individual's diabetes care will revert to their usual care prior to listing for surgery.

The OCTOPuS intervention will be tested in a randomised controlled trial, funded by the National Institute for Health Research Health Technology Assessment programme.

It is hoped that if the intervention is successful in improving surgical outcomes, it can be implemented in the National Health Service and adapted for other major surgery.

For peer review only

For peer review only - http://bmjopen.bmj.com/site/about/guidelines.xhtml

Change Control

Date	Version	Activity	Who
**19th Dec 2018**	2	Clarification on how many support calls are required.	Liz Dixon
**15th August 2019**	3	Clarification on timing of intervention and managing surgical queries.	Liz Dixon
**14th October 2019**	4	1. Additional support call post-surgery<br>2. Note regarding retinopathy	Giorgos Dritsakis, Richard Holt
**16th January 2020**	5	Changes regarding frequency of telephone calls and HbA1c measurement	Richard Holt
**9th June 2020**	6	Addition of remote initial consultation option	Richard Holt

For peer review only

For peer review only - http://bmjopen.bmj.com/site/about/guidelines.xhtml

Southampton Clinical Trials Unit

UNIVERSITY OF Southampton

Contents

1 ABOUT OCTOPUS AND THIS MANUAL 5

1.1 WHAT IS THE OCTOPUS INTERVENTION? 5
1.2 WHY IS THE OCTOPUS INTERVENTION NEEDED? 5
1.3 WHO IS THE OCTOPUS INTERVENTION FOR? 6
1.4 ABOUT THIS MANUAL 6

2 THE OCTOPUS INTERVENTION 7

2.1 THE OCTOPUS PRACTITIONER 7
2.2 GLUCOSE MANAGEMENT 7
2.2.1 Glucose monitoring 8
2.2.2 Lifestyle modification 9
2.2.3 Drug therapy for people with type 2 diabetes not currently receiving antidiabetes medication . 10
2.2.4 People with type 2 diabetes currently receiving oral antidiabetes medication 11
2.2.5 People with type 2 diabetes already on insulin 11
2.2.6 People with type 1 diabetes 11
2.2.7 Summary of antidiabetes medication options 12
2.2.8 Diabetes management during admission for cardiac surgery 14
2.3 LIPID MANAGEMENT 15
2.4 HYPERTENSION MANAGEMENT 15
2.5 WEIGHT MANAGEMENT 16
2.6 EXERCISE 16
2.7 SMOKING CESSATION 17
2.8 INVOLVEMENT OF SPOUSES, OR OTHER RELATIVES AND FRIENDS 17

3 DELIVERING THE OCTOPUS INTERVENTION 18

3.1 THE SCHEDULE OF EVENTS 18
3.1.1 The Initial Assessment 18
3.1.2 The Fortnightly Phone Call 20
3.1.3 Surgery and Beyond 21

APPENDIX 1: THE OCTOPUS RANDOMISED CONTROLLED TRIAL 22

APPENDIX 2: BIBLIOGRAPHY 23

Figures

Figure 1 - Diabetes medication flowchart for people with type 2 diabetes; based on the 2018 American Diabetes Association care standards 12
Figure 2 - A high-level flowchart of the OCTOPuS Intervention 18

Tables

Table 1 - Framework for Initial Assessment 19
Table 2 - Framework for Fortnightly Phone Call 20

For peer review only - http://bmjopen.bmj.com/site/about/guidelines.xhtml

1 About OCTOPuS and this manual

1.1 What is the OCTOPuS Intervention?

The OCTOPuS intervention is a set of elements, designed to improve the management of a person with diabetes over the weeks preceding scheduled major surgery. The components of the OCTOPuS intervention represent current best clinical practice and are endorsed by NICE or other guidelines. Suitably qualified and trained clinicians will deliver OCTOPuS.

The planned randomised evaluation (see Appendix 1) has been designed to assess the use of the OCTOPuS intervention for people with inadequately controlled diabetes undergoing cardiothoracic surgery, and this manual assumes that setting.

1.2 Why is the OCTOPuS intervention needed?

There are approximately 4 million people living with diagnosed and undiagnosed diabetes mellitus in the UK. Since 1996, the number of people diagnosed with diabetes has increased from 1.4 million to around 3.5 million. Diabetes increases the risk of cardiovascular disease by approximately two fold after adjustment for other cardiovascular risk factors. Ischaemic heart disease is by far the leading cause of death in people with diabetes accounting for approximately two-thirds of all deaths in those aged >65 years. Coronary heart disease tends to be more diffuse and progresses more rapidly in people with diabetes, which may explain why up to 35% of those presenting for elective cardiac revascularisation have diabetes.

The increasing number of people with diabetes will increase the demand for cardiac surgery in the future. These individuals have longer lengths of hospital stay and higher re-admission rates, placing a large financial burden on the NHS.

Poor glycaemic control increases the risk of wound and chest infections, renal impairment and death, especially following cardiac surgery. The Joint British Diabetes Societies for in-patient care has provided recommendations to improve the management of adults with diabetes undergoing surgery. As poor peri-operative glycaemic control is associated with an increased risk of all surgical complications, the guidelines recommend improving glycaemic control to optimise surgical outcomes.

Hyperglycaemia, however, does not wholly explain poorer surgical outcomes of people with diabetes; other important risk factors, such as obesity, hypertension and dyslipidaemia, are also more common in people with diabetes. Furthermore, lack of knowledge and training of the nursing and medical teams might also contribute.

If the pre-operative intervention is successful in improving glycaemic control and addressing other risk factors, this may reduce the complication rate and improve the clinical outcomes. It may also prove cost effective and even cost saving.

For peer review only - http://bmjopen.bmj.com/site/about/guidelines.xhtml

1.3 Who is the OCTOPuS intervention for?

The intervention can be offered to any person with sub-optimally controlled diabetes, which is defined as an HbA_{1c} >53 mmol/mol (>7%) using a point-of-care test at the cardiothoracic outpatient appointment where the decision to proceed to surgery is made.

The intervention described here assumes there is a period of at least 10 to 12 weeks before the scheduled surgery, but patients may derive benefit from a shorter intervention. In some circumstances, surgery may be delayed beyond 12 weeks. In this instance the pre-operative OCTOPuS intervention should continue until the patient is admitted surgery.

OCTOPuS is not suitable for people with malignancy, women who are pregnant or those with other illnesses or conditions that would preclude engagement with the intervention.

1.4 When should this manual be used?

The OCTOPuS intervention should begin as soon as possible after an individual has been accepted for cardiac surgery. The intervention continues until the individual has had their cardiac surgery or the surgery is cancelled. Following discharge or if the surgery is cancelled, an individual's diabetes care will revert to their usual care prior to listing for surgery.

1.5 About this manual

This manual describes the OCTOPuS intervention. At the time of writing, the intervention is still under development. The latest version of the manual can be obtained from the SCTU website (www.southampton.ac.uk/ctu) or from the OCTOPuS trial manager (octopus@soton.ac.uk).

This section has described the background and justification for the intervention.

Section 2 describes the components of the intervention, with a discussion of how each component might be delivered individually.

Section 3 discusses how these components are brought together and delivered as a coherent intervention.

For peer review only

For peer review only - http://bmjopen.bmj.com/site/about/guidelines.xhtml

2 The OCTOPuS Intervention

In this section, we describe the individual components of the intervention. How these components are tied together and delivered to the patient is described in section 3.

The OCTOPuS intervention comprises several elements, which are brought together in a systematic way. It is the role of the OCTOPuS practitioner to work with the patient awaiting surgery to decide which elements of the programme are applicable to the individual.

The recommendations in this manual represent the views of the OCTOPuS research team and are presented after careful consideration of the evidence and currently available NICE and international guidelines. When making treatment decisions with the participants, OCTOPuS practitioners are expected to take this manual into account, alongside the individual needs, preferences and values of their trial participants. It is not mandatory to apply the recommendations in the manual, and the guideline does not override the responsibility to make decisions appropriate to the circumstances of the individual, in consultation with them and their families and carers or guardian.

Given the short duration of the intervention and limited number of contacts, it may not be feasible to implement all actions and changes suggested by this manual. The OCTOPuS practitioner should work with the patient to implement as much as possible, planning to bring the patient to the best clinical status prior to surgery, but not delaying the planned surgical procedure in order to deliver OCTOPuS.

The sections below provide guidance in how to decide which elements might benefit each individual and guide the decision-making process. In general, the management below should follow NICE guidance for the management of diabetes, hypertension, dyslipidaemia and obesity unless specifically described otherwise.

2.1 The OCTOPuS practitioner

The OCTOPuS practitioner is a clinically qualified health care worker with expertise in diabetes. They are most likely to be a diabetes nurse specialist, but might, for example, be a pharmacist, dietitian or physician. The OCTOPuS practitioner will receive additional specific training about the OCTOPuS intervention.

Once a plan has been agreed, the practitioner supports the patient through regular contact (at least fortnightly until optimised), encouragement and counselling, signposting, and referral to key services.

Each practitioner will work with several patients awaiting surgery, providing initial advice, then remote telephone follow-up over the 3 months or so until the cardiac procedure. The OCTOPuS practitioner will need to provide advice about medication regimens, direct patients to local services, to advocate on patient's behalf, and to provide a listening ear to the patient.

2.2 Glucose Management

All patients eligible for the intervention will have an HbA_{1c} of >53mmol/mol (7.0%) and may benefit from improved glucose control. The challenge is to improve control without inducing episodes of hypoglycaemia, or excessive weight gain. The decision to intensify therapy should be made on an individual basis; for example, a more relaxed target may be appropriate for a

For peer review only - http://bmjopen.bmj.com/site/about/guidelines.xhtml

person with significant co-morbidities or where the risk and consequence of hypoglycaemia are high. There is a risk of worsening retinopathy in people with existing retinopathy if there is a rapid decline in HbA1c. The OCTOPuS practitioner should enquire whether the participant has a history of retinopathy and whether their retinal screening is up-to-date. The OCTOPuS practitioner should consider retinopathy when discussing treatment changes.

Improving glucose control may require lifestyle intervention, medication change or a combination of these strategies. How they can be delivered will be determined by local policies and funding. If the OCTOPuS practitioner is able to prescribe then they may undertake any initial changes themselves; otherwise, they should make recommendations to the patient's GP or local specialist diabetes team.

The patient's diabetes management and medication should be considered at every OCTOPuS interaction. The process is outlined in this section and summarised in Figure 1 on page 13. The OCTOPuS practitioner should provide the patient with a Diabetes UK Information Prescription about glycaemic management at each visit, if appropriate, to support glycaemic management.

2.2.1 Glucose monitoring

If the patient is not already monitoring their blood glucose, the OCTOPuS practitioner should provide the patient with a capillary glucose monitor and sufficient strips for the duration of the intervention (approximately 100 strips). They should teach the patient how to monitor and interpret their glucose using a standardised education package. All participants with Type 2 diabetes should be given a copy of 'Your Guide to Type 2 Diabetes' education booklet produced by Diabetes UK to keep for their personal reference and use throughout the study. Where applicable, participants should be shown online software management systems such as the Accu-Chek 360° Diabetes Management System. Although glucose monitoring is not usually recommended for routine use in people not using insulin or sulfonylureas, this is an important component of the intervention as short-term improvements in glucose control may not be apparent from changes in HbA_{1c} because of the short duration of the intervention.

In addition to any glucose monitoring that the patient is already undertaking prior to the intervention, patients should be advised to check their glucose levels 4 times a day (before meals and before bed) on the 3 days prior to the next OCTOPuS contact. The OCTOPuS practitioner should provide the patient with a glucose and diet diary so that the results can be recorded prior to the consultation. The OCTOPuS practitioner should encourage the patient to record what they eat in the diary so that any relationship between glucose readings and reported diet can be discussed at the consultation.

As most consultations will be remote, e.g. by telephone or Skype, where possible, the results should be sent to the OCTOPuS practitioner before the consultation, e.g. by email or Diasend (where available).

Following discussion between the OCTOPuS practitioner and patient, the glucose targets should be individualised, taking into account the risk of hypoglycaemia. However, typical glucose targets would be:

For peer review only - http://bmjopen.bmj.com/site/about/guidelines.xhtml

- Fasting and pre-meal glucose values: 4.0-6.0 mmol/L
- Post-meal and before bed glucose values: 4.0-7.8 mmol/L (for people using insulin who are prepared to test after meals)

2.2.2 Lifestyle modification

Dietary and exercise advice should follow the 2018 Diabetes UK nutritional guidelines (https://www.diabetes.org.uk/professionals/position-statements-reports/food-nutrition-lifestyle/evidence-based-nutrition-guidelines-for-the-prevention-and-management-of-diabetes). Where necessary, a healthcare professional with specific expertise and competencies in nutrition should see the patient.

Encourage high-fibre, low-glycaemic-index sources of carbohydrate in the diet, such as fruit, vegetables, wholegrains and pulses; include low-fat dairy products and oily fish; and control the intake of foods containing saturated and trans fatty acids. Encourage the patients to avoid excessive alcohol consumption.

The following leaflets may be of benefit:

British Heart Foundation:
Food labelling guidance: https://www.bhf.org.uk/publications/healthy-eating-and-drinking/this-label-could-change-your-life

Weight Loss advice:
https://www.bhf.org.uk/publications/healthy-eating-and-drinking/facts-not-fads---your-simple-guide-to-healthy-weight-loss

Nutrition and Diet Resource
Weight loss advice: describes 80kcal portions of different food groups so that the participants can decide how many of each they can have i.e. 1500kcal
https://www.ndr-uk.org/item/81/WeightManagement/Weight-Loss-You-Can-See-with-guidelines.html
This is a paid resource but could be useful for more visual patients

Carbs and cals – a variety of recipes, carb values, and low calorie meal options:
https://www.carbsandcals.com/weight-loss/weight-loss

British Dietetic Association:
Basic diet sheets for glycaemic index, healthy eating and weight loss
https://www.bda.uk.com/foodfacts/GIDiet.pdf
https://www.bda.uk.com/foodfacts/HealthyEating.pdf
https://www.bda.uk.com/foodfacts/Want2LoseWeight.pdf

For individuals who are overweight or obese, weight loss should be encouraged (see below).

For peer review only

For peer review only - http://bmjopen.bmj.com/site/about/guidelines.xhtml

The OCTOPuS practitioner should encourage the patient to become more physically active (see below).

2.2.3 Drug therapy for people with type 2 diabetes not currently receiving anti-diabetes medication

Almost all patients will need anti-diabetes drug therapy because of the elevated HbA_{1c} and short window-of-opportunity to improve the glucose control. The OCTOPuS practitioner should use their clinical judgement in the choice of medication, which should be individualised in discussion with the patient. All prescribing advice should be within the drug licence and take account of the individual Summary of Product Characteristics.

In line with NICE and 2018 American Diabetes Association guidance, the first line treatment of choice is standard release metformin, unless contraindicated. Metformin should be used according to its licence. Slow release metformin can be considered if the standard released metformin is not tolerated.

Where the initial HbA_{1c} is ≥75 mmol/mol (9%), consideration should be given to starting dual therapy from the outset as recommended by the 2018 American Diabetes Association guidance.

If metformin is insufficient to achieve adequate glycaemic control (as judged by capillary glucose monitoring described above), a second agent should be added. Because of the short-time scale of the intervention, it is not possible to base treatment changes on the measurement of HbA_{1c} for most patients. As all patients will have existing atherosclerotic cardiovascular disease, in line with the 2018 American Diabetes Association guidance, the first treatment intensification should usually be with a drug that has proven cardiovascular benefit, e.g. an SGLT-2 inhibitor (e.g. empagliflozin or canagliflozin) or a GLP-1 receptor agonist (e.g. liraglutide), unless contraindicated. The second treatment intensification should be the other class of drug (i.e. if an SGLT2 inhibitor was the first intensification then the second should be GLP-1 receptor agonist or vice versa), unless contraindicated. OCTOPuS practitioners will need to take account of drug-specific and patient factors as well as local formulary requirements.

The 2018 American Diabetes Association guidance diverges from current NICE guidance as the latter has not yet been updated in light of the latest cardiovascular outcome trials. However, there is a need to avoid weight gain and hypoglycaemia in this group of patients and there is a need to avoid provoking cardiovascular events as occurred in the ACCORD study.

Where these drugs are contraindicated, alternative agents, such as DPP-4 inhibitors, pioglitazone or sulfonylureas, e.g. gliclazide, can be used according to NICE guidance.

If adequate glycaemic control is not achieved with three non-insulin therapies, insulin should be initiated according to NICE guidance (see below). Where the initial HbA_{1c} is ≥86 mmol/mol

For peer review only - http://bmjopen.bmj.com/site/about/guidelines.xhtml

(10%), consideration should be given to starting insulin therapy from the outset as recommended by the 2018 American Diabetes Association guidance.

2.2.4 People with type 2 diabetes currently receiving oral anti-diabetes medication

Lifestyle factors, such as diet and physical activity, should be explored but it is likely that drug therapy will need to be intensified.

The OCTOPuS practitioner should explore whether the patient is taking the medication as prescribed. It is known that fewer than 50% of people receiving oral anti-diabetes treatments, antihypertensive agents and statins persist with their medication 2 years after treatment initiation and up to 20% never start treatment. The barriers to adherence should be discussed with the patient.

Where drug therapy intensification is needed, this should be done as described in the previous section 2.2.3. If the individual is on other combinations of oral anti-diabetes agents, the OCTOPuS practitioner should consider whether to change these to treatments with proven cardiovascular benefit.

2.2.4.1 Commencement of Insulin in people with type 2 diabetes

If adequate glycaemic control (judged by capillary glucose monitoring or presenting HbA_{1c}) is not achieved with three non-insulin therapies, insulin should be initiated according to NICE guidance. Basal insulin alone is the most convenient initial insulin regimen, beginning at 10 units per day or 0.1–0.2 units/kg/day, depending on the degree of hyperglycaemia.

As hypoglycaemia is a major risk factor for cardiovascular events, the OCTOPuS practitioner should have a low threshold to initiate insulin analogues instead of NPH insulin because of the lower risk of hypoglycaemia seen with the use of insulin analogues.

The OCTOPuS practitioner will need to liaise with local health services to ensure that the patient is offered sufficient training to use the insulin effectively. Given the urgency of treatment, in many instances, this will need to be outside usual channels.

Intensification of insulin is usually by the addition of prandial insulin or switch to pre-mixed insulin. The options should be discussed with the patient and a management plan agreed with the patient.

2.2.5 People with type 2 diabetes already on insulin

The OCTOPuS practitioner should review the current insulin regimen, injection technique and sites with the patient. The OCTOPuS practitioner should offer advice about the doses and types of injection as necessary.

2.2.6 People with type 1 diabetes

It is likely that the OCTOPuS practitioner will see people on a variety of insulin regimens, including both multiple daily injection and insulin pump therapy. The OCTOPuS practitioner

For peer review only - http://bmjopen.bmj.com/site/about/guidelines.xhtml

should review the current insulin regimen, injection technique and sites with the patient. The OCTOPuS practitioner should offer advice about the doses and types of injection as necessary.

If the patient has not attended a structured education course, this should be offered where possible.

A detailed description of insulin therapy is beyond the scope of this manual and the OCTOPuS practitioner should refer to the NICE type 1 diabetes guidance.

2.2.7 Summary of anti-diabetes medication options

The OCTOPuS practitioner should provide the patient with a Diabetes UK Information Prescription about glycaemic management at each visit to support glycaemic management.

Figure 1 summarises the options available to improve glycaemic control prior to surgery. The OCTOPuS practitioner should discuss progress with the patient at every visit or fortnightly phone call and management plan adjusted accordingly.

For peer review only

For peer review only - http://bmjopen.bmj.com/site/about/guidelines.xhtml

Southampton Clinical Trials Unit

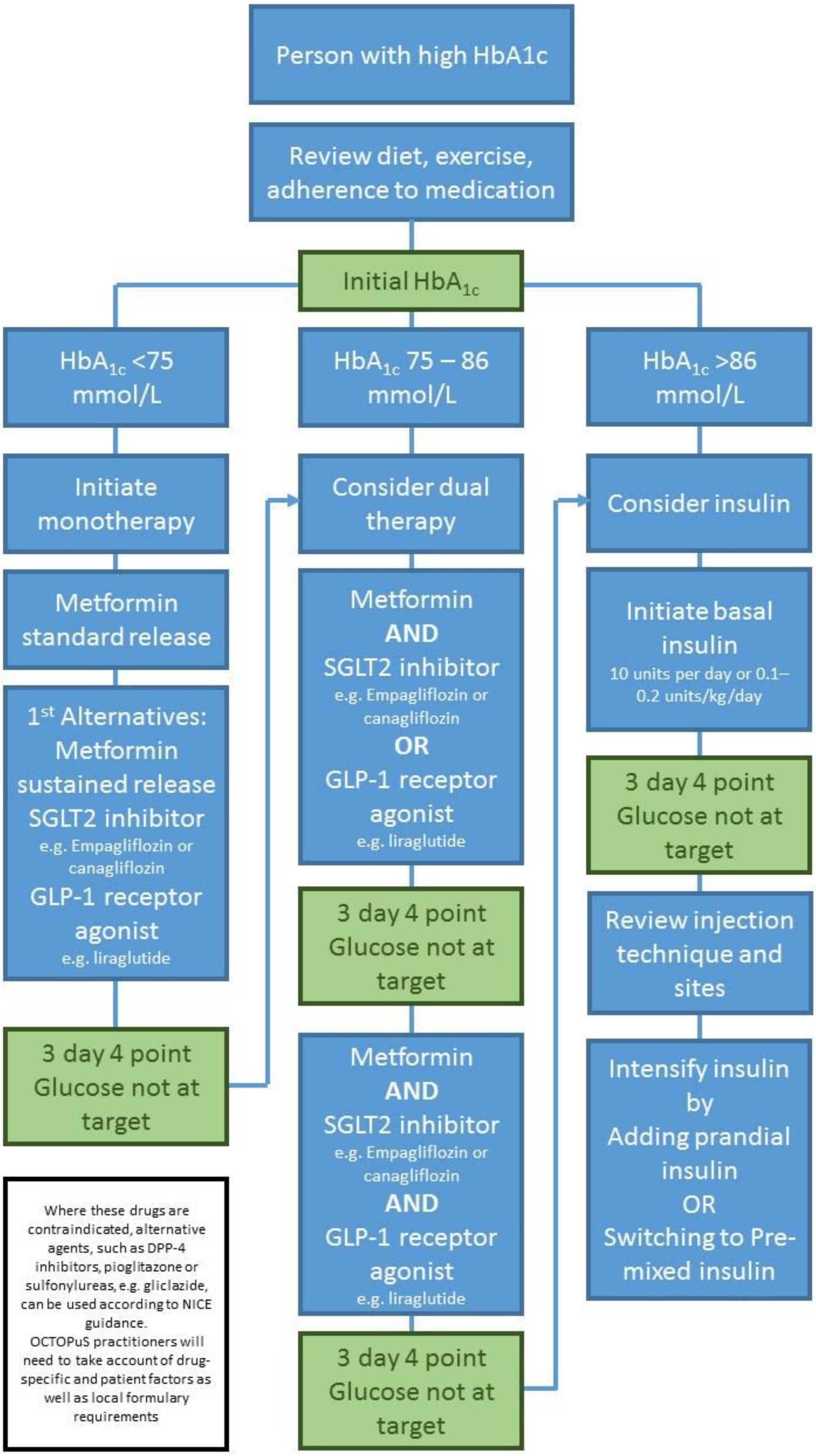

Figure 1 - Diabetes medication flowchart for people with type 2 diabetes; based on the 2018 American Diabetes Association care standards.

For peer review only - http://bmjopen.bmj.com/site/about/guidelines.xhtml

2.2.8 Diabetes management during admission for cardiac surgery

The OCTOPuS practitioner will need to provide advice about medication and glucose management when the patient is admitted for surgery. Where possible, the OCTOPuS practitioner should arrange to see the patient after admission but prior to surgery to provide advice about diabetes management during the admission.

The OCTOPuS practitioner may need to liaise with the diabetes in-patient team in the cardiothoracic centre, if not already a member of this team. Local protocols and JBDS guidance should be followed (http://www.diabetologists-abcd.org.uk/JBDS/Surgical_guidelines_2015_full_FINAL_amended_Mar_2016.pdf). There may be specific questions about cardiac surgery or management of diabetes during the operation that the OCTOPuS practitioner is unable to answer. If this is the case, the OCTOPuS practitioner should alert the cardiac surgeon so that the surgical team can answer these questions.

The OCTOPuS practitioner should advise the patient to continue to monitor their glucose, where appropriate. They should warn the patient of the risk of hypoglycaemia during fasting prior to admission. The OCTOPuS practitioner should advise the patient that oral anti-diabetes medications and GLP-1 receptor agonists need to be omitted on the day of surgery. The OCTOPuS practitioner should provide advice about adjustments to insulin doses. Suggested adjustments to insulin doses are as follows:

Insulin	Day before procedure	Day of procedure
Once daily (evening) (e.g. Lantus, Levemir, Tresiba, Abasalaglar Insulatard or Humulin I, Toujeo)	Take 80% of usual insulin dose at usual time.	Take 80% of usual insulin dose in the evening after the procedure
Once daily (morning) (e.g. Lantus, Levemir, Tresiba, Insulatard or Humulin I)	Take usual time.	Take 80% of usual insulin dose in the evening after the procedure
Twice daily (e.g. Novomix 30, Humulin M3, Humalog Mix 25 or 50, Lantus, Levemir)	Take usual time	Omit morning dose
Meal time injection	Take usual time	Omit all rapid insulin
Insulin pump	Please inform specialist pump team before admission for personalised advice. Continue with usual basal rates and continue to bolus depending on carbohydrate intake	Continue with usual basal rates and continue to bolus depending on carbohydrate intake

For peer review only - http://bmjopen.bmj.com/site/about/guidelines.xhtml

Following surgery but prior to discharge, the OCTOPuS practitioner should see the patient to discuss post-operative diabetes management. In some situations, it may be appropriate to discontinue insulin therapy. Any treatment changes and on-going management plans should be communicated to the patient's GP and specialist diabetes team, if necessary, following discharge after surgery. This information could be included in the discharge summary or communicated separately depending on local arrangements.

2.3 Lipid Management

Most patients will already be taking lipid-lowering therapy. However, if they are not taking a statin, the OCTOPuS practitioner should discuss the benefits of statin therapy and recommend that this is initiated if there are no contraindications or the person has previously not tolerated treatment with statins.

For those already taking lipid-lowering therapy, the OCTOPuS practitioner should review the patient's latest lipid profile. If a greater than 40% reduction in non-HDL cholesterol has not been achieved, the OCTOPuS practitioner should discuss adherence and timing of dose, optimise adherence to diet and lifestyle measures and consider increasing the statin dose if the participant is taking less than atorvastatin 80 mg. The OCTOPuS practitioner should consider the addition of ezetimibe. In some circumstances, these measures may be insufficient to control the lipid profile and in this situation, the OCTOPuS practitioner should considering recommending a referral to a specialist lipid clinic for consideration of PCSK9 inhibitors if this is available within the timeframe of the intervention.

Where changes have been recommended, the OCTOPuS practitioner should provide the patient with a Diabetes UK Information Prescription about lipid management.

2.4 Hypertension Management

Hypertension and endothelial dysfunction are common in people with diabetes. The OCTOPuS practitioner should measure the blood pressure or record the clinic blood pressure measurement as part of the initial assessment.

Preliminary data suggest that preoperative use of an antagonist of renin-angiotensin system (ACE inhibitor or angiotensin receptor blocker) in people undergoing CABG is associated with decreased in-hospital mortality. Unless contraindicated, the OCTOPuS practitioner should consider an antagonist of renin-angiotensin system (ACE inhibitor or angiotensin receptor blocker) for all patients after discussion with the local cardiothoracic surgical team. The dose should be titrated against the patient's blood pressure, which should be measured in the patient's general practice or by the patient at home. Further agents may be added in accordance with NICE guidance (CG127) as necessary. When an ACE inhibitor or angiotensin receptor blocker is added, the OCTOPuS practitioner should advise the measurement of urea and electrolytes and estimated glomerular filtration rate according to standard clinical

For peer review only - http://bmjopen.bmj.com/site/about/guidelines.xhtml

practice. At each contact, the OCTOPuS practitioner should advise the patients to withhold ACE inhibitors or angiotensin receptor blockers from 5 days prior to surgery.

Bear in mind that the surgical team may wish to commence a beta blocker prior to surgery, so if a second line agent is needed the OCTOPuS practitioner should consider commencing a beta blocker. This decision should be discussed with the local cardiothoracic surgical team.

Where changes have been recommended, the OCTOPuS practitioner should provide the patient with a Diabetes UK Information Prescription about blood pressure management.

2.5 Weight Management

Obesity and being overweight are associated with poor surgical outcomes. People with a BMI >25 Kg/m^2 are therefore likely to benefit from weight reduction, both to improve their diabetes control and surgical outcome.

The OCTOPuS practitioner should measure the patient's height and weight and calculate their BMI.

For recommendations on weight management, see the NICE guidelines on: preventing excess weight gain, weight management, and obesity. For most patients undertaking OCTOPuS, the major element of weight reduction will be through diet. Exercise is discussed in section 2.6 below.

The OCTOPuS practitioner should refer the patient or facilitate referral to a local NHS weight reduction programme or dietitian if this can be accessed quickly enough to achieve a worthwhile effect before surgery (e.g. the programme can be started within 4 weeks).

As described above, Diabetes UK and the British Heart Foundation both produce excellent leaflets about healthy eating and the OCTOPuS practitioner should provide the patient with a copy of these or let the patient know how to access them.

If an NHS option is not available, then other weight reduction options should be explored. This could include commercial programmes, such as Weight Watchers, or a self-managed diet.

2.6 Exercise

Physical activity has profound benefits for people with diabetes, including improved fitness, reduced insulin requirement and better glycaemic control, lower cardiovascular risk (lower blood pressure and improved lipid profile) and improved survival. People with diabetes should take at least 150 minutes of exercise per week spread over a minimum of 3 days with a mixture of aerobic exercise and resistance training.

However, care is needed in this group of patients as those awaiting cardiac revascularisation surgery may experience angina on exertion and have a limited capacity to provide oxygenated blood to cardiac muscle. Similarly, those awaiting valve surgery may not have significant capacity to exercise. Therefore, this component of the intervention will require the OCTOPuS practitioner to tailor any recommendation to the capacity of the individual.

For peer review only

For peer review only - http://bmjopen.bmj.com/site/about/guidelines.xhtml

It is also important to ascertain whether the individual has diabetes microvascular complications before starting to exercise. People with a history of active foot ulcers should avoid weight-bearing exercise and appropriate footwear should be worn.

However, where possible, the OCTOPuS practitioner should encourage patients to take gentle exercise, such as walking or dancing. The use of pedometers can support a gradual increase in physical activity. The best exercise is the one that the person enjoys. Practical advice should be given to help the person with diabetes find ways to become more physically active.

In the case of Type 1 diabetes, exercise can lead to unstable glucose levels during and immediately after exercise and a later risk of severe hypoglycaemia. Patients should be advised to avoid exercise of this intensity. As prior hypoglycaemia blunts the catecholamine response, people should be advised to avoid exercise within 24 hours of a severe hypoglycaemic episode and 1 hour of a self-treated episode. Exercise should also be avoided if blood ketone levels are increased. The OCTOPuS practitioner should consider whether a referral to a consultant diabetologist is required to address this complex area.

In type 2 diabetes, exercise does not usually cause hypoglycaemia and so carbohydrate supplementation is not required.

2.7 Smoking Cessation

All patients should be encouraged and supported to stop smoking, where possible. For recommendations on smoking cessation, see the NICE guidelines on: smoking: brief interventions and referrals, stop smoking services, and smoking: harm reduction.

2.8 Involvement of spouses, or other relatives and friends

The involvement of people important in the patient's life may lead to greater adherence to the components of the OCTOPuS intervention. Encouragement and support from friends and family will make the changes in medication, diet, and other activity, more sustainable over the few weeks that the intervention is delivered.

Therefore, if possible friends and relatives should be involved in the initial consultation, and consideration should be given to including them in the fortnightly phone calls.

For peer review only

For peer review only - http://bmjopen.bmj.com/site/about/guidelines.xhtml

3 Delivering the OCTOPuS Intervention

The elements described in section 2 are brought together in the OCTOPuS intervention.

3.1 The Schedule of Events

The intervention follows a series of events, shown in Figure 1. At each stage, the practitioner works with the patient to agree a set of goals and actions.

Figure 1 - A high-level flowchart of the OCTOPuS Intervention

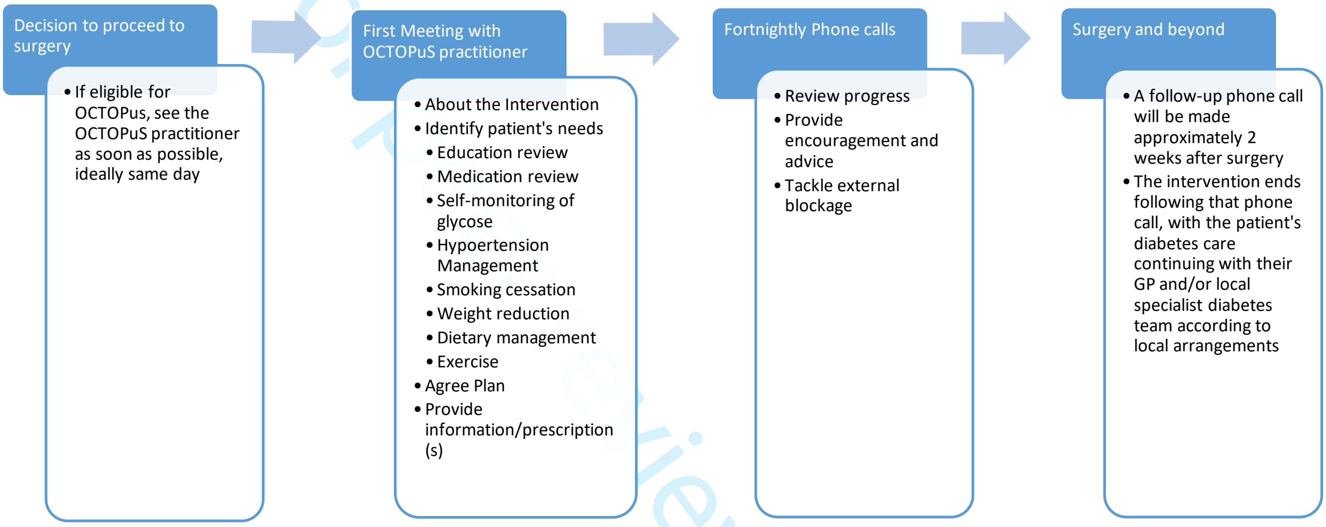

3.1.1 The Initial Assessment

The initial assessment is key to the OCTOPuS intervention. It is where the practitioner establishes a trust relationship, which will enable the patient to take the greatest advantage of the programme. We anticipate that the initial consultation will be usually be conducted face-to–face. However, alternative remote delivery options (video or telephone consultations) may be considered when these are necessary, for example, when the patient is unable to attend the hospital or to comply with COVID-19 social distancing.

For peer review only - http://bmjopen.bmj.com/site/about/guidelines.xhtml

Table 1 - Framework for Initial Assessment

Phase	Activity
**Assessment**	Explore the patient's understanding and experience of diabetes.<br>Explore any concerns the patient has relating to diabetes and their prospective surgery.<br>Establish whether there are relevant co-morbidities<br>Establish any significant people in the patient's life who could provide support in the run up to surgery.
**Explanation**	Explain the OCTOPuS intervention, and its goals, in the light of information elicited in the assessment phase.
**Measurement**	Make any clinical assessments which may be required, that weren't done in the most recent cardiovascular outpatient appointment<br>• HbA_{1c}<br>• Blood Pressure<br>• Height, Weight -> BMI
**Review**	Review the patient's situation in the areas set out in section 2 of this manual.<br>• Glucose Management<br>• Hypertension Management<br>• Weight Reduction<br>• Smoking Status<br>• Exercise<br>Agree a plan, where appropriate, with the patient for each of these elements
**Follow-up**	Schedule the first fortnightly phone call. You may like to schedule multiple calls for the complete period up to the planned surgery date.<br>Obtain permission from the patient to contact their GP or other services to support the patient's action plan. Liaise with local clinical team where necessary<br>Put into place any arrangements needed to support the patient's action plan

For peer review only - http://bmjopen.bmj.com/site/about/guidelines.xhtml

3.1.2 The Fortnightly Phone Call

At least every fortnight, the OCTOPuS practitioner will contact the patient until the patient's diabetes management is optimised or no further changes are possible. After this has been achieved, the frequency of the calls can be reduced at the discretion of the OCTOPuS practitioner to a minimum of every 6 weeks. We envisage this being by phone but other methods (face-to-face, Skype etc.) could be used by mutual agreement.

This is an opportunity for the practitioner to review progress against goals, and for the patient to raise any queries they might have.

The OCTOPuS practitioner should ensure that the patient has the contact details of the OCTOPuS team so that they can contact the team if necessary.

Table 2 - Framework for Fortnightly Phone Call

Phase	Activity
**Assessment**	Explore the patient's activity and progress against the goals agreed at the previous initial assessment or fortnightly phone call<br><br>Explore whether there has been any changes health, e.g. infection, that might affect the management plan.
**Measurement**	Make any clinical assessments, which may be required. This will generally involve the patients reporting over the phone or by alternative means of communication, such as email, DIASEND, Freestyle Libreview etc.<br>• Recent self-monitoring of blood glucose<br>• Weight<br>• Smoking status
**Listen**	To any problems or concerns that the patient raises

For peer review only

For peer review only - http://bmjopen.bmj.com/site/about/guidelines.xhtml

Phase	Activity
**Review**	Review the patient's situation in the areas set out in section 2 of this manual.<br>• Glucose Management – where surgery is delayed beyond 3 months, the OCTOPuS practitioner should arrange for a further HbA1c measurement every 3 months<br>• Hypertension Management<br>• Weight Reduction<br>• Smoking Status<br>• Exercise<br>Agree a plan, where appropriate, with the patient for each of these elements, in the light of the review and listening phases.<br>Liaise with local clinical team where necessary
**Follow up**	Schedule the next fortnightly phone call, if not already done.

3.1.3 Surgery and Beyond

Surgery will proceed according to local protocols.

Approximately two weeks after surgery, the OCTOPuS practitioner will contact the patient again. This phone call will follow the framework in Table 2 above. During the final support call, the OCTOPuS practitioner should undertake a review of the elements of the intervention and develop a future diabetes management plan.

All patients who have completed the OCTOPuS intervention and their surgical treatment should then return to routine diabetes care with their GP or local diabetes specialist team.

For peer review only

For peer review only - http://bmjopen.bmj.com/site/about/guidelines.xhtml

Appendix 1: The OCTOPuS Randomised Controlled Trial

The OCTOPuS trial has been funded by the NIHR HTA programme to evaluate the OCTOPuS intervention, and to determine whether it adds value to patient care.

Approximately 426 people with poorly controlled diabetes undergoing cardiac surgery will be randomised to either the OCTOPuS intervention or to usual care. The outcomes of interest include time from surgery until clinically for hospital discharge, 30 & 90 day mortality, wound infections, chest infections, renal impairment, HbA_{1c} pre-op and, 90 days post op, cost-effectiveness, procedures cancelled due to glycaemic control, quality of life, patient satisfaction & experience.

More information is available at https://www.journalslibrary.nihr.ac.uk/programmes/hta/162512/#/

For peer review only

For peer review only - http://bmjopen.bmj.com/site/about/guidelines.xhtml

Appendix 2: Bibliography

1 Zheng J, Cheng J, Wang T, *et al.* Does HbA1c Level Have Clinical Implications in Diabetic Patients Undergoing Coronary Artery Bypass Grafting? A Systematic Review and Meta-Analysis. *Int J Endocrinol* 2017;**2017**:1537213–8. doi:10.1155/2017/1537213

2 Rollins KE, Varadhan KK, Dhatariya K, *et al.* Systematic review of the impact of HbA1c on outcomes following surgery in patients with diabetes mellitus. *Clinical Nutrition* 2016;**35**:308–16. doi:10.1016/j.clnu.2015.03.007

3 Boreland L, Scott-Hudson M, Hetherington K, *et al.* The effectiveness of tight glycemic control on decreasing surgical site infections and readmission rates in adult patients with diabetes undergoing cardiac surgery: A systematic review. *Heart Lung* 2015;**44**:430–40. doi:10.1016/j.hrtlng.2015.06.004

4 Cheng X, Tong J, Hu Q, *et al.* Meta-analysis of the effects of preoperative renin–angiotensin system inhibitor therapy on major adverse cardiac events in patients undergoing cardiac surgery. *Eur J Cardiothorac Surg* 2015;**47**:958–66. doi:10.1093/ejcts/ezu330

5 NICE. *Hypertension in adults: diagnosis and management*. 2011.

6 NICE. *Preventing excess weight gain*. NICE 2015.

7 NICE. *Weight management: lifestyle services for overweight or obese adults*. NICE 2014.

8 NICE. *Obesity: identification, assessment and management*. NICE 2014.

For peer review only

For peer review only - http://bmjopen.bmj.com/site/about/guidelines.xhtml

PROGRESS GRADING

Actions to be taken depending on progress grade

Grade	Action
Green	Continue trial, keeping an eye on accrual.
Amber	Working with governance committees (TSC, TMG, PPI Committees), seek root cause for under performance. Consider whether these can be mitigated through work with organisations or individuals within the study.
Red	Review the study with governance committees, taking steps as detailed under amber, but also explicitly considering recommending study closure.

Progress grading time points and criteria

Assessment Point	Green	Amber	Red
After 100 patients have had surgery (50 intervention and 50 control)	HbA_{1c} reduction in intervention group >5mmol/mol	HbA_{1c} reduction in intervention group <5mmol/mol	HbA_{1c} reduction in intervention group not consistent with physiological effect

For peer review only - http://bmjopen.bmj.com/site/about/guidelines.xhtml

SAFETY CONSIDERATIONS

Definitions

Adverse Event (AE): any untoward medical occurrence in a participant or clinical study participant which does not necessarily have a causal relationship with study treatment or participation. An AE can therefore be any unfavourable and unintended sign (including an abnormal laboratory finding), symptom, or disease temporally associated with the study treatment or participation (regardless of causality assessments).

Serious Adverse Event (SAE) is any untoward medical occurrence or effect that:

- Results in death
- Is life-threatening, i.e. the participant was at risk of death at the time of the event
- Requires hospitalisation (regardless of length of stay), or prolongation of existing hospitalisation (>30 days post- cardiothoracic surgery)
- Results in persistent or significant disability or incapacity
- Is a congenital anomaly or birth defect
- Other important medical events (if they jeopardise the participant or require an intervention to prevent one of the above consequences).

It is the responsibility of the PI or delegate to grade an event as 'not serious' (AE) or 'serious' (SAE).

Seriousness

A complete assessment of the seriousness must always be assessed by a medically qualified doctor who is registered on the delegation of responsibility log; this is usually the investigator. All SAEs must be reported immediately by the PI at the participating centre to the SCTU.

Causality & Expectedness

A complete assessment of the causality must always be assessed by a medically qualified doctor who is registered on the delegation of responsibility log; this is usually the investigator. The nature or severity should be considered when making the assessment of expectedness. If these factors are not consistent with the current information available then the AE should be recorded as 'unexpected'.

For peer review only

Reporting Procedures

All adverse events should be reported until the End of Study as defined in 3.3. SAEs should be reported to SCTU within 24 hours of site becoming aware of the event. Additional information should be provided as soon as possible if the event has not resolved at the time of reporting. The reporting requirement for all AEs and SAEs affecting participants applies for all events occurring up to 90 days following cardiac surgery.

All unresolved adverse events should be followed by the investigator until resolved, the participant is lost to follow-up, or the adverse event is otherwise explained. At the last scheduled visit, the investigator should instruct each participant to report any subsequent event(s) that the participant, or the participant's general practitioner, believes might reasonably be related to participation in this study. The investigator should notify the study sponsor of any death or adverse event occurring at any time after a participant has discontinued or terminated study participation that may reasonably be related to this study.

Medically significant pre-existing conditions (those which are present prior to informed consent) should not be reported as an AE unless the conditions worsens during the trial. The condition, however, must be reported on the Medical History eCRF. Any adverse events which occur after informed consent taken should be recorded on the AE eCRF as per safety reporting section.

All SAEs should be reported within 24 hours of the local site becoming aware of the event. The SAE Non-CTIMP Form asks for nature of event, date of onset, severity, corrective therapies given, outcome, causality (i.e. unrelated, unlikely, possible, probably, definitely) and expectedness. The responsible investigator should assign the causality and expectedness of the event with reference to the events listed in Section 6.4.1.

For peer review only

University Hospital Southampton NHS Foundation Trust

UNIVERSITY OF Southampton

OCTOPuS

Optimising Cardiac Surgery ouTcOmes in People with diabeteS

For peer review only

Version 1 2 Jun 2020

SPONSOR: University Hospital Southampton NHS Foundation Trust
COORDINATING CENTRE: Southampton Clinical Trials Unit

ISRCTN reference:	ISRCTN10170306
Ethics reference number:	283351
Sponsor reference number:	RHM MED1718
Funder reference number:	16/25/12
ICD10	[Insert]

UKCRC Registered Clinical Trials Units

Protocol authorised by:

**Name:**	Professor Richard Holt	**Role:**	Chief Investigator
**Signature:**		**Date:**	
**Name:**	Professor Gareth Griffiths	**Role:**	Director of SCTU
**Signature:**		**Date:**	
**Name:**		**Role:**	On behalf of Sponsor
**Signature:**		**Date:**	

MAIN STUDY CONTACT

Chief Investigator and Medical Expert: Professor Richard Holt

The Institute of Developmental Sciences (IDS Building)
MP887 University of Southampton
Southampton General Hospital
Tremona Road
Southampton SO16 6YD UK

Tel: 023 8120 4665
Email: R.I.G.Holt@soton.ac.uk

STUDY COORDINATION CENTRE

For general study and clinical queries e.g. participant queries, study supplies, data collection, please contact in the first instance:

OCTOPuS Clinical Trial Manager(s): Elizabeth Dixon
Tel: 02381 203866
Email: OCTOPuS@soton.ac.uk

Address: Southampton Clinical Trials Unit
Southampton General Hospital
Tremona Road
SOUTHAMPTON
SO16 6YD

Tel: 023 8120 5154
Fax: 0844 774 0621
Email: ctu@soton.ac.uk
Web: www.southampton.ac.uk/ctu

SPONSOR

University Hospital Southampton NHS Foundation Trust is the research sponsor for this study. For further information regarding sponsorship conditions, please contact the Director or Research and Development at:

Address: R&D Department
University Hospital Southampton NHS Foundation Trust
SGH, Level E, Laboratory & Pathology Block, SCBR, MP 138
Tremona Road
SOUTHAMPTON
SO16 6YD

Tel: 023 8120 4989
Web: www.uhs.nhs.uk

CO-INVESTIGATOR(S)

Co-Investigators can be contacted via the Trial Coordination Centre.

Dr Andrew Cook	University of Southampton
Dr Mayank Patel	University Hospital Southampton NHS Foundation Trust
Dr Helen Partridge	Royal Bournemouth and Christchurch Hospitals NHS Foundation Trust
Mr Philip Newland- Jones	University Hospital Southampton NHS Foundation Trust
Mr Suvitesh Luthra	University Hospital Southampton NHS Foundation Trust
Mr Kareem Salhiyyah	Farah Medical Campus, Amman, Jordan
Professor Joanne Lord	University of Southampton
Professor Katharine Barnard-Kelly	Bournemouth University
Dr Amy Whitehead	University of Southampton
Ms Michelle Light	University of Southampton
Mr John Niven	PPI
Professor Sunil Ohri	University Hospital Southampton NHS Foundation Trust

For peer review only

For peer review only - http://bmjopen.bmj.com/site/about/guidelines.xhtml

FUNDER

This study is funded by the NIHR Health Technology Assessment programme, project number 16/25/12.

Protocol Information

This protocol describes the OCTOPuS study and provides information about procedures for entering participants. The protocol should not be used as a guide for the treatment of other non-study participants; every care was taken in its drafting, but corrections or amendments may be necessary. These will be circulated to investigators in the study, but sites entering participants for the first time are advised to contact Southampton Clinical Trials Unit to confirm they have the most recent version.

Compliance

This study will adhere to the principles of Good Clinical Practice (GCP). It will be conducted in compliance with the protocol, in accordance with current Data Protection Regulations and all other regulatory requirements, as appropriate.

For peer review only

SCHEDULE OF OBSERVATIONS AND PROCEDURES 10
1 INTRODUCTION 12
1.1 BACKGROUND 12
1.2 OCTOPUS INTERVENTION DEVELOPMENT 12
1.3 THE OCTOPUS SURVEY 14
2 RATIONALE & OBJECTIVES 14
3 STUDY DESIGN 15
3.1 PRIMARY ENDPOINT 15
3.2 SECONDARY ENDPOINTS 15
3.3 DEFINITION OF END OF STUDY 16
4 SELECTION AND ENROLMENT OF PARTICIPANTS 16
4.1 CONSENT 16
4.2 INCLUSION CRITERIA 17
4.3 EXCLUSION CRITERIA 17
4.4 SCREENING FAILURES 18
4.5 REGISTRATION/RANDOMISATION PROCEDURES 18
4.6 CONTRACEPTION 18
5 STUDY OBSERVATIONS AND PROCEDURES 18
5.1 SCREENING PROCEDURES 18
5.2 STUDY PROCEDURES 19
5.3 FOLLOW UP 20
5.4 DEVIATIONS AND SERIOUS BREACHES 20
5.5 STUDY DISCONTINUATION 20
5.6 WITHDRAWAL 21
5.7 PROHIBITED AND RESTRICTED THERAPIES DURING THE STUDY 21
5.8 BLINDING AND PROCEDURES FOR EMERGENCY UNBLINDING 21
6 SAFETY 21
6.1 DEFINITIONS 21
6.2 SERIOUSNESS 22
6.3 CAUSALITY 22
6.4 EXPECTEDNESS 23
6.5 REPORTING PROCEDURES 23
6.6 SCTU RESPONSIBILITIES FOR SAFETY REPORTING TO REC 25
7 STATISTICS AND DATA ANALYSES 25
7.1 METHOD OF RANDOMISATION 25
7.2 SAMPLE SIZE 25
7.3 INTERIM ANALYSIS 26
7.4 STATISTICAL ANALYSIS PLAN (SAP) 26

For peer review only

For peer review only - http://bmjopen.bmj.com/site/about/guidelines.xhtml

7.5 ECONOMIC EVALUATION 27
7.6 QUALITATIVE & PSYCHOSOCIAL OUTCOMES 28
8 REGULATORY 30
8.1 CLINICAL TRIAL AUTHORISATION 30
9 ETHICAL CONSIDERATIONS 30
9.1 SPECIFIC ETHICAL CONSIDERATIONS 31
9.2 ETHICAL APPROVAL 31
9.3 INFORMED CONSENT PROCESS 31
9.4 CONFIDENTIALITY 31
10 SPONSOR 32
10.1 INDEMNITY 32
10.2 FUNDING 32
10.3 AUDITS AND INSPECTIONS 32
11 STUDY OVERSIGHT GROUPS 32
11.1 TRIAL MANAGEMENT GROUP (TMG) 33
11.2 TRIAL STEERING COMMITTEE (TSC) 33
11.3 INDEPENDENT DATA MONITORING COMMITTEE (IDMC) / DATA MONITORING AND ETHICS COMMITTEE (DMEC) 33
12 DATA MANAGEMENT 33
12.1 DATA SHARING 34
13 MONITORING 35
13.1 CENTRAL MONITORING 35
13.2 CLINICAL SITE MONITORING 35
13.3 SOURCE DATA 35
14 RECORD RETENTION AND ARCHIVING 35
15 PUBLICATION POLICY 35
16 REFERENCES 37
17 APPENDICES 39
17.1 APPENDIX 1- PROGRESS GRADING 39
18 SUMMARY OF SIGNIFICANT CHANGES TO THE PROTOCOL 42

For peer review only

For peer review only - http://bmjopen.bmj.com/site/about/guidelines.xhtml

LIST OF ABBREVIATIONS

AE	Adverse Event
CRF	Case Report Form
CTCAE	Common Terminology Criteria for Adverse Events
DMEC	Data Monitoring and Ethics Committee
GCP	Good Clinical Practice
IDMC	Independent Data Monitoring Committee
IDSG	Intervention Development Steering Group
ISF	Investigator Site File
ITU	Intensive Treatment Unit
MHRA	Medicines and Healthcare products Regulatory Agency
NCI	National Cancer Institute
REC	Research Ethics Committee
SAE	Serious Adverse Event
SCTU	Southampton Clinical Trials Unit
TMF	Trial Master File
TMG	Trial Management Group
TSC	Trial Steering Committee

KEYWORDS
Diabetes, Intervention, Cardiac, Cardiothoracic, Surgery, Out-patient

For peer review only

For peer review only - http://bmjopen.bmj.com/site/about/guidelines.xhtml

STUDY SYNOPSIS

**Short title/Acronym:**	OCTOPuS
**Full title:**	Optimising Cardiac Surgery ouTcOmes in People with diabeteS

**Study Phase:**	III
**Population:**	Adults (≥18 yrs) with sub-optimally controlled type 1 or type 2 diabetes (HbA_{1c} >53 mmol/mol if aged 18-75 years or HbA_{1c} >64 mmol/mol if aged >75 years) undergoing elective cardiac surgery, who are clinically able to wait at least 2 months for their surgery.
**Primary Objective:**	To investigate whether an outpatient intervention, delivered in the weeks prior to elective major cardiac surgery, can improve outcomes for people whose diabetes is sub-optimally controlled.
**Secondary Objectives:**	• Through qualitative and psychosocial research, assess patients' and clinicians' experience of receiving and delivering the intervention, respectively.<br>• Health economic evaluation of the intervention
**Rationale:**	There are currently two important uncertainties in the management of people with sub-optimally controlled diabetes undergoing intermediate and major surgery;<br>• how to improve diabetes management in the weeks leading up to an elective procedure, and<br>• whether that improved management is reflected in improved outcomes post-surgery.<br>We have previously assessed the feasibility of the OCTOPuS intervention, an outpatient intervention delivered to people with sub-optimally controlled diabetes before elective cardiac surgery. The present study will assess whether this intervention can reduce HbA_{1c} levels and improve post-surgery clinical outcomes.
**Study Design:**	A multicentre, parallel group, single-masked, individually randomised trial incorporating a pre-planned futility analysis comparing time from surgery until clinically fit for discharge in adults with sub-optimally controlled type 1 or 2 diabetes undergoing elective surgery between the OCTOPuS intervention and usual care.
**Sample size :**	426
**Treatment/Intervention:**	An outpatient based intervention delivered over approximately 8-12 weeks prior to surgery

**URL for Database:**	https://www.imedidata.com

**Primary Study Endpoints:**	Time from surgery until clinically fit for discharge.
**Secondary Study Endpoints:**	• Actual time from surgery to discharge from hospital<br>• Days alive and either out of hospital or judged as clinically fit for discharge<br>• Pre-operative mortality<br>• 30 day mortality

For peer review only - http://bmjopen.bmj.com/site/about/guidelines.xhtml

	• 90 day mortality<br>• Time on ITU<br>• Time on a ventilator<br>• Sternal Infections<br>• Leg wound infections, in participants who provide donor vein<br>• Chest infections<br>• Urinary tract infections<br>• Acute myocardial infarction<br>• Change in weight between randomisation and surgery<br>• Effect on post-operative renal function and incidence of acute kidney injury<br>• HbA_{1c} immediately preoperative, and at between 90 and 180 days post operation.<br>• Change in HbA_{1c} between baseline and immediately preoperative, and change from preoperative to between 90 and 180 days post operation<br>• Frequency and severity of self-reported overall, minor, severe and nocturnal hypoglycaemia<br>• Operations permanently cancelled for sub-optimal glycaemic control<br>• EQ-5D at baseline, 7 days, 90 days and (if found feasible and effective) 30 days post-surgery.<br>• Cost effectiveness of intervention<br>Use of NHS lifestyle improvement programs and diabetes services<br>Use of medication<br>Time spent by practitioners for a) training, b) delivering the intervention and c) liaising with local services<br>HbA_{1c} point-of-care and blood glucose monitoring costs<br>• Psychosocial questionnaires at baseline and 90 days post surgery using:<br>Problem areas in diabetes (PAID)<br>Brief illness perception questionnaire (BIPQ)<br>Diabetes empowerment scale (DES5)<br>Summary of diabetes self-care activities (SDSCA)<br>Patient health questionnaire 2 (PHQ-2)
**Total Number of Sites:**	Approximately 15

For peer review only - http://bmjopen.bmj.com/site/about/guidelines.xhtml

STUDY SCHEMA

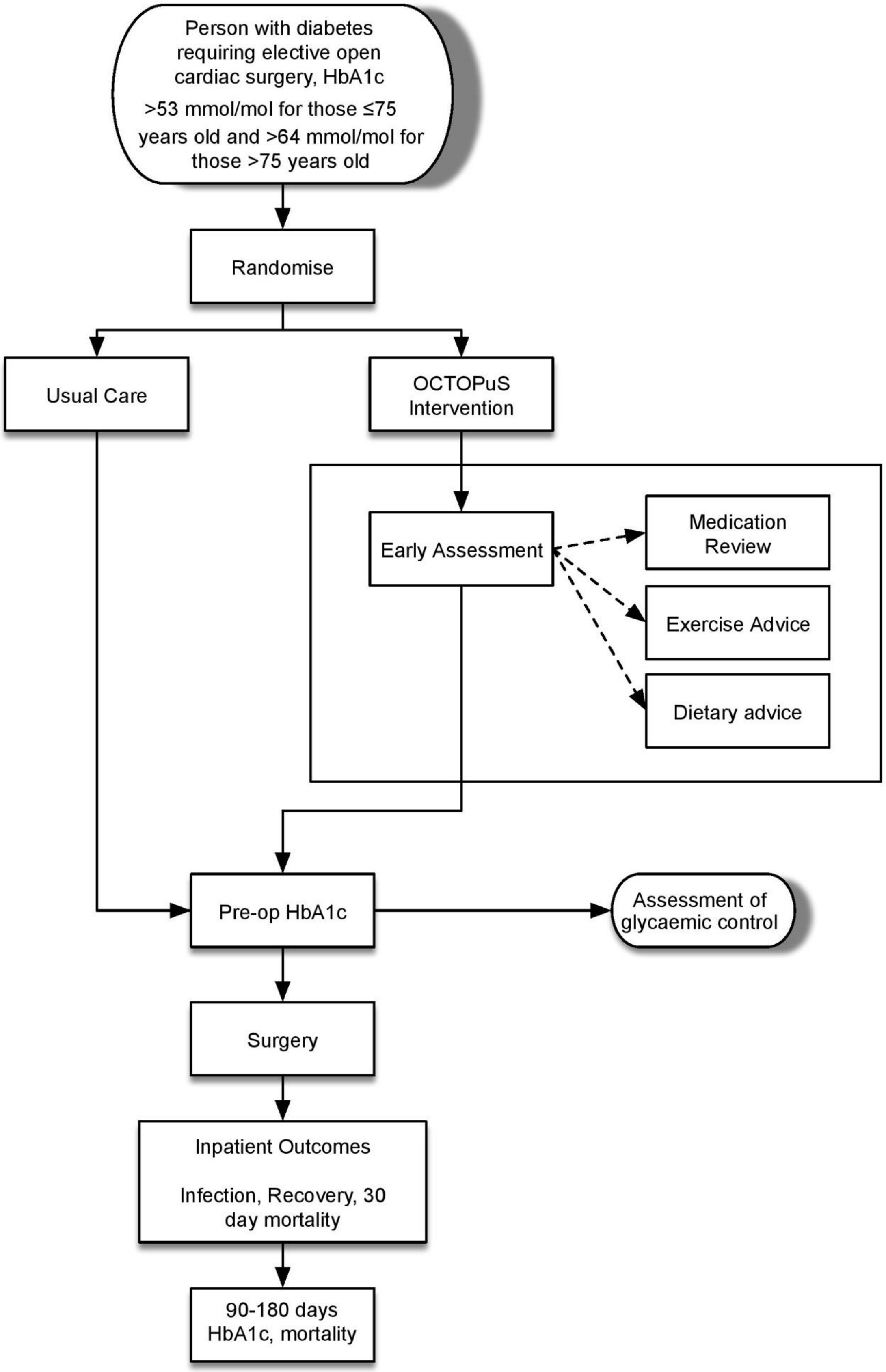

For peer review only - http://bmjopen.bmj.com/site/about/guidelines.xhtml

SCHEDULE OF OBSERVATIONS AND PROCEDURES

Visit:	Screening	Consent	Baseline	Intervention	Support calls[1]	Pre-Surgery assessments	Surgery	Discharge	Surgery +7 days	Post-discharge support call[2]	Surgery +30 days	Surgery +90 days	End of study
Notes review	X												
Informed Consent		X											
Eligibility evaluation (incl. pregnancy test where appropriate)	X	X	X										
Medical History (incl. smoking status, diabetes and current medications)			X			X							
Physical Exam (incl height, weight and waist circumference)			X[3]			X							
Vital Signs (incl. BP)			X			X							
Biochemistry (incl HbA_{1c}, blood glucose and renal function)			X			X		X[4]				X[5]	
Hypoglycaemia			X		X	X							
Infections and surgical complications								x			x	X	
Mortality						x					x	x	x
Intervention				X									
Intervention support phone call (incl. review of diary card and					X					x			

[1] Every 2 to 6 weeks until surgery.
[2] One to 3 weeks after discharge.
[3] Please note: 'Height' will only be recorded at 'Baseline'.
[4] Only serum creatinine and renal function will be measured to capture acute kidney failure.
[5] This can be completed between 90 and 180 days post-surgery.

For peer review only

For peer review only - http://bmjopen.bmj.com/site/about/guidelines.xhtml

Visit:	Screening	Consent	Baseline	Intervention	Support calls[1]	Pre-Surgery assessments	Surgery	Discharge	Surgery +7 days	Post-discharge support call[2]	Surgery +30 days	Surgery +90 days	End of study
components of intervention utilised)													
Practitioner time (cost-effectiveness)			X	X	X					X			
NHS resource use questions (cost-effectiveness)						X							
Surgery (inc. time on ventilator/ITU)							X						
Blinded assessment								X					
Adverse Events			X	X	X	X			X	X	X	X	X
EQ-5D 5L			X						X		x[6]	X	
Participant qualitative interview			X[7, 8]									X[7]	
Psychosocial questionnaires			X									X	

The Participant/legal representative is free to withdraw consent at any time without providing a reason. When withdrawn, the participant will continue to receive standard clinical care. Follow up data will continue to be collected (unless the participant/legal representative has specifically stated that they do not want this to happen).

Key health professionals involved in the delivery of the intervention will be interviewed once around 12 months after the start of trial in their centre but as the timing is independent from the study visits and will vary for each site, these interviews are not included in the above table.

[6] Only if feasible and effective (see section 7.5).
[7] For those randomised to intervention.
[8] Interviews will be conducted with 50 participants within 6 weeks of surgery.

For peer review only - http://bmjopen.bmj.com/site/about/guidelines.xhtml

For peer review only

1 INTRODUCTION

1.1 BACKGROUND

There are approximately 4 million people living with diagnosed and undiagnosed diabetes mellitus in the UK (1). Since 1996, the number of people diagnosed with diabetes has increased from 1.4 million. Diabetes increases the risk of cardiovascular disease by approximately 2-fold after adjustment for other cardiovascular risk factors. Ischaemic heart disease is by far the leading cause of death in people with diabetes accounting for approximately two thirds of all deaths in those aged >65 years. Coronary heart disease tends to be more diffuse and progresses more rapidly in people with diabetes which may explain why up to 35% of those presenting for elective cardiac revascularisation have diabetes (2). Sub-optimal glycaemic control increases the risk of wound and chest infections, renal impairment and death, especially following cardiac surgery (3-7).

The increasing number of people with diabetes will increase the demand for cardiac surgery in the future. These patients have longer lengths of hospital stay and higher re-admission rates, placing a large financial burden on the NHS. If the pre-operative intervention is successful in improving glycaemic control, this may reduce the complication rate and improve the clinical outcomes. It may also prove cost effective and even cost saving.

The Joint British Diabetes Societies for in-patient care provided recommendations to improve the management of adults with diabetes undergoing surgery (3). As sub-optimal peri-operative glycaemic control is associated with an increased risk of all surgical complications, the guidelines recommend improving glycaemic control to optimise surgical outcomes.

Since 2011, the diabetes team at the Royal Bournemouth Hospital have worked to optimise the surgical experience of people with diabetes. Using a nurse-led outpatient intervention, delivered around 3 months before surgery to people with sub-optimally controlled diabetes, they achieved a reduction in HbA_{1c} from 85 mmol/mol at first referral to 74 mmol/mol on admission for surgery. This has been associated with a reduced length of stay from a mean of 5.9 days to a mean of 3 days, while the length of stay for those without diabetes remained constant at 5 days. Other work has shown the practicality of improving HbA_{1c} over a period of weeks in primary care (8).

The OCTOPuS project was established to address whether a pre-operative out-patient healthcare professional delivered intervention to improve diabetes management improves cardiac surgical outcomes for people with diabetes. The project is divided into two parts:

1. Intervention Development
2. Randomised Controlled Trial to evaluate the effectiveness and cost effectiveness of the intervention developed in part 1

The Intervention Development part of the study was covered by a separate ethics application and protocol (18/SC/0508) and is summarised below. The rest of this protocol refers explicitly to the OCTOPuS Randomised Controlled Trial, hereafter referred to as the OCTOPuS study.

1.2 OCTOPUS INTERVENTION DEVELOPMENT

1.2.1 Feasibility study

1.2.1.1 Aims

A feasibility study was conducted to assess whether the intervention is acceptable for people with diabetes and clinicians and can be delivered in a multicentre randomised controlled trial.

For peer review only - http://bmjopen.bmj.com/site/about/guidelines.xhtml

More specifically we explored (1) whether any changes needed to be made to the first draft of the manual and (2) if there are any potential barriers in the process that could inform the main trial.

1.2.1.2 Methods

Participants were recruited through 5 cardiac surgery outpatient clinics at University Hospital Southampton NHS Foundation Trust since March 2019 using the same process anticipated for the main trial. In brief, clinic lists were screened several weeks prior to the clinic for potential participants. They were contacted by letter and follow-up phone call to assess their willingness to take part in the study. On the day of their clinic visit, if they were listed for cardiac surgery, they were recruited to the study if they fulfilled the inclusion/exclusion criteria. After providing informed written consent, the participants receive their first OCTOPuS consultation from a diabetes specialist healthcare professional (OCTOPuS practitioner) either on the day of their outpatient appointment with the surgeon or at a dedicated weekly OCTOPuS clinic established explicitly for the study. A formal diabetes management was agreed to help prepare the participant for surgery. Participants were then contacted by an OCTOPuS practitioner, initially at least fortnightly, to assess their diabetes management and to provide on-going encouragement and support to help the participant achieve their diabetes goals. The precise format of this contact was driven by the OCTOPuS intervention manual. Feedback from the participants experiencing the intervention and clinicians delivering the intervention was obtained using semi-structured interviews with the aim of refining and finalising the manual in preparation for the main trial.

1.2.1.3 Process evaluation

We evaluated the recruitment and intervention delivery process throughout the study to ensure the fidelity of the intervention when scaled up. The main barriers and measures we took to mitigate are presented below:

Challenge	Solution
Recruiting participants on the same day as their outpatient appointment	Dedicated OCTOPuS clinic every Thursday afternoon
Small number of potential candidates when recruiting from one cardiac surgery clinic	Expansion to all 5 UHS clinics
Large number of people >75 years old who were excluded only on the basis of age and could benefit from the intervention	Recruiting participants aged >75 years old. We used a higher HbA_{1c} threshold of >64 mmol/mol to reduce the risk of iatrogenic hypoglycaemia

1.2.1.4 Results

Overall, the feedback from both participants and clinicians was positive and both groups were more focused on pre-operative diabetes management after taking part in the OCTOPuS trial. Participants reported that taking part in the trial improved their health prior to surgery, and felt supported and satisfied with the care they had received. High levels of anxiety relating to the prospect of cardiothoracic surgery were reported by some participants. As the OCTOPuS intervention provided an opportunity to alleviate some of these fears, we added clarifications to the OCTOPuS manual regarding the impact of diabetes on cardiac surgery.

From the practitioners' point of view, participants seemed happy after surgery and were less likely to need referral to inpatient diabetes services after surgery. For them, the main challenge was how to fit the time-commitment to the study within NHS normal practice. Establishing the dedicated OCTOPuS clinic allowed much better time management and was easier for the OCTOPuS team.

For peer review only - http://bmjopen.bmj.com/site/about/guidelines.xhtml

Most of the OCTOPuS manual performed well and only minor changes to the manual were needed during the intervention development. However, further changes may be required once all the qualitative feedback has been analysed. Preliminary analysis demonstrated a reduction in HbA_{1c} between baseline and surgery but the sample size was too small to draw conclusions about the magnitude of this effect.

It is envisaged that the manual will be a dynamic document and will be updated as appropriate in response to changes in clinical practice during the main trial.

1.2.1.5 The OCTOPuS training package

A training package will be developed at the end of the intervention development including the final OCTOPuS manual as well as details on how to run the study. This training will be provided to all study sites prior to the commencement of the study at each site.

1.3 THE OCTOPUS SURVEY

In parallel to assessing the feasibility of the OCTOPus intervention, we developed and distributed a survey to understand the current practice for management of people with diabetes in cardiac surgery centres in the UK. The results of this survey will inform how the OCTOPuS study can fit into current NHS clinical practice.

2 RATIONALE & OBJECTIVES

There are currently two important uncertainties in the management of people with sub-optimally controlled diabetes undergoing intermediate and major surgery;

1) how to improve diabetes management in the weeks leading up to an elective procedure, and
2) whether that improved management is reflected in improved outcomes post-surgery (9).

Practice is therefore varied, with current UK guidelines recommending a delay to elective surgery to allow for improved diabetes management if HbA_{1c} is above 69 mmol/mol (where it is safe to do so); in contrast, the USA guidance recommends considering a delay to if the HbA_{1c} is above 53 mmol/mol. The current NICE guidelines recognise this as an evidence gap (10), as do the Joint British Diabetes Societies (3).

The Intervention Development part of the OCTOPuS study demonstrated that an outpatient intervention delivered to people with sub-optimally controlled diabetes before elective cardiac surgery is acceptable by people with diabetes and clinicians. The study demonstrated the feasibility of the recruitment process within the NHS routine pathway.

The present study aims to investigate whether the OCTOPuS intervention is both clinically and cost-effective at improving outcomes for people with sub-optimally controlled diabetes in a large-scale multi-centre Randomised Controlled Trial (RCT) involving approximately 15 cardiothoracic UK centres.

Specifically:

- We will assess whether the intervention can improve clinical outcomes following cardiac surgery.

For peer review only - http://bmjopen.bmj.com/site/about/guidelines.xhtml

- We will test whether the intervention can reduce HbA_{1c} levels in people awaiting cardiac surgery compared with usual care.
- Through qualitative and psychosocial research, we will explore the patient experience of receiving the intervention.
- We will undertake a health economic evaluation of the intervention.

3 STUDY DESIGN

OCTOPuS is a multicentre, parallel group, single blind, individually randomised trial incorporating a pre-planned futility analysis. It will compare time from surgery until an individual is clinically fit for discharge in adults with sub-optimally controlled type 1 or 2 diabetes undergoing elective surgery (as defined as an HbA_{1c} >53 mmol/mol for those ≤75 years old and > 64 mmol/mol for those > 75 years old) between the OCTOPuS intervention and usual care.

3.1 PRIMARY ENDPOINT

Time from surgery until clinically fit for discharge, as judged by the surgical team. Teams will be blinded to pre-hospital diabetes management allocation. This primary outcome was chosen because reduced time in hospital (though not at the expense of safety) is valued by people with diabetes, clinicians, and commissioners.

3.2 SECONDARY ENDPOINTS

- Time from surgery to actual discharge from hospital – this recognises that discharge can be delayed for non-clinical reasons
- Days alive between surgery and either out of hospital or judged as clinically fit for discharge
- Pre-operative mortality
- 30 day mortality
- 90 day mortality
- Time on ITU
- Time on a ventilator
- Sternal Wound Infections, defined as below according to the NICE guidance and the CDC criteria (19, 20):
  - Superficial Sternal Wound Infection, which involves skin, subcutaneous tissues and/or pectoralis fascia only without bone involvement
  - Deep Sternal Wound Infection, which involves the bone or mediastinum
- Leg wound infections, in patients who provide donor vein are graded according to the Centers for Disease Control and Prevention definitions of surgical site infections. Any sternal or leg wound infection occurring within three months after surgery will be considered as postoperative wound infections (21).
- Chest infections, defined as a change in typical chest symptoms (cough, increase respiratory rate, shortness of breath) in conjunction with a fever or inflammatory markers.
- Urinary tract infections, defined as ”clinically-diagnosed and treated, whether or not results from a urine culture are available’’
- Acute Coronary Syndrome, referring to any of these conditions leading to a hospital admission:
  - stable angina
  - unstable angina requiring or not nitrates infusion
  - non-ST elevation myocardial infarction
  - ST-elevation myocardial infarction (21)

For peer review only - http://bmjopen.bmj.com/site/about/guidelines.xhtml

- Change in weight between randomisation and surgery
- Effect on post-operative renal function and incidence of acute kidney injury as assessed by measurement of serum creatinine and calculation of estimated glomerular filtration rates
  - Acute Kidney Injury is defined as an increase in serum creatinine 1.5 -1.9 times the baseline level or serum creatinine increase ≥26.5 µmol/l within seven days after surgery (21).
- HbA_{1c} immediately preoperative, and at between 90 and 180 days post operation.
- Change in HbA_{1c} between baseline and immediately preoperative, and change from preoperative to between 90 and 180 days post operation
- Operations cancelled for sub-optimal glycaemic control
- Frequency and severity of self-reported overall, minor, severe and nocturnal hypoglycaemia assessed at Baseline, during the Support Contact and Pre-surgery according to the following definitions:
  - Overall hypoglycaemia is defined as any self-reported hypoglycaemia confirmed by a self-monitored capillary or interstitial glucose of <4 mmol/L
  - Minor hypoglycaemia is defined as any self-reported hypoglycaemia confirmed by a self-monitored capillary or interstitial glucose of <4 mmol/L when this was treated by the participant
  - Severe hypoglycaemia is defined as any self-reported episode of hypoglycaemia that required external assistance for treatment
  - Noctural hypoglycaemia is defined as any self-reported hypoglycaemia confirmed by a self-monitored capillary or interstitial glucose of <4 mmol/L that occurred between 0000 and 0600 (22).
- EQ-5D at baseline, 7 days and 90 days post-surgery. In the first phase of the trial, the utility of collecting EQ-5D at 30 days post-surgery will be explored. This has the potential for providing an extra data point, but the increased participant burden may risk the loss of completeness of the survey at 90 days.
- Psychosocial questionnaires at baseline and 90 days post-surgery to explore the impact of the intervention on factors important to quality of life and any changes to participants' diabetes self-management (see 7.6.2).
- Cost effectiveness of intervention

  Use of NHS lifestyle improvement programs and diabetes services

  Use of medication

  Time spent by practitioners for a) training, b) delivering the intervention and c) liaising with local services

  HbA_{1c} point-of-care and blood glucose monitoring costs

3.3 DEFINITION OF END OF STUDY

The last visit will be 180 days post-surgery for the last study participant who has a trial operation. The end of trial is defined as the date after the last patient has their last visit and when all data points required to answer the research question are captured and verified.

4 SELECTION AND ENROLMENT OF PARTICIPANTS

4.1 CONSENT

Consent to enter the study must be sought from each participant only after a full explanation has been given, an information leaflet offered and time allowed for consideration. Written informed participant consent should be obtained. The right of the participant to refuse to participate without giving reasons must be respected.

For peer review only - http://bmjopen.bmj.com/site/about/guidelines.xhtml

After the participant has entered the study, the clinician remains free to give alternative treatment to that specified in the protocol at any stage if he/she feels it is in the participant's best interest, but the reasons for doing so should be recorded. In these cases the participants remain within the study for the purposes of follow-up and data analysis. All participants are free to withdraw at any time from the protocol treatment without giving reasons and without prejudicing further treatment.

Participants will be asked to consent for long-term follow-up using routine data. We anticipate exploring (not as part of the current funding contract) outcomes such as 5 and 10 year mortality, and effect on longer term glycaemic control.

Cardiac surgery clinic lists will be scrutinised by the clinical cardiac surgery team to identify potentially eligible individuals who will be sent study information and receive a telephone call to discuss the study. Potential participants will have the opportunity to ask questions prior to their outpatient cardiac surgery appointment allowing at least 24 hours to consider the trial before their outpatient appointment.

A similar approach has been used by Foss et al (12), who demonstrated that people receiving telephone-based counselling about a trial showed similar levels of comprehension to those being counselled face-to-face. In the present study, potential participants will also have the opportunity to discuss the study face-to-face with the research nurse after their appointment with the cardiac surgeon and before consent is sought. See section 5.1 below for screening and consent procedures.

Upon completion of the informed consent form, a copy will be given to the participant, a copy stored in the participant's medical notes, a copy sent to the Southampton Clinical Trials Unit (SCTU) and the original filed in the Investigator Site File. The SCTU copy should be emailed to uhs.sctu@nhs.net using a secure nhs.net email address to allow for central monitoring.

4.2 INCLUSION CRITERIA

1. Aged ≥ 18 years old with type 1 or type 2 diabetes
2. Sub-optimally controlled diabetes defined as an HbA_{1c} > 53 mmol/mol for those ≤ 75 years old and an HbA_{1c} > 64 mmol/mol for those > 75 years old[9], measured using a near patient test at the cardiac surgery outpatient appointment where the decision to proceed to surgery is made.
3. Awaiting elective open-heart cardiac surgery
4. Anticipated delay before surgery of at least 2 months.
5. Surgery will take place at one of the hospitals participating in the trial
6. Ability to give informed consent.
7. Ability to interact with the study documentation and processes.

4.3 EXCLUSION CRITERIA

1. Active malignancy that would preclude engagement with OCTOPuS intervention[10]
2. Pregnancy
3. Previous cardiac surgery
4. Known haemoglobinopathies that affect the measurement of HbA_{1c}

[9] The higher cut-off in older people is designed to minimise the risk of iatrogenic hypoglycaemia in this population (17).
[10] Active malignancy is defined as malignancy which is currently being treated by chemotherapy, surgery or radiotherapy or is likely to cause death within 6 months

For peer review only - http://bmjopen.bmj.com/site/about/guidelines.xhtml

5. Other illnesses or conditions that would preclude engagement with the OCTOPuS intervention
6. Surgery taking place outside one of the participating hospitals, e.g. at a private hospital

4.4 SCREENING FAILURES

Screen failures will be recorded on the 'patient screening log'. This is completed for all individuals who have been considered for the study and is faxed or emailed monthly to the OCTOPuS Trial Team on 0844 774 0621 or octopus@soton.ac.uk.

4.5 REGISTRATION/RANDOMISATION PROCEDURES

After consent, a web-based remote randomisation system, maintained by Southampton CTU, will allocate participants to either the OCTOPuS intervention, or the control condition.

Participants will be randomised between the arms in a 1:1 ratio and stratified by centre, age (≤75 years old and >75 years old) and baseline HbA_{1c} (< 69 mmol/mol and ≥69 mmol/mol as per [3]), using pre-generated permuted blocks to prevent clinicians anticipating the allocation.

Participants randomised to the OCTOPuS intervention will see a health care professional trained in the OCTOPuS intervention.

4.6 CONTRACEPTION

Although it is not anticipated that there will be many pre-menopausal women in the study, women of child-bearing age will be advised to avoid pregnancy during the study.

5 STUDY OBSERVATIONS AND PROCEDURES

5.1 SCREENING PROCEDURES

Outpatient cardiac surgery appointment clinic lists will be scrutinised by a member of the cardiac surgery clinical team (or research nurse where he/she is a member of the clinical team) ahead of appointments, to identify those who may meet the study eligibility criteria. For people who appear eligible for the OCTOPuS study, an information sheet explaining the trial will be sent by post or email as appropriate. Before the outpatient appointment a member of the site trial research team will contact the prospective participant to discuss the study, allowing sufficient time for reflection and discussion (at least 24 hours) before the outpatient appointment. The information sheet will also include contact details to opt out if the person does not want to be contacted about the trial. This will allow those who are eligible for the study to be randomised immediately after the outpatient appointment, and where applicable receive their first OCTOPuS consultation, on the same day.

At the outpatient appointment where a decision to proceed to surgery is made, the treating surgeon will remind eligible patients about the trial. If the participant wishes to take part, a more detailed interview with a research nurse will follow, where the study can be discussed in depth according to the needs of the individual, and final exclusion criteria checked (e.g. pregnancy status) and written consent given.

Patients whose medical records cannot be accessed prior to the appointment to determine eligibility (e.g. patients from another hospital), will be given information about the study on the day of their outpatient appointment and will be offered the opportunity to come on another

For peer review only - http://bmjopen.bmj.com/site/about/guidelines.xhtml

day to discuss their participation in the trial. Where possible, the OCTOPuS visit will be scheduled for the same day to reduce the number of times that any individual has to travel to the trial centre.

The reason for this method of recruitment is because many cardiothoracic centres provide care to people living in a diverse geographical area and this will reduce the number of visits and length of time needed for travel to the trial centre.

5.2 STUDY PROCEDURES

Participants who are randomised to receive the OCTOPuS intervention will have an initial consultation with an OCTOPuS trained health professional (OCTOPuS Practitioner), who may be a doctor, nurse, pharmacist, or other appropriately trained person. In this consultation the participant's diabetes management will be discussed, as well as the likely benefits that improved glycaemic control will provide in the run up to surgery. The practitioner and participant will agree a number of actions, tailored to the individual needs and ability. These are likely to include:

- A graded exercise regimen. This may be completely self-delivered, or alternatively by joining a local appropriate exercise scheme – such as a 'health walk'. There is a general consensus amongst cardiac surgeons that limited exercise can be allowed prior to surgery. This needs to be individualised for each person and should not provoke symptoms of angina or breathlessness. The usual format of exercise suggested is walking on the flat, for short, frequent, episodes.
- Dietary advice, supplemented by a consultation with a dietitian if needed
- Medication review, which may lead to the introduction of insulin or other diabetes medications for people with type 2 diabetes.
- Specific advice about managing expectations, understanding facilitators to achieve change and overcoming barriers to improve medical and psychosocial outcomes

The exact process for this discussion and the treatment options are set out in the OCTOPuS intervention manual.

Participants will receive regular review with the OCTOPuS practitioner, probably by telephone, at least once a fortnight until the patient's diabetes management is optimised or no further changes are needed. After this, the frequency of the calls can be reduced at the discretion of the OCTOPuS practitioner to a minimum of every 6 weeks until surgery. This will be an opportunity to offer encouragement and support and address any issues which have arisen for the participant. One more support contact will be made between 1 and 3 weeks after discharge to ensure the continuity of the intervention beyond surgery. Although this final contact will not affect the primary outcome of the study, it was added following feedback from the participants in the intervention development study, who requested a final consultation before the diabetes management returned to their usual setting.

Where necessary the OCTOPuS practitioner will liaise with local services, e.g. the participant's GP or a dietitian, to facilitate delivery.

Participants in the control arm will receive usual care in the cardiac surgery centre attended by the individual. This is likely to contain brief advice from the patient's surgeon to pay attention to their diabetes in the run up to surgery. Some people may act on this advice, either on their own or in conjunction with their GP.

For peer review only - http://bmjopen.bmj.com/site/about/guidelines.xhtml

The study will document 'usual care' at all recruiting centres and explore with participants in the control arm as part of the qualitative work what actions were taken in response to advice received.

Discharge assessment: This should be completed by the surgical team who are blinded to the participant's pre-hospital diabetes management allocation.

5.3 FOLLOW UP

Following surgery all participants will be followed up at discharge, 7 days, 30 days and 90-180 days post-surgery. Also, as explained above a support call will be conducted between 1 and 3 weeks post-discharge. See 'SCHEDULE OF OBSERVATIONS AND PROCEDURES' for the information to be collected at each follow-up.

Most of the outcome measures will be collected in hospital, with the exception of 30 and 90 day mortality, which will either be collected through in-patient note review, or where possible using adult cardiac surgery databases (e.g. the SCTS National Adult Cardiac Surgery Audit).

Any of the following approaches can be used to obtain the 90-180 day HbA_{1c}.

- Many people have 3 – 6 monthly blood tests; for these individuals, the research team will ask for the copy of the result.
- For those attending cardiac surgery or cardiology outpatients in this window, the results of any outpatient clinic blood test result will be requested.
- The research team will contact the participant's GP to establish whether they have died, and if so the date of their death. If the person is still alive, they will be sent a capillary HbA_{1c} testing kit, which can be returned by post.

5.4 DEVIATIONS AND SERIOUS BREACHES

Any study protocol deviations/violations and breaches of Good Clinical Practice occurring at sites should be reported to the SCTU and the local R&D Office immediately. SCTU will then advise of and/or undertake any corrective and preventative actions as required.

All serious protocol deviations/violations and serious breaches of Good Clinical Practice and /or the study protocol will immediately be reported to the regulatory authorities and other organisations, as required in the Medicines for Human Use (Clinical Trials) Regulations 2004, as amended.

5.5 STUDY DISCONTINUATION

In consenting to the study, participants have consented to the study intervention, follow-up and data collection. Participants may be discontinued from the study procedures at any time.

Participants may be discontinued from the study in the event of:

- Permanent cancellation of surgery or postponement beyond 6 months of the original planned date of surgery
- Clinical decision, as judged by the Principal Investigator or Chief Investigator
- In the event the trial is discontinued due to the interim analysis (as outlined in Section 7.3)

Full details of the reason for study discontinuation should be recorded in the eCRF and medical record.

For peer review only - http://bmjopen.bmj.com/site/about/guidelines.xhtml

5.6 WITHDRAWAL

The participant / legal representative is free to withdraw consent from the study at any time without providing a reason.

Investigators should explain to the participants the value of remaining in study follow-up and allowing these data to be used for trial purposes. Where possible, those who have withdrawn from study treatment should remain in follow-up as per the trial schedule. If participants additionally withdraw consent for this, they should revert to standard clinical care as deemed by the responsible clinician. It would remain useful for the study team to continue to collect standard follow-up data and unless the participant explicitly states otherwise, follow-up data will continue to be collected.

Details of study discontinuation (date, reason if known) should be recorded in the eCRF and medical record.

5.7 PROHIBITED AND RESTRICTED THERAPIES DURING THE STUDY

There are no prohibited or restricted therapies during this study.

5.8 BLINDING AND PROCEDURES FOR EMERGENCY UNBLINDING

Due to the nature of the interventions in this trial it will not be possible to blind the participants or investigators, with the exception of discharge. Every attempt will be made to keep the surgical team assessing fitness to discharge blinded to the treatment allocation. As this assessment does not depend on treatment allocation, it will not be necessary to unblind the surgeons.

6 SAFETY

6.1 DEFINITIONS

Adverse Event (AE): any untoward medical occurrence in a participant or clinical study participant which does not necessarily have a causal relationship with study treatment or participation.

An AE can therefore be any unfavourable and unintended sign (including an abnormal laboratory finding), symptom, or disease temporally associated with the study treatment or participation (regardless of causality assessments).

Serious Adverse Event (SAE) is any untoward medical occurrence or effect that:

- **Results in death**
- **Is life-threatening***
- **Requires hospitalisation, or prolongation of existing hospitalisation****
- **Results in persistent or significant disability or incapacity**
- **Is a congenital anomaly or birth defect**
- Other important medical events***.

*'life-threatening' in the definition of 'serious' refers to an event in which the participant was at risk of death at the time of the event; it does not refer to an event which hypothetically might have caused death if it were more severe.

For peer review only - http://bmjopen.bmj.com/site/about/guidelines.xhtml

**Hospitalisation is defined as an in-patient admission, regardless of length of stay, even if the hospitalisation is a precautionary measure for continued observation. Hospitalisations for a pre-existing condition, including elective procedures that have not worsened, do not constitute an SAE. Prolongation of hospitalisation is considered to be the case from >30 days post-cardiothoracic surgery i.e. hospitalisation for surgery and up to 30 days is not considered an SAE.

***Other important medical events may also be considered serious if they jeopardise the participant or require an intervention to prevent one of the above consequences.

Note: It is the responsibility of the PI or delegate to grade an event as ‘not serious’ (AE) or ‘serious’ (SAE).

6.2 SERIOUSNESS

A complete assessment of the seriousness must always be assessed by a medically qualified doctor who is registered on the delegation of responsibility log; this is usually the investigator.

All adverse events that fulfil the criteria definition of ‘serious’ in protocol section 6.1, must be reported to SCTU using the Serious Adverse Event Report Form – Non-CTIMP. Specific exceptions to this (as listed below) should be recorded as AEs rather than SAEs.

All SAEs must be reported immediately by the PI at the participating centre to the SCTU.

6.2.1 Exceptions:

For the purposes of this study, the following SAEs **do not** require reporting to SCTU using the Serious Adverse Event Report Form – Non-CTIMP:

- Hospitalisations for elective treatment of a pre-existing condition

Also, the following SAEs **do not** require reporting to SCTU using the Serious Adverse Event Report Form – Non-CTIMP if they occur between ‘Surgery’ and ‘Discharge’:

- Arrhythmia, including atrial fibrillation
- Immediate postoperative surgical bleeding
- Pneumonia

6.3 CAUSALITY

A complete assessment of the causality must always be assessed by a medically qualified doctor who is registered on the delegation of responsibility log; this is usually the investigator.

If any doubt about the causality exists the local investigator should inform the SCTU who will notify the Chief Investigator. Other clinicians may be asked for advice in these cases.

Relationship	Description	Event Status
**Unrelated**	There is no evidence of any causal relationship	Not related to treatment
**Unlikely**	There is little evidence to suggest there is a causal relationship (e.g. the event did not occur within a reasonable time after administration of the study treatment). There is another reasonable explanation for	Not related to treatment

For peer review only - http://bmjopen.bmj.com/site/about/guidelines.xhtml

	the event (e.g. the participant's clinical condition, other concomitant treatment).	
**Possibly**	There is some evidence to suggest a causal relationship (e.g. because the event occurs within a reasonable time after administration of the study treatment). However, the influence of other factors may have contributed to the event (e.g. the participant's clinical condition, other concomitant treatments).	Related and expected SAE/ Related and unexpected SAE
**Probably**	There is evidence to suggest a causal relationship and the influence of other factors is unlikely.	Related and expected SAE/ Related and unexpected SAE
**Definitely**	There is clear evidence to suggest a causal relationship and other possible contributing factors can be ruled out.	Related and expected SAE/ Related and unexpected SAE

In terms of event status; **Not related to treatment** would highlight that the SAE is not related to the trial treatment. **Related and expected** SAE would signify that the SAE is related to the trial treatment and is expected (according to the list of expected events listed in the protocol). **Related and unexpected SAE** would be classified as an SAE which is related to the trial treatment and is unexpected in terms of the events listed in the protocol.

In the case of discrepant views on causality between the Investigator and others, SCTU will classify the event as per the worst case classification and where applicable the Ethics Committee will be informed of both opinions within the required timelines.

6.4 EXPECTEDNESS

Expectedness assessments are made against the list of expected events below:

6.4.1 Expected Adverse Events:

- Minor musculoskeletal aches and pains
- Myocardial infarction
- Respiratory tract infection

The nature or severity of should be considered when making the assessment of expectedness. If these factors are not consistent with the current information available then the AE should be recorded as 'unexpected'.

6.5 REPORTING PROCEDURES

All adverse events should be reported until the End of Study as defined in 3.3.

Depending on the nature of the event, the appropriate reporting procedures below should be followed. A flowchart will be provided to aid in the reporting procedures.

6.5.1 Reporting Details

A SAE for Non-CTIMPs Form should be completed for all SAEs and faxed to SCTU within 24 hours of site becoming aware of the event.

Complete the SAE form and fax or email a scanned copy of the form with as many details as possible to the SCTU together with anonymised relevant treatment forms and investigation reports.

For peer review only - http://bmjopen.bmj.com/site/about/guidelines.xhtml

Or
Contact the SCTU by phone for advice and then fax or email a scanned copy of the completed SAE form.

SAE REPORTING CONTACT DETAILS
Please email or fax a copy of the SAE form to SCTU within 24 hours of becoming aware of the event
**Fax: 0844 774 0621 or Email: ctu@soton.ac.uk**
FAO: Quality and Regulatory Team
For further assistance: Tel: 023 8120 4138 (Mon to Fri 09:00 – 17:00)

Additional information should be provided as soon as possible if the event has not resolved at the time of reporting.

6.5.2 Follow Up and Post- study SAEs

The reporting requirement for all AEs and SAEs affecting participants applies for all events occurring up to 90 days following cardiac surgery.

All unresolved adverse events should be followed by the investigator until resolved, the participant is lost to follow-up, or the adverse event is otherwise explained. At the last scheduled visit, the investigator should instruct each participant to report any subsequent event(s) that the participant, or the participant's general practitioner, believes might reasonably be related to participation in this study. The investigator should notify the study sponsor of any death or adverse event occurring at any time after a participant has discontinued or terminated study participation that may reasonably be related to this study.

6.5.3 Pre-existing Conditions

Medically significant pre-existing conditions (those which are present prior to informed consent) should not be reported as an AE unless the conditions worsens during the trial. The condition, however, must be reported on the Medical History eCRF. Any adverse events which occur after informed consent taken should be recorded on the AE eCRF as per safety reporting section.

6.5.4 Serious Adverse Events

All SAEs should be reported within 24 hours of the local site becoming aware of the event. The SAE Non-CTIMP Form asks for nature of event, date of onset, severity, corrective therapies given, outcome, causality (i.e. unrelated, unlikely, possible, probably, definitely) and expectedness. The responsible investigator should assign the causality and expectedness of the event with reference to the events listed in Section 6.4.1. The event term should be in accordance with the latest version of MedDRA and grades given in accordance with the NCI CTCAE v5, Additional information should be provided as soon as possible if the event has not resolved at the time of reporting.

For peer review only - http://bmjopen.bmj.com/site/about/guidelines.xhtml

6.6 SCTU RESPONSIBILITIES FOR SAFETY REPORTING TO REC

SCTU will notify the necessary competent authorities of all **Related and Unexpected** SAEs occurring during the study within 15 days.

SCTU submit all safety information to the REC in annual progress report.

7 STATISTICS AND DATA ANALYSES

7.1 METHOD OF RANDOMISATION

Participants will be individually randomised between the arms, using a 1:1 allocation ratio. The randomisation will be performed via an online system allowing instant assignment to groups 24 hours per day. The service will be provided by Southampton Clinical Trials Unit with telephone back up during office hours (9am-5pm) on days when the University of Southampton is open. Randomisation will be stratified by centre, age and baseline HbA_{1c} and use permuted blocks.

7.2 SAMPLE SIZE

7.2.1 Futility Assessment – Physiological Effect of Intervention

To demonstrate that a physiological response is plausible we need to show an HbA_{1c} improvement of 5 mmol/mol in the intervention group at pre-surgery compared to baseline. Previous experience shows the mean initial HbA_{1c} in our study population to be around 72 mmol/mol, with a standard deviation of around 15 mmol/mol (13,14). For an expected change in HbA_{1c} from baseline of 5 mmol/mol in the intervention group, and assuming a conservative correlation of 50% between baseline and pre-surgery, a sample size of 50 participants would allow a margin of error of 4.16 below the mean for a 95% confidence interval (CI). This CI width would allow us to exclude a difference of zero if the treatment difference of 5 was observed.

7.2.2 Intervention effectiveness – clinical outcomes

The primary outcome is the time from surgery to when the responsible consultant considers the participant clinically fit for discharge. We will not consider the actual discharge date in the primary analysis, as currently in cardiothoracic surgery many elective patents are kept in hospital longer than clinically indicated due to their social situation. Discussions with clinicians and commissioners suggest that a mean improvement of half a day would be clinically worthwhile.

The current mean duration post-surgery until clinically fit for discharge is 7 days, with a standard deviation of 1.5 days. To demonstrate an improvement of 0.5 days with 90% power and 5% significance with 1:1 randomisation between intervention and control arms would require a total of 382 participants (nQuery v7.0). After listing for surgery, very few patients are lost to follow-up. We will therefore allow for a 5% loss to follow-up, and a further 5% for deaths post randomisation inflating the final target sample size to 426 participants.

7.3 INTERIM ANALYSIS

The futility will be assessed, and there is the potential that the trial could be stopped early in one of two ways:

7.3.1 Recruitment and Delivery

There are several threats to recruitment and delivery of this trial:

- Being unable to recruit and initiate sufficient centres.

For peer review only - http://bmjopen.bmj.com/site/about/guidelines.xhtml

- Centres not being able to recruit sufficient participants
- Centres not being able to deliver the OCTOPuS interventions

Therefore throughout the trial phase of the study we will review progress against a number of criteria at three time points, grading trial progress as red, amber, or green each time. Appendix 1, Table 1 sets out the actions to be taken depending on how progress is scored. Appendix 1, Table 2 describes the criteria leading to the scores.

7.3.2 Physiological Effect of Intervention

It is believed that the OCTOPuS intervention will have its clinically relevant effects (such as reducing length of stay, reducing infection and reducing mortality) through improvement of a number of clinical variables, including change in body weight, exercise, lipid profile and blood pressure. However, as the main target of the intervention is to improve glycaemic control, if no physiological effect can be demonstrated on glycaemic measures, continuation of the trial would be futile. After the first 100 participants have had their surgery we will assess the effect of the intervention on pre-operative HbA_{1c} as stated in Appendix 1. If there is no discernible effect (defined as a change of HbA_{1c} of < 5 mmol/mol) we will ask the TSC to review the trial's viability.

7.3.3 Other reasons for stopping the trial early

While previous experience has suggested that in a different clinical group a 3 day saving in length of stay is possible, advice from clinicians, commissioners, and service managers is that a 0.5 day reduction in length of stay would be worthwhile. The trial is therefore powered on a 0.5 day reduction, however, the DMEC will be asked to periodically review the effect being observed and to recommend a change in sample size or stop the trial if appropriate.

7.4 STATISTICAL ANALYSIS PLAN (SAP)

Since this is a parallel group, randomised controlled trial, with a usual care (control) arm, data will be reported and presented according to the revised CONSORT statement (15, 16). A detailed statistical analysis plan will be developed prior to the final analysis of the trial, however, the main features of the plan are discussed below.

Demographics and characteristics of participants at baseline will be summarised and assessed for comparability between the intervention and control arms (16). The primary analysis will be conducted using ANCOVA adjusted for randomisation stratification factors on an intention to treat population. Continuous data will be presented as means and standard deviations and analysed using ANCOVA (or presented as medians and ranges and analysed using Mann-Whitney U tests if data are skewed). Binary data will be reported in terms of odds ratios and analysed using logistic regression modelling. Analysis of time-to-event outcomes will include presenting Kaplan-Meier graphs by arm and analysed using Cox proportional hazards regression (or competing risk regression as discussed below). A two-sided p-value of 0.05 or less will be used to declare statistical significance for all analyses and results will be presented with 95% confidence intervals.

Subgroups will be investigated, including those with HbA_{1c} above or below 69 mmol/mol at presentation; type of diabetes; age above or below 75 years. The cut-off of 69 mmol/mol has been chosen as the level above which the Joint British Diabetes Societies recommend specific

For peer review only - http://bmjopen.bmj.com/site/about/guidelines.xhtml

action to improve pre-operative glycaemic control. The cut-off for age has been chosen to reflect the different HbA_{1c} entry criteria for those above and below 75 years.

It is possible that a small proportion of participants will receive the intervention/usual care but will not actually undergo the planned surgery due to death, or clinically directed surgery cancellation. A small proportion may also undergo urgent revascularisation due to myocardial infarction after they have received their allocated treatment. A further group may undergo surgery but die before they are well enough for discharge from hospital and thus not meet the primary endpoint. In total, it is expected that these events will occur in no more than 5% of participants. In this case, these individuals will be excluded from the primary analysis but the prevalence of each of these outcomes will be monitored and recorded by treatment arm separately and presented to the DMEC for their guidance if there is any indication that there is an excess of any of these outcomes in either treatment group. A sensitivity analysis will be considered, looking at a competing risks model, where any of these outcomes and functional recovery are competing risks. This sensitivity analysis will also be performed if the total prevalence of these events is more than 5%. The analysis plan for the psychosocial questionnaires is detailed in section 7.6.2 below.

7.5 ECONOMIC EVALUATION

Within-trial analysis, with longer term modelling, will be used to estimate the cost-effectiveness of the intervention compared with usual care. The analysis will follow a NICE 'reference case', with costs estimated from a healthcare perspective and outcomes quantified using Quality Adjusted life Years (QALYs).

The 'within-trial' analysis will be conducted using data on healthcare use, mortality and health-related quality of life ('utility'); covering the period from randomisation to 90 days post-surgery. Utility will be measured with the patient-reported **EQ-5D-5L questionnaire**, at baseline, 7, 30 and 90 days post-operative. After the first 100 patients have been recruited, it will be examined whether an EQ-5D-5L taken at 30 days post-surgery is both useful and a reasonable patient burden. If so, we will continue to collect EQ-5D-5L at this timepoint.

Intervention costs will be estimated, including: practitioner training time; practitioner time for delivery of the initial consultation, telephone follow-up and liaison with local services (also see 'Study Observations and Procedures' table). Participants in both groups will be asked about use of related NHS services before surgery, including changes to medication for diabetes, consultations with GP, dietitian or other clinicians, and participation in lifestyle interventions, such as exercise referral schemes (also see 'Study Observations and Procedures' table). It is important to collect this information for both study groups, to estimate the net cost to the NHS of delivering the intervention. Hospital care or treatment for any surgical complications will be collected in the CRF. For study efficiency, and to minimise burden on the participants, post-discharge data will not be collected on healthcare use from participants, although total NHS costs will be estimated in the modelling approach described below. Unit costs for all staff and services will be obtained from routine national sources (NHS Reference Costs, PSSRU estimates and BNF or Drug Tariff for medications).

Methods of analysis for the economic trial data will be pre-defined alongside the statistical analysis plan. QALYs will be estimated from EQ-5D-5L and mortality data, using the area-under-the-curve method. Similarly, costs will be estimated at the patient level. Mean between-group differences in QALYs and costs will be estimated using a regression-based approach, including adjustment for baseline covariates and interaction terms for pre-defined sub-groups, and allowing for clustering at hospital and/or practitioner level. Results will be presented as an

For peer review only - http://bmjopen.bmj.com/site/about/guidelines.xhtml

Incremental Cost-Effectiveness Ratio (ICER) if appropriate. Non-parametric bootstrapping will be used to estimate confidence intervals around estimated cost differences and ICERs.

A simple modelling approach will be also be used to estimate the costs and health impacts of surgical complications over a lifetime horizon. This long extrapolation is necessary to reflect any mortality or lasting quality of life decrement associated with surgical complications. There will be no attempt to estimate the long-term impact of improved diabetes management related to the intervention, as it will be difficult to predict the duration over which any improvements will be maintained. This is likely to be a conservative assumption that will under-estimate the QALY gain and cost-effectiveness of intervention if it proves to be effective. Model parameters will be estimated from the trial and from other published sources. Long-term resource use, mortality and utility decrements associated with key surgical complications, will be identified by systematic review of HTAs, NICE guidelines and published literature.

Permission to use the EQ-5D measure has been granted by the EuroQol Research Foundation.

7.6 QUALITATIVE & PSYCHOSOCIAL OUTCOMES

In addition to the descriptive, biomedical and physiological data collected and analysed, qualitative interviews and validated psychosocial measures will be used to understand and explore:

- Participants' experiences of the intervention and health professionals' views about delivering it.
- The perceived benefits of the intervention from participants' and health professionals' perspectives; and, their recommendations for future refinements.
- Any changes participants make to their diabetes self-management practices and treatment goals after receiving the intervention and 90 days after surgery, and why.
- Whether there are any site-specific differences in how participants self-manage their diabetes after receiving the intervention, and why.

7.6.1 Interviews

Qualitative interviews with participants, those who decline to participate/drop out and healthcare professionals will explore perceptions and experiences of the intervention and how it might be improved. Treatment fidelity will be maintained by including all content to be covered on checklists, assessed by intervention access/usage and examining any diversions from the protocol.

7.6.1.1 Participant interviews

Fifty participants receiving the intervention will be recruited across all participating sites. Purposive sampling will be used so that there is diversity in terms of age, gender, diabetes duration, treatment type and occupation in the final sample. Baseline interviews will take place within 2 weeks of participants' commencing the intervention and will explore their experiences; any changes made to their diabetes self-management practices, and why; short- and long-term treatment goals and the reasons for these; and perceived barriers and facilitators to achieving these goals. These interviews will also include detailed exploration of participants' historical diabetes management practices; previous contact with health professionals and diabetes management programmes; and, their everyday work and family lives. After the first 3-4 interviews have been conducted, these interviews will be reviewed so that revisions can to be made to the topic guide if these are required.

For peer review only - http://bmjopen.bmj.com/site/about/guidelines.xhtml

After all baseline interviews have been completed, the team will undertake an interim analysis and then meet to: (a) discuss preliminary findings; (b) agree on a coding frame; and (c) develop and agree on a topic guide for the follow-up interviews.

The follow-up interviews will be conducted with the same participants at 90 days post-surgery to explore whether, how and, why, their diabetes self-management practices and treatment goals have changed in the intervening period; and, any perceived barriers to achieving future changes and goals. The interviews will also explore participants' information and support needs and whether, and in what ways, the intervention and follow-up care could be changed or improved.

Both interviews may be audio-recorded and brief quotations may be included in study reports. Nobody will be able to identify any participant in these reports. Audio-recordings and subsequent transcripts of the interviews will be stored for up to 15 years after the end of this study for research review purposes and will be held securely.

7.6.1.2 Health professional interviews

Key personnel involved in the delivery will be interviewed once around 12 months after the start of trial in their centre[11]. Interviews will explore: previous experiences; perceived benefits as compared to routine care; experiences of, and views about the intervention; barriers and facilitators to intervention delivery; perceived impact of the intervention on participants' diabetes self-management practices; and, any suggested improvements for future use.

Data analysis will commence as soon as data collection begins. Regular meetings will be held between the team to discuss preliminary findings, make refinements to the topic guides if required and to agree on a coding frame. A thematic approach will be used to analyse the data, the purpose of which is to look for, and understand, patterns and experiences which cut across different people's accounts and the reasons for these. Key aspects of the analysis will include: (a) comparisons between participants' baseline and follow-up interviews to identify changes in their perceptions, experiences and diabetes self-management practices over time, and the reasons for these; (b) comparison of participant and health professional accounts to identify similarities and differences in their understandings and any impact on diabetes self-management practices; (c) cross-comparison of participants' accounts to identify common issues and experiences as well differences in diabetes self-management practices between subgroups of participants (e.g. men versus women, participants of different ages etc.), and the reasons for these.

7.6.1.3 Quality procedures

Several quality procedures will be used to increase the validity and credibility. Procedures to be used are for instance: using a member check, the use of several data collection methods (triangulation), using a reflexive diary, doing the analyses by two researchers and the use of 'thick descriptions'.

The data from both the participant and health professionals interviews will be collected outside of the study database and will be analysed by qualitative researchers who are part of the study team.

---

[11] Please note: as the timing is independent from the study visits and will vary for each site, the health professional interviews are not included in the 'Schedule of Interventions and Procedures'.

For peer review only - http://bmjopen.bmj.com/site/about/guidelines.xhtml

7.6.2 *Psychosocial Questionnaires*

The following questionnaires will be completed by participants at baseline and at 3 months post-surgery to explore the perceived benefits of the intervention, any changes to participants' diabetes self-management and associations between psychosocial factors and the primary endpoint:

- **Diabetes Empowerment Scale (short form)**: an 8 item questionnaire assessing diabetes-related psychosocial self-efficacy. License (to be) purchased by Mapi Research Trust.
- **PAID5**: a 5 item self-reported measure of diabetes related distress with high internal consistency. Accessed from McGuire et al. (2010), permission obtained from main author.
- **Patient Health Questionnaire (PHQ-2)**: ultra-brief depression screener, variant of PHQ-9. It is not used to establish a final diagnosis or to monitor depression severity but rather to screen for depression as a 'first step' approach. Adapted from PHQ9, freely available from the public domain.
- **Brief Illness Perception Questionnaire (B-IPQ)**: a 8 item measure assessing cognitive illness representations, emotional representations, illness comprehensibility and perceived causal factors for illness. Accessed from the website, permission obtained from Elizabeth Broadbend. The final item of the B-IPQ is qualitative and will not be collected in this study.
- **Summary of Diabetes Self-Care Activities scale (SDSCA)**: a 15-item self-report questionnaire of diabetes self-management that includes items assessing the following aspects of the diabetes regimen: general diet, specific diet, exercise, blood-glucose testing, foot care, and smoking. It is a brief yet reliable and valid self-report measure of diabetes self-management that is useful both for research and practice. Permission was obtained by Oregon Research Institute.

The analysis of the questionnaire responses will aim to answer the following questions:

1. What effect does baseline score (categorised as high/low etc. as appropriate) have on study outcomes, i.e. days until considered fit for surgery?
2. What effect does the study intervention have on change in score assessed as a continuous variable from baseline to 90 days post-surgery?
3. Does the treatment work better or less well in people depending on their baseline score (categorised)?

8 REGULATORY

8.1 CLINICAL TRIAL AUTHORISATION

This study is not considered to be a clinical trial of a medicinal product, so clinical trial authorisation from the UK Competent Authority the Medicines and Healthcare products Regulatory Agency (MHRA) is not applicable.

9 ETHICAL CONSIDERATIONS

The study will be conducted in accordance with the recommendations for physicians involved in research on human participants included in the WMA Declaration of Helsinki, as amended by the 64th WMA General Assembly, Fortaleza, Brazil, October 2013 and as revised and recognised by governing laws and EU Directives. Each participant's consent to participate in the study should be obtained after a full explanation has been given of treatment options, including the conventional and generally accepted methods of treatment. The right of the participant to refuse to participate in the study without giving reasons must be respected.

For peer review only - http://bmjopen.bmj.com/site/about/guidelines.xhtml

After the participant has entered the study, the clinician may give alternative treatment to that specified in the protocol, at any stage, if they feel it to be in the best interest of the participant. However, reasons for doing so should be recorded and the participant will remain within the study for the purpose of follow-up and data analysis according to the treatment option to which they have been allocated. Similarly, the participant remains free to withdraw at any time from protocol treatment and study follow-up without giving reasons and without prejudicing their further treatment.

9.1 SPECIFIC ETHICAL CONSIDERATIONS

Our proposal to randomise participants on the same day as the decision to proceed to surgery is unusual. However, it is believed that this is entirely justified as (i) this is a pragmatic approach, which reflects how the intervention would be used in routine practice, (ii) it is more convenient for the participants, relieving them on an additional journey to a potentially remote hospital, (iii) this approach to consent has been used previously within the European Union (12), and (iv) this approach was feasible and acceptable to participants during the intervention development study. All participants will have an opportunity to discuss the study face-to-face with a clinician before randomisation, and will able to withdraw should they change their mind.

Participants will be reassured that all personally identifiable data collected during the course of the research will be kept strictly confidential, and non-identifiable data will be shared in accordance with the University of Southampton policies. All participant data will be anonymised and stored on a database in accordance with current Data Protection Regulations. We will also seek the participant's permission to inform their general practitioner that they are taking part in this study. Documentation relating to clinical trials managed by Southampton CTU is retained for 15 years after notification of the trial's end.

9.2 ETHICAL APPROVAL

Ethical approval for this study protocol will be sought by from Research Ethics Committee or Institutional Review Board (IRB) in the approved national participating countries.

9.3 INFORMED CONSENT PROCESS

Informed consent is a process that is initiated prior to an individual agreeing to participate in a study and continues throughout the individual's participation. In obtaining and documenting informed consent, the investigator should comply with applicable regulatory requirements and should adhere to the principles of GCP.

Discussion of objectives, risks and inconveniences of the study and the conditions under which it is to be conducted are to be provided to the participant by appropriately delegated staff with knowledge in obtaining informed consent with reference to the patient information leaflet. This information will emphasise that participation in the trial is voluntary and that the participant may withdraw from the trial at any time and for any reason. The participant will be given the opportunity to ask any questions that may arise and provided the opportunity to discuss the study with family members, friend or an independent healthcare professional outside of the research team and time to consider the information prior to agreeing to participate.

9.4 CONFIDENTIALITY

SCTU will preserve the confidentiality of participants taking part in the study. The investigator must ensure that participant's anonymity will be maintained and that their identities are

For peer review only - http://bmjopen.bmj.com/site/about/guidelines.xhtml

protected from unauthorised parties. On CRFs participants will not be identified by their names, but by an identification code.

10 SPONSOR

SCTU, Chief Investigator and other appropriate organisations have been delegated specific duties by the Sponsor and this is documented in the trial task allocation matrix.

The duties assigned to the study sites (NHS Trusts or others taking part in this study) are detailed in the Non-Commercial Agreement.

10.1 INDEMNITY

For NHS sponsored research HSG (96) 48 reference no.2 applies. If there is negligent harm during the clinical study when the NHS body owes a duty of care to the person harmed, NHS Indemnity covers NHS staff, medical academic staff with honorary contracts, and those conducting the study. NHS Indemnity does not offer no-fault compensation and is unable to agree in advance to pay compensation for non-negligent harm. Ex-gratia payments may be considered in the case of a claim.

10.2 FUNDING

This study is funded by the National Institute for Health Research Health Technology Assessment Programme (16/25/12).

10.2.1 Site payments

The payments assigned to the study sites (NHS Trusts or others taking part in this study) are detailed in the Non-Commercial Agreement.

This study is automatically eligible for the NIHR portfolio. This enables Trusts to apply to their comprehensive local research network for service support costs, if required.

10.2.2 Participant payments

Participants will not be paid for participation in this study but reasonable travel expenses will be refunded for trial related activities.

10.3 AUDITS AND INSPECTIONS

The study may be inspected and audited by UHS (under their remit as Sponsor), SCTU (as the Sponsor's delegate) and other regulatory bodies to ensure adherence to the principles of GCP, Research Governance Framework for Health and Social Care, applicable contracts/agreements and national regulations.

11 STUDY OVERSIGHT GROUPS

The day-to-day management of the trial will be co-ordinated through the SCTU and oversight will be maintained by the Trial Management Group, the Trial Steering Committee and the Data Monitoring and Ethics Committee.

For peer review only - http://bmjopen.bmj.com/site/about/guidelines.xhtml

11.1 TRIAL MANAGEMENT GROUP (TMG)

The TMG is responsible for overseeing progress of the study, including both the clinical and practical aspects. The Chair of the TMG will be the Chief Investigator of the study.

The OCTOPuS TMG charter defines the membership, terms of reference, roles, responsibilities, authority, decision-making and relationships of the TMG, including the timing of meetings, frequency and format of meetings and relationships with other trial committees.

11.2 TRIAL STEERING COMMITTEE (TSC)

The TSC acts as the oversight body on behalf of the Sponsor and Funder. The TSC will meet twice a year. The majority of members of the TSC, including the Chair, should be independent of the study.

The OCTOPuS TSC charter defines the membership, terms of reference, roles, responsibilities, authority, decision-making and relationships of the TSC, including the timing of meetings, frequency and format of meetings and relationships with other trial committees.

11.3 INDEPENDENT DATA MONITORING COMMITTEE (IDMC) / DATA MONITORING AND ETHICS COMMITTEE (DMEC)

*(**NB** for the purposes of this protocol, IDMC and DMEC refer to the same committee, and these terms can be used interchangeably).*

The aim of the IDMC is to safeguard the interests of study participants, monitor the main outcome measures including safety and efficacy, and monitor the overall conduct of the study.

There is a theoretical risk that the intervention – especially the exercise component - may induce myocardial infarction, and hence possibly death. When participants have a myocardial infarction while on a waiting list for cardiac surgery, the most common outcome is survival with urgent revascularisation. Adverse outcomes will be collected, and the Data Monitoring and Ethics committee will be asked to review myocardial infarction rates and deaths for participants enrolled in the trial. If the DMEC believes that the intervention is causing excessive morbidity and mortality they will recommend stopping the trial.

The OCTOPuS DMEC charter defines the membership, terms of reference, roles, responsibilities, authority, decision-making and relationships of the IDMC, including the timing of meetings, methods of providing information to and from the IDMC, frequency and format of meetings, statistical issues and relationships with other trial committees.

12 DATA MANAGEMENT

Participant data will be entered remotely at site and retained in accordance with current Data Protection Regulations. The PI is responsible for ensuring the accuracy, completeness, and timeliness of the data entered.

The participant data are pseudo-anonymised by assigning each participant a participant identifier code which is used to identify the participant during the study and for any participant-specific clarification between SCTU and site. The site retains a participant identification code list which is only available to site staff.

For peer review only - http://bmjopen.bmj.com/site/about/guidelines.xhtml

The Informed Consent Form will specify the participant data to be collected and how it will be managed or might be shared; including handling of all Patient Identifiable Data (PID) and sensitive PID adhering to relevant data protection law.

Trained personnel with specific roles assigned will be granted access to the electronic case report forms (eCRF). eCRF completion guidelines will be provided to the investigator sites to aid data entry of participant information.

Only the Investigator and personnel authorised by them should enter or change data in the eCRFs. When requested, laboratory data must be transcribed, with all investigator observations entered into the eCRF. The laboratory reports (original or copies) must be retained by the Investigator for future reference.

A Data Management Plan (DMP) providing full details of the study specific data management strategy for the trial will be available and a Trial Schedule with planned and actual milestones, CRF tracking and central monitoring for active trial management created.

Data queries will either be automatically generated within the eCRF, or manually raised by the study team, if required. All alterations made to the eCRF will be visible via an audit trail which provides the identity of the person who made the change, plus the date and time.

At the end of the study after all queries have been resolved and the database frozen, the PI will confirm the data integrity by electronically signing all the eCRFs. The eCRFs will be archived according to SCTU policy and a PDF copy including all clinical and Meta data returned to the PI for each participant.

Data may be requested from the Data Access Committee at SCTU. Request will be considered on a monthly basis.

Please note that the qualitative interview data will be not be collected as part of the study database.

12.1 DATA SHARING

In order to meet our ethical obligation to responsibly share data generated by interventional clinical trials, SCTU operate a transparent data sharing request process for results that are available in the public domain. As a minimum, anonymous data will be available for request from three months after publication of an article, to researchers who provide a completed Data Sharing request form that describes a methodologically sound proposal, for the purpose of the approved proposal and if appropriate a signed Data Sharing Agreement. Data will be shared once all parties have signed relevant data sharing documentation.

Researchers interested in our data are asked to complete the Request for Data Sharing form (CTU/FORM/5219) [template located on the SCTU web site, www.southampton.ac.uk/ctu] to provide a brief research proposal on how they wish to use the data. It will include; the objectives, what data are requested, timelines for use, intellectual property and publication rights, data release definition in the contract and participant informed consent etc. If considered necessary, a Data Sharing Agreement from Sponsor may be required.

For peer review only - http://bmjopen.bmj.com/site/about/guidelines.xhtml

13 MONITORING

13.1 CENTRAL MONITORING

Data stored at SCTU will be checked for missing or unusual values (range checks) and checked for consistency within participants over time. Any suspect data will be returned to the site in the form of data queries. Data query forms will be produced at SCTU from the trial database and sent either electronically or through the post to a named individual (as listed on the site delegation log). Sites will respond the queries providing an explanation/resolution to the discrepancies and return the data query forms to SCTU. The forms will then be filed along with the appropriate CRFs and the appropriate corrections made on the database. There are a number of monitoring features in place at SCTU to ensure reliability and validity of the trial data, which are detailed in the trial monitoring plan.

The DMEC also have responsibility for specific central monitoring activities, as described in protocol section 11.3.

13.2 CLINICAL SITE MONITORING

Monitoring will be completed as per the trial monitoring plan.

13.2.1 Source Data Verification

On receipt of a written request from SCTU, the PI will allow the SCTU direct access to relevant source documentation for verification of data entered onto the eCRF (taking into account data protection regulations). Access should also be given to study staff and departments.

The participants' medical records and other relevant data may also be reviewed by appropriate qualified personnel independent from the SCTU appointed to audit the study, including representatives of the Competent Authority. Details will remain confidential and participants' names will not be recorded outside the study site.

13.3 SOURCE DATA

Source documents are where data are first recorded, and from which participants' CRF data are obtained. These include, but are not limited to, hospital records (from which medical history and previous and concurrent medication may be summarised), clinical and office charts, laboratory and pharmacy records, diaries, microfiches, radiographs, and correspondence.

14 RECORD RETENTION AND ARCHIVING

Trial documents will be retained in a secure location during and after the trial has finished.

The PI or delegate must maintain adequate and accurate records to enable the conduct of the study to be fully documented and the study data to be subsequently verified. After study closure the PI will maintain all source documents and study related documents. All source documents will be retained for a period of 15 years following the end of the study.

Sites are responsible for arching the ISF and participant's medical records. The Sponsor is responsible for archiving the TMF and other relevant documentation.

15 PUBLICATION POLICY

Data from all centres will be analysed together and published as soon as possible.

For peer review only - http://bmjopen.bmj.com/site/about/guidelines.xhtml

Individual investigators may not publish data concerning their participants that are directly relevant to questions posed by the trial until the Trial Management Group (TMG} has published its report. The TMG will form the basis of the Writing Committee and advise on the nature of publications. All publications shall include a list of investigators, and if there are named authors, these should include the Chief Investigator, Co-Investigators, Trial Manager, and Statistician(s) involved in the trial. Named authors will be agreed by the CI and Director of SCTU. If there are no named authors then a 'writing committee' will be identified.

For peer review only

For peer review only - http://bmjopen.bmj.com/site/about/guidelines.xhtml

16 REFERENCES

1. Diabetes UK. Facts and Stats [Internet]. diabetes.org.uk. 2015 [cited 2016 Aug 4]. Available from: https://www.diabetes.org.uk/About_us/What-we-say/Statistics/

2. Engoren M, Habib RH, Zacharias A, Schwann TA, Riordan CJ, Durham SJ, et al. The prevalence of elevated hemoglobin A1c in patients undergoing coronary artery bypass surgery. Journal of Cardiothoracic Surgery 2008 3:1. BioMed Central; 2008 Nov 24;3(1):63.

3. Joint British Diabetes Societies for Inpatient Care. Management of adults with diabetes undergoing surgery and elective procedures: Improving standards. 2015 Sep 9;:1–84.

4. Thourani VH, Weintraub WS, Stein B, Gebhart S, Craver JM, Jones EL, et al. Influence of diabetes mellitus on early and late outcome after coronary artery bypass grafting. Ann Thorac Surg. 1999 Apr;67(4):1045–52.

5. Risum Ø, Abdelnoor M, Svennevig JL, Levorstad K, Gullestad L, Bjørnerheim R, et al. Diabetes Mellitus and Morbidity and Mortality Risks after Coronary Artery Bypass Surgery. Scandinavian Journal of Thoracic and Cardiovascular Surgery. 2009 Jul 12;30(2):71–5.

6. Salomon NW, Page US, Okies JE, Stephens J, Krause AH, Bigelow JC. Diabetes mellitus and coronary artery bypass. Short-term risk and long-term prognosis. J Thorac Cardiovasc Surg. 1983 Feb;85(2):264–71.

7. Dhatariya K, Levy N, Kilvert A, Watson B, Cousins D, Flanagan D, et al. NHS Diabetes guideline for the perioperative management of the adult patient with diabetes. Diabet Med. 2012 Apr 1;29(4):420–33.

8. Hirst A, Dutton S, Wu O, Briggs A, Edwards C, Waldenmaier L, et al. A multi-centre retrospective cohort study comparing the efficacy, safety and cost-effectiveness of hysterectomy and uterine artery embolisation for the treatment of symptomatic uterine fibroids. The HOPEFUL study. Health technology assessment (Winchester, England). 2008 Mar 1;12(5):1–248, iii.

9. Rollins KE, Varadhan KK, Dhatariya K, Lobo DN. Systematic review of the impact of HbA_{1c} on outcomes following surgery in patients with diabetes mellitus. Clin Nutr. 2016 Apr;35(2):308–16.

10. National Guideline Centre (UK). Preoperative Tests (Update): Routine Preoperative Tests for Elective Surgery. London: National Institute for Health and Care Excellence (UK); 2016 Apr.

11. Craig P, Dieppe P, Macintyre S, Michie S, Nazareth I, Petticrew M. Developing and evaluating complex interventions: new guidance. Glasgow: Medical Research Council; 2008. 39 p.

12. Foss KT, Kjærgaard J, Stensballe LG, Greisen G. Recruiting to Clinical Trials on the Telephone - a randomized controlled trial. Trials. BioMed Central; 2016 Nov 21;17(1):552.

For peer review only - http://bmjopen.bmj.com/site/about/guidelines.xhtml

13. Hirst JA, Stevens RJ, Farmer AJ. Changes in HbA_{1c} level over a 12-week follow-up in patients with type 2 diabetes following a medication change. Barengo NC, editor. PLoS ONE. Public Library of Science; 2014;9(3):e92458.

14. Cohen O, Dankner R, Chetrit A, Luxenburg O, Langenauer C, Shinfeld A, et al. Multidisciplinary intervention for control of diabetes in patients undergoing coronary artery bypass graft (CABG). Cardiovasc Surg. 2003 Jun;11(3):195–200.

15. Schulz KF, Altman DG, Moher D. BioMed Central | Full text | CONSORT 2010 Statement: updated guidelines for reporting parallel group randomised trials. BMC Med. 2010.

16. Moher D, Hopewell S, Schulz KF, Montori V, Gøtzsche PC, Devereaux PJ, et al. CONSORT 2010 Explanation and Elaboration: updated guidelines for reporting parallel group randomised trials. 2010;340:c869.

17. Older Adults: Standards of Medical Care in Diabetes-2019. American Diabetes Association. Diabetes Care 2019 Jan; 42 (Supplement 1): S139-S147. https://doi.org/10.2337/dc19-S012

18. Rajendran R, Kerry C, Rayman G*, et al* Temporal patterns of hypoglycaemia and burden of sulfonylurea-related hypoglycaemia in UK hospitals: a retrospective multicentre audit of hospitalised patients with diabetes *BMJ Open* 2014;**4:**e005165. doi: 10.1136/bmjopen-2014-005165

19. https://www.nice.org.uk/guidance/ng125/evidence/full-guideline-pdf-6727105694

20. Garner JS, Jarvis WR, Emori TG, Horan TC, Hughes JM. CDC definitions for nosocomial infections, 1988. Am J Infect Control. 1988;16(3):128-40.

21. Biancari, F., Ruggieri, V.G., Perrotti, A., Svenarud, P., Dalén, M., Onorati, F., Faggian, G., Santarpino, G., Maselli, D., Dominici, C. and Nardella, S., 2015. European multicenter study on coronary artery bypass grafting (E-CABG registry): study protocol for a prospective clinical registry and proposal of classification of postoperative complications. *Journal of cardiothoracic surgery*, *10*(1), p.90.

22. Chakrapani Balijepalli, Eric Druyts, Gaye Siliman, Michel Joffres, Kristian Thorlund and Edward J Mills 2017 Hypoglycemia: a review of definitions used in clinical trials evaluating antihyperglycemic drugs for diabetes. Clin Epidemiol, 9: 291–296.

For peer review only - http://bmjopen.bmj.com/site/about/guidelines.xhtml

17 APPENDICES

17.1 APPENDIX 1- PROGRESS GRADING

Table 1 – Actions to be taken depending on progress grade

Grade	Action
Green	Continue trial, keeping an eye on accrual.
Amber	Working with governance committees (TSC, TMG, PPI Committees), seek root cause for under performance. Consider whether these can be mitigated through work with organisations or individuals within the study.
Red	Review the study with governance committees, taking steps as detailed under amber, but also explicitly considering recommending study closure.

For peer review only

For peer review only - http://bmjopen.bmj.com/site/about/guidelines.xhtml

Table 2 - Progress grading time points and criteria

Assessment Point	Green	Amber	Red
After 100 patients have had surgery (50 intervention and 50 control)	HbA_{1c} reduction in intervention group > 5mmol/mol	HbA_{1c} reduction in intervention group < 5mmol/mol	HbA_{1c} reduction in intervention group not consistent with physiological effect
6 complete months after 1st recruiting site has opened	≥ 8 centres have recruited at least one participants<br><br>and<br><br>≥ 50 participants have been recruited<br><br>and<br><br>At least 10 participants have completed their OCTOPuS intervention and have either received surgery, or have had their surgery cancelled or postponed for either clinical or operational reasons.	One or two criteria in green column met	No criteria in green column met
12 complete months after 1st recruiting site has opened	≥ 12 centres have recruited at least one patient<br><br>and<br><br>At least 50 participants have completed their OCTOPuS intervention and have either received surgery, or have had their surgery cancelled or postponed for either clinical or operational reasons.	Only one criterion from green column met	No criteria on green column met
End of month 15 of recruitment	The mean recruitment rate across the trial in months 13, 14, and 15 following the opening of the first recruiting centre is compatible	The mean recruitment rate across the trial in months 13, 14, and 15 following the opening of the first recruiting centre is compatible	The mean recruitment rate across the trial in months 13, 14, and 15 following the opening of the first recruiting centre is not

For peer review only - http://bmjopen.bmj.com/site/about/guidelines.xhtml

	with completing recruitment by the end of month 27 of recruitment	with achieving 75% or more of target recruitment by the end of month 27	compatible with achieving at least 75% of target recruitment by the end of month 27

For peer review only

For peer review only - http://bmjopen.bmj.com/site/about/guidelines.xhtml

18 SUMMARY OF SIGNIFICANT CHANGES TO THE PROTOCOL

Protocol date and version	Summary of significant changes

For peer review only

For peer review only - http://bmjopen.bmj.com/site/about/guidelines.xhtml

Reporting checklist for protocol of a clinical trial.

Based on the SPIRIT guidelines.

Instructions to authors

Complete this checklist by entering the page numbers from your manuscript where readers will find each of the items listed below.

Your article may not currently address all the items on the checklist. Please modify your text to include the missing information. If you are certain that an item does not apply, please write "n/a" and provide a short explanation.

Upload your completed checklist as an extra file when you submit to a journal.

In your methods section, say that you used the SPIRITreporting guidelines, and cite them as:

Chan A-W, Tetzlaff JM, Gøtzsche PC, Altman DG, Mann H, Berlin J, Dickersin K, Hróbjartsson A, Schulz KF, Parulekar WR, Krleža-Jerić K, Laupacis A, Moher D. SPIRIT 2013 Explanation and Elaboration: Guidance for protocols of clinical trials. BMJ. 2013;346:e7586

		Reporting Item	Page Number
**Administrative information**			
Title	#1	Descriptive title identifying the study design, population, interventions, and, if applicable, trial acronym	1
Trial registration	#2a	Trial identifier and registry name. If not yet registered, name of intended registry	2
Trial registration: data set	#2b	All items from the World Health Organization Trial Registration Data Set	n/a
Protocol version	#3	Date and version identifier	n/a
Funding	#4	Sources and types of financial, material, and other support	17
Roles and responsibilities: contributorship	#5a	Names, affiliations, and roles of protocol contributors	1, 17

For peer review only

Roles and responsibilities: sponsor contact information	#5b	Name and contact information for the trial sponsor	16
Roles and responsibilities: sponsor and funder	#5c	Role of study sponsor and funders, if any, in study design; collection, management, analysis, and interpretation of data; writing of the report; and the decision to submit the report for publication, including whether they will have ultimate authority over any of these activities	n/a
Roles and responsibilities: committees	#5d	Composition, roles, and responsibilities of the coordinating centre, steering committee, endpoint adjudication committee, data management team, and other individuals or groups overseeing the trial, if applicable (see Item 21a for data monitoring committee)	14
**Introduction**			
Background and rationale	#6a	Description of research question and justification for undertaking the trial, including summary of relevant studies (published and unpublished) examining benefits and harms for each intervention	4
Background and rationale: choice of comparators	#6b	Explanation for choice of comparators	7-8
Objectives	#7	Specific objectives or hypotheses	5
Trial design	#8	Description of trial design including type of trial (eg, parallel group, crossover, factorial, single group), allocation ratio, and framework (eg, superiority, equivalence, non-inferiority, exploratory)	5
**Methods: Participants, interventions, and outcomes**			
Study setting	#9	Description of study settings (eg, community clinic, academic hospital) and list of countries where data will be collected. Reference to where list of study sites can be obtained	10-11
Eligibility criteria	#10	Inclusion and exclusion criteria for participants. If applicable, eligibility criteria for study centres and individuals who will	5

For peer review only

		perform the interventions (eg, surgeons, psychotherapists)	
Interventions: description	#11a	Interventions for each group with sufficient detail to allow replication, including how and when they will be administered	7-8
Interventions: modifications	#11b	Criteria for discontinuing or modifying allocated interventions for a given trial participant (eg, drug dose change in response to harms, participant request, or improving / worsening disease)	7-8
Interventions: adherance	#11c	Strategies to improve adherence to intervention protocols, and any procedures for monitoring adherence (eg, drug tablet return; laboratory tests)	7-8
Interventions: concomitant care	#11d	Relevant concomitant care and interventions that are permitted or prohibited during the trial	7-8
Outcomes	#12	Primary, secondary, and other outcomes, including the specific measurement variable (eg, systolic blood pressure), analysis metric (eg, change from baseline, final value, time to event), method of aggregation (eg, median, proportion), and time point for each outcome. Explanation of the clinical relevance of chosen efficacy and harm outcomes is strongly recommended	9-10
Participant timeline	#13	Time schedule of enrolment, interventions (including any run-ins and washouts), assessments, and visits for participants. A schematic diagram is highly recommended (see Figure)	7-8
Sample size	#14	Estimated number of participants needed to achieve study objectives and how it was determined, including clinical and statistical assumptions supporting any sample size calculations	10-11
Recruitment	#15	Strategies for achieving adequate participant enrolment to reach target sample size	6
**Methods: Assignment of interventions (for controlled trials)**			
Allocation: sequence generation	#16a	Method of generating the allocation sequence (eg, computer-generated random numbers), and list of any factors for stratification. To reduce predictability of a random sequence, details of any planned restriction (eg, blocking) should be provided in a separate document that is unavailable to those who enrol participants or assign interventions	6

For peer review only

Allocation concealment mechanism	#16b	Mechanism of implementing the allocation sequence (eg, central telephone; sequentially numbered, opaque, sealed envelopes), describing any steps to conceal the sequence until interventions are assigned	n/a
Allocation: implementation	#16c	Who will generate the allocation sequence, who will enrol participants, and who will assign participants to interventions	n/a
Blinding (masking)	#17a	Who will be blinded after assignment to interventions (eg, trial participants, care providers, outcome assessors, data analysts), and how	n/a
Blinding (masking): emergency unblinding	#17b	If blinded, circumstances under which unblinding is permissible, and procedure for revealing a participant's allocated intervention during the trial	n/a
**Methods: Data collection, management, and analysis**			
Data collection plan	#18a	Plans for assessment and collection of outcome, baseline, and other trial data, including any related processes to promote data quality (eg, duplicate measurements, training of assessors) and a description of study instruments (eg, questionnaires, laboratory tests) along with their reliability and validity, if known. Reference to where data collection forms can be found, if not in the protocol	18
Data collection plan: retention	#18b	Plans to promote participant retention and complete follow-up, including list of any outcome data to be collected for participants who discontinue or deviate from intervention protocols	18
Data management	#19	Plans for data entry, coding, security, and storage, including any related processes to promote data quality (eg, double data entry; range checks for data values). Reference to where details of data management procedures can be found, if not in the protocol	18
Statistics: outcomes	#20a	Statistical methods for analysing primary and secondary outcomes. Reference to where other details of the statistical analysis plan can be found, if not in the protocol	12-13
Statistics: additional analyses	#20b	Methods for any additional analyses (eg, subgroup and adjusted analyses)	12-13

For peer review only

Statistics: analysis population and missing data	#20c	Definition of analysis population relating to protocol non-adherence (eg, as randomised analysis), and any statistical methods to handle missing data (eg, multiple imputation)	12-13
**Methods: Monitoring**			
Data monitoring: formal committee	#21a	Composition of data monitoring committee (DMC); summary of its role and reporting structure; statement of whether it is independent from the sponsor and competing interests; and reference to where further details about its charter can be found, if not in the protocol. Alternatively, an explanation of why a DMC is not needed	n/a
Data monitoring: interim analysis	#21b	Description of any interim analyses and stopping guidelines, including who will have access to these interim results and make the final decision to terminate the trial	n/a
Harms	#22	Plans for collecting, assessing, reporting, and managing solicited and spontaneously reported adverse events and other unintended effects of trial interventions or trial conduct	n/a
Auditing	#23	Frequency and procedures for auditing trial conduct, if any, and whether the process will be independent from investigators and the sponsor	n/a
**Ethics and dissemination**			
Research ethics approval	#24	Plans for seeking research ethics committee / institutional review board (REC / IRB) approval	2
Protocol amendments	#25	Plans for communicating important protocol modifications (eg, changes to eligibility criteria, outcomes, analyses) to relevant parties (eg, investigators, REC / IRBs, trial participants, trial registries, journals, regulators)	n/a
Consent or assent	#26a	Who will obtain informed consent or assent from potential trial participants or authorised surrogates, and how (see Item 32)	6
Consent or assent: ancillary studies	#26b	Additional consent provisions for collection and use of participant data and biological specimens in ancillary studies, if applicable	n/a
Confidentiality	#27	How personal information about potential and enrolled participants will be collected, shared, and maintained in order to protect confidentiality before, during, and after the trial	16

For peer review only

Declaration of interests	#28	Financial and other competing interests for principal investigators for the overall trial and each study site	18
Data access	#29	Statement of who will have access to the final trial dataset, and disclosure of contractual agreements that limit such access for investigators	18
Ancillary and post trial care	#30	Provisions, if any, for ancillary and post-trial care, and for compensation to those who suffer harm from trial participation	n/a
Dissemination policy: trial results	#31a	Plans for investigators and sponsor to communicate trial results to participants, healthcare professionals, the public, and other relevant groups (eg, via publication, reporting in results databases, or other data sharing arrangements), including any publication restrictions	16-17
Dissemination policy: authorship	#31b	Authorship eligibility guidelines and any intended use of professional writers	n/a
Dissemination policy: reproducible research	#31c	Plans, if any, for granting public access to the full protocol, participant-level dataset, and statistical code	n/a
**Appendices**			
Informed consent materials	#32	Model consent form and other related documentation given to participants and authorised surrogates	n/a
Biological specimens	#33	Plans for collection, laboratory evaluation, and storage of biological specimens for genetic or molecular analysis in the current trial and for future use in ancillary studies, if applicable	n/a

The SPIRIT Explanation and Elaboration paper is distributed under the terms of the Creative Commons Attribution License CC-BY-NC. This checklist was completed on 02. March 2021 using https://www.goodreports.org/, a tool made by the EQUATOR Network in collaboration with Penelope.ai

For peer review only

BMJ Open

The Optimising Cardiac Surgery ouTcOmes in People with diabeteS (OCTOPuS) randomised-controlled trial to evaluate an out-patient pre-cardiac surgery diabetes management intervention: a study protocol

Journal:	*BMJ Open*
Manuscript ID	bmjopen-2021-050919.R1
Article Type:	Protocol
Date Submitted by the Author:	25-Mar-2021
Complete List of Authors:	Holt, Richard; University of Southampton, Human Development and Health Academic Unit; University Hospital Southampton NHS Foundation Trust, Southampton National Institute for Health Research Biomedical Research Centre<br>Dritsakis, Giorgos; University of Southampton, Clinical Trials Unit<br>Barnard-Kelly, Katharine; Barnard Health – Health Psychology Research<br>Thorne, Kerensa; University of Southampton, Clinical Trial Units<br>Whitehead, Amy<br>Cohen, Lauren; Barnard Health<br>Dixon, Elizabeth; University of Southampton, Clinical Trial Units<br>Patel, Mayank; University Hospital Southampton, Department of Diabetes and Endocrinology<br>Newland-Jones, Philip; University Hospital Southampton, Department of Diabetes and Endocrinology<br>Green, Mark; University Hospital Southampton<br>Partridge, Helen; Royal Bournemouth and Christchurch Hospitals NHS Foundation Trust, Diabetes and Endocrinology<br>Luthra, Suvitesh; University Hospital Southampton, Division of Cardiac Surgery, Wessex Cardiothoracic Centre<br>Ohri, Sunil; University Hospital Southampton, Division of Cardiac Surgery, Wessex Cardiothoracic Centre<br>Salhiyyah, Kareem; Middle East University<br>Lord, Joanne; University of Southampton, Southampton Health Technology Assessments Centre<br>Niven, John; Patient Representative<br>Cook, Andrew; University of Southampton, Wessex Institute
**Primary Subject Heading**:	Diabetes and endocrinology
Secondary Subject Heading:	Cardiovascular medicine
Keywords:	Cardiothoracic surgery < SURGERY, DIABETES & ENDOCRINOLOGY, SURGERY

I, the Submitting Author has the right to grant and does grant on behalf of all authors of the Work (as defined in the below author licence), an exclusive licence and/or a non-exclusive licence for contributions from authors who are: i) UK Crown employees; ii) where BMJ has agreed a CC-BY licence shall apply, and/or iii) in accordance with the terms applicable for US Federal Government officers or employees acting as part of their official duties; on a worldwide, perpetual, irrevocable, royalty-free basis to BMJ Publishing Group Ltd ("BMJ") its licensees and where the relevant Journal is co-owned by BMJ to the co-owners of the Journal, to publish the Work in this journal and any other BMJ products and to exploit all rights, as set out in our licence.

The Submitting Author accepts and understands that any supply made under these terms is made by BMJ to the Submitting Author unless you are acting as an employee on behalf of your employer or a postgraduate student of an affiliated institution which is paying any applicable article publishing charge ("APC") for Open Access articles. Where the Submitting Author wishes to make the Work available on an Open Access basis (and intends to pay the relevant APC), the terms of reuse of such Open Access shall be governed by a Creative Commons licence – details of these licences and which Creative Commons licence will apply to this Work are set out in our licence referred to above.

Other than as permitted in any relevant BMJ Author's Self Archiving Policies, I confirm this Work has not been accepted for publication elsewhere, is not being considered for publication elsewhere and does not duplicate material already published. I confirm all authors consent to publication of this Work and authorise the granting of this licence.

For peer review only

The Optimising Cardiac Surgery ouTcOmes in People with diabeteS (OCTOPuS) randomised-controlled trial to evaluate an out-patient pre-cardiac surgery diabetes management intervention: a study protocol

Richard IG Holt[1-2], Giorgos Dritsakis[3], Katharine Barnard-Kelly[4,5], Kerensa I. Thorne[3], Amy Whitehead[3], Lauren Cohen[4], Elizabeth Dixon[3], Mayank Patel[6], Philip Newland-Jones[6], Mark Green[6], Helen Partridge[7], Suvitesh Luthra[6], Sunil Ohri[6], Kareem Salhiyyah[8], Jo Lord[9], John Niven[10], Andrew Cook[3,6] on behalf of the OCTOPuS study group

[1] Human Development and Health, Faculty of Medicine, University of Southampton, Southampton, UK

[2] Southampton National Institute for Health Research Biomedical Research Centre, University Hospital Southampton NHS Foundation Trust, Southampton, UK

[3] Clinical Trials Unit, Faculty of Medicine, University of Southampton, Southampton, UK

[4] Barnard Health – Health Psychology Research, Fareham, UK

[5] Faculty of Health & Social Science, Bournemouth University

[6] University Hospital Southampton, Southampton, UK

[7] Royal Bournemouth and Christchurch Hospitals NHS Foundation Trust, Bournemouth, UK

[8] Middle East University, Amman, Jordan

[9] Southampton Health Technology Assessments Centre, University of Southampton, UK

[10] Patient and public representative

Name and email address of corresponding author: Professor Richard Holt

R.I.G.Holt@soton.ac.uk

For peer review only - http://bmjopen.bmj.com/site/about/guidelines.xhtml

Abstract

Introduction: Cardiothoracic surgical outcomes are poorer in people with diabetes compared with those without diabetes. There are two important uncertainties in the management of people with diabetes undergoing major surgery; 1) how to improve diabetes management in the weeks leading up to an elective procedure, and 2) whether that improved management leads to better post-operative outcomes. We previously demonstrated the feasibility of delivering the OCTOPuS intervention, an outpatient intervention delivered by diabetes healthcare professionals for people with sub-optimally managed diabetes over 8-12 weeks before elective cardiac surgery. The present study will assess the clinical and cost-effectiveness of the intervention in cardiothoracic centres across the UK.

Methods and Analysis: A multicentre, parallel group, single-blinded 1:1 individually randomised trial comparing time from surgery until clinically fit for discharge in adults with sub-optimally managed type 1 or type 2 diabetes undergoing elective surgery between the OCTOPuS intervention and usual care (primary endpoint). Secondary endpoints will include: actual time from surgery to discharge from hospital; days alive and either out of hospital or judged as clinically fit for discharge; mortality; time on ITU/ventilator; infections; acute myocardial infarction; change in weight; effect on post-operative renal function and incidence of acute kidney injury; change in HbA_{1c}; frequency and severity of self-reported hypoglycaemia; operations permanently cancelled for sub-optimal glycaemic levels; cost effectiveness; psychosocial questionnaires. The target sample size will be 426 recruited across approximately 15 sites. The primary analysis will be conducted on an intention to treat population. A two-sided p-value of 0.05 or less will be used to declare statistical significance for all analyses and results will be presented with 95% confidence intervals.

Ethics and dissemination. The trial was approved by the South Central - Hampshire A Research Ethics Committee (20/SC/0271). Results will be disseminated through conferences, scientific journals, newsletters, magazines and social media.

Trial Registration: ISRCTN10170306

Keywords: diabetes, cardiothoracic surgery, OCTOPuS intervention, randomised-clinical trial, post-operative outcomes

For peer review only - http://bmjopen.bmj.com/site/about/guidelines.xhtml

Word count: 3683

Article summary

Strengths and limitations of the study

- The OCTOPuS intervention was developed according to the MRC Framework for complex interventions and successfully piloted in a single cardiothoracic surgical centre.
- This is the first trial to assess whether early contact with a specialist diabetes team in the weeks leading up to surgery improves cardiothoracic surgical outcomes and reduces the excess morbidity and mortality experienced by people with diabetes.
- Hospital length of stay is an important clinical and economic measure of the success of surgery
- The sample size and number of sites will mean that the results are sufficiently generalisable to the remaining cardiothoracic centres across the UK
- The start of the study will likely be delayed by Covid-19 because of the effect of the epidemic on elective surgery.

w only

For peer review only - http://bmjopen.bmj.com/site/about/guidelines.xhtml

1 INTRODUCTION

The prevalence of cardiovascular disease is increased approximately 2-fold in people with diabetes after adjustment for other cardiovascular risk factors.[1] It affects approximately a third of all people with type 2 diabetes and contributes to over 50% of deaths.[2] As coronary heart disease in people with diabetes tends to be more diffuse affecting multiple vessels, coronary artery bypass grafting is often the preferred method for re-vascularisation. Approximately 30-40% of all people undergoing open cardiac surgery have diabetes.[3]

Surgical outcomes are worse in people with diabetes with an up to three-fold higher risk of post-operative complications which include poor healing, wound complications, and renal dysfunction.[4,5] These complications are associated with longer hospital stay and higher re-admission rates. The reasons underlying the poorer outcomes include hyperglycaemia, dyslipidaemia and obesity. Although national and international groups have published detailed guidelines to improve surgical outcomes in people with diabetes, many people with diabetes are poorly prepared for surgery.[6-8] In the E-CABG study, 54% of people with type 2 diabetes treated with non-insulin medications and 67% of those with insulin-treated diabetes had an HbA_{1c} above 53 mmol/mol (7.0%) prior to cardiac surgery.[5]

There are two important uncertainties in the management of people with sub-optimally managed diabetes undergoing major surgery; 1) how to improve diabetes management in the weeks leading to elective surgery, and 2) whether that improved management is reflected in better surgical outcomes. To address these gaps, the overarching aim of the Optimising Cardiac Surgery ouTcOmes in People with diabeteS (OCTOPuS) project is to develop and test whether a pre-operative out-patient intervention to improve diabetes management improves cardiac surgical outcomes.

The development of the intervention is described in detail elsewhere (Holt et al. Developing an intervention to optimise the outcome of cardiac surgery in people with diabetes: the OCTOPuS pilot study. Under review). In summary, the prototype OCTOPuS intervention was based on a nurse-led outpatient intervention that has been used in Royal Bournemouth Hospital for 7 years and incorporated the findings of two rapid literature reviews. A feasibility study conducted in 17 people with diabetes undergoing cardiothoracic surgery at University Hospital Southampton showed that it is possible to develop a clinical pathway to deliver the

For peer review only - http://bmjopen.bmj.com/site/about/guidelines.xhtml

OCTOPuS intervention to improve glycaemic management prior to admission that was acceptable for people with diabetes and clinicians.

The present study will be a multicentre randomised controlled trial (RCT) in cardiothoracic centres across the UK to assess the clinical and cost-effectiveness of the intervention.

2 METHODS AND ANALYSIS

2.1 STUDY DESIGN

OCTOPuS is a multicentre, parallel group, single blind, individually randomised RCT incorporating a pre-planned futility analysis. It will compare time from surgery until an individual is clinically fit for discharge in adults with sub-optimally managed type 1 diabetes or type 2 diabetes undergoing elective cardiothoracic surgery between the OCTOPuS intervention and usual care. The provisional planned trial recruitment dates are: 01/09/2021 – 31/08/2023. These are contingent on the re-opening of elective cardiothoracic surgery and research capacity following the latest national Covid-19 lockdown.

2.2 ELIGIBILITY

2.2.1 Inclusion Criteria

1. Aged ≥ 18 years old with type 1 diabetes or type 2 diabetes
2. Sub-optimally managed diabetes defined as an HbA_{1c} >53 mmol/mol (7%) for those ≤75 years old and an HbA_{1c} >64 mmol/mol (8%) for those >75 years old. The higher HbA_{1c} criterion for older people is to minimise the risk of iatrogenic hypoglycaemia[9]. This will be measured using a near patient test at the cardiothoracic surgery outpatient appointment where the decision to proceed to surgery is made.
3. Awaiting elective open-heart cardiac surgery
4. Anticipated delay before surgery of at least 2 months.
5. Surgery will take place at a hospital participating in the trial
6. Ability to give informed consent.
7. Ability to interact with the study documentation and processes

For peer review only - http://bmjopen.bmj.com/site/about/guidelines.xhtml

2.2.2 *Exclusion Criteria*

1. Active malignancy, where the malignancy is currently being treated by chemotherapy, surgery or radiotherapy or is likely to cause death within 6 months
2. Pregnancy
3. Previous cardiac surgery
4. Known haemoglobinopathies that affect the measurement of HbA_{1c}
5. Other illnesses or conditions that would preclude engagement with the OCTOPuS intervention
6. Surgery taking place outside the participating hospitals, e.g. at a private hospital

2.3 RECRUITMENT

2.3.1 *Screening & consent*

Outpatient cardiac surgery appointment clinic lists will be scrutinised ahead of appointments and an information sheet explaining the trial will be sent by post or email as appropriate to people who appear eligible (including contact details to opt out if the person does not want further contact about the trial). Before the outpatient appointment, a researcher will contact the prospective participant to discuss the study at least 24 hours before the appointment allowing time for reflection and discussion. This will permit eligible individuals to be randomised immediately after the outpatient appointment, and where possible receive their OCTOPuS consultation, on the same day. The treating surgeon will remind eligible patients about the trial if a decision to proceed to surgery is made. If the person wishes to participate, they will have the opportunity to discuss the study face-to-face with a research nurse before they give written consent. Final eligibility criteria will be checked prior to recruitment. Patients whose medical records cannot be accessed prior to the appointment to determine eligibility (e.g. patients from another hospital), will be given information about the study on the day of the appointment and will be offered the opportunity to attend another day to discuss participation.

2.3.2 *Randomisation*

Participants will be individually randomised in a 1:1 ratio, stratified by centre and age (≤75 years old and >75 years old), using permuted blocks. The study flow is illustrated in Figure 1.

For peer review only - http://bmjopen.bmj.com/site/about/guidelines.xhtml

[Figure 1 here]

2.4 STUDY PROCEDURES

2.4.1 Baseline measurements

After randomisation, the following data will be collected on participants in both arms: medical history and examination; vital signs; biochemistry; self-reported episodes of hypoglycaemia

2.4.2 The OCTOPuS intervention

2.4.2.1 Initial consultation

Participants randomised to receive the OCTOPuS intervention will have an initial consultation with an OCTOPuS practitioner, who may be a doctor, nurse, pharmacist, or other appropriately trained healthcare professional. In this consultation the participant's diabetes management will be discussed, as well as the likely benefits of improved glycaemic management prior to surgery. The practitioner and participant will agree actions, tailored to the individual needs and ability, including:

- A graded exercise regimen. This may be completely self-delivered, or alternatively by joining a local appropriate exercise scheme – such as a 'health walk'.
- Dietary advice, supplemented by a consultation with a dietitian if needed
- Medication review, which may lead to the introduction of insulin or other diabetes medications for people with type 2 diabetes.
- Specific advice about managing expectations, understanding facilitators to achieve change and overcoming barriers to improve medical and psychosocial outcomes

The exact process and the treatment options are set out in the OCTOPuS intervention manual (Supplementary Material 1).

For peer review only - http://bmjopen.bmj.com/site/about/guidelines.xhtml

2.4.2.2 Support calls

After the initial consultation, participants will receive regular review with the OCTOPuS practitioner, usually by telephone, at least once a fortnight until the participant's diabetes management goals have been reached and no further changes are needed. After this, the frequency of the calls can be reduced at the discretion of the OCTOPuS practitioner and participant to a minimum of every 6 weeks. This contact will be an opportunity to offer encouragement and support and address any issues which have arisen for the participant. One more support contact will be made 1-3 weeks after discharge to ensure the continuity of diabetes management beyond surgery. Where necessary the OCTOPuS practitioner will liaise with local services, e.g. the participant's GP or a dietitian, to facilitate delivery.

2.4.3 Control arm

Participants in the control arm will receive usual care in the cardiac surgery centre attended by the individual. This is likely to contain standardised brief advice from the surgeon to pay attention to their diabetes prior to surgery. Some people may act on this advice, either on their own or in conjunction with their GP. The study will document 'usual care' at all recruiting centres and explore with participants in the control arm as part of the qualitative work what actions were taken in response to advice received.

2.4.4 Follow up visits

After participants are randomised to either the intervention or control arm, data will be collected from them at the following timepoints: pre-surgery; discharge; 7 days post-surgery; 30 days post-surgery; and at their next routine diabetes care visit between 90 and 180 days post-surgery. In addition to the baseline measures, information about the surgery, infections and surgical complications, mortality and adverse events will be collected (***TABLES***
***Table* 1**). Pre-surgery, surgery and discharge data will be collected in hospital. After discharge, data will be collected remotely, e.g. over the phone, by post, through in-patient note review or where possible using adult cardiac surgery databases (e.g. the SCTS National Adult Cardiac Surgery Audit).

For peer review only

For peer review only - http://bmjopen.bmj.com/site/about/guidelines.xhtml

[Table 1 here]

2.5 ENDPOINTS

2.5.1 Primary endpoint

Time from surgery until clinically fit for discharge, as judged by the surgical team. Teams will be blinded to pre-hospital diabetes management allocation. This primary outcome was chosen because reduced time in hospital (though not at the expense of safety) is valued by people with diabetes, clinicians, and commissioners.

2.5.2 Secondary endpoints

- Time from surgery to actual discharge from hospital – this recognises that discharge can be delayed for non-clinical reasons
- Days alive between surgery and either out of hospital or judged as clinically fit for discharge
- Pre-operative mortality; 30 day mortality; 90 day mortality
- Time on ITU
- Time on a ventilator
- Sternal Wound Infections, defined according to the NICE guidance and the CDC criteria[10,11]
- Leg wound infections, in those who provide donor veins; graded according to the Centers for Disease Control and Prevention definitions of surgical site infections.[12]
- Chest infections, defined as a change in typical chest symptoms (cough, increase respiratory rate, shortness of breath) in conjunction with a fever or inflammatory markers.
- Urinary tract infections, defined as "clinically-diagnosed and treated, whether or not results from a urine culture are available''
- Acute Coronary Syndrome[12]
- Change in weight between randomisation and surgery

For peer review only - http://bmjopen.bmj.com/site/about/guidelines.xhtml

- Effect on post-operative renal function and incidence of acute kidney injury as assessed by measurement of serum creatinine and calculation of estimated glomerular filtration rates[12]
- HbA_{1c} immediately preoperative, and at between 90 and 180 days post operation.
- Change in HbA_{1c} between baseline and immediately preoperative, and change from preoperative to between 90 and 180 days post operation
- Operations cancelled for sub-optimal glycaemic management
- Frequency and severity of self-reported overall, minor, severe and nocturnal hypoglycaemia assessed at Baseline, during the Support Contact and Pre-surgery.[13]
- EQ-5D at baseline, 7, 30 and 90 days post-surgery.
- Qualitative interviews and psychosocial questionnaires at baseline and 90 days post-surgery to explore participants' experiences and perceived benefits of the intervention and any changes to their diabetes self-management.
- Cost effectiveness of intervention, including: use of NHS lifestyle improvement programs and diabetes services; use of medication, time spent by practitioners for training, delivering the intervention and liaising with local services; HbA_{1c} point-of-care and blood glucose monitoring costs

2.6 SAMPLE SIZE

2.6.1 Futility Assessment – Physiological Effect of Intervention

To demonstrate that a physiological response is plausible we need to show an HbA_{1c} reduction of 5 mmol/mol in the intervention group pre-surgery compared to baseline. Previous experience shows the mean initial HbA_{1c} in our study population is approximately 72 mmol/mol, with a standard deviation of 15 mmol/mol.[14,15] For an expected change in HbA_{1c} from baseline of 5 mmol/mol in the intervention group, and assuming a correlation of 50% between baseline and pre-surgery, a sample size in the intervention group of 50 participants would allow a margin of error of 4.16 below the mean for a 95% confidence interval (CI) and would, therefore, allow us to exclude a difference of zero if the treatment difference of 5 was observed.

For peer review only - http://bmjopen.bmj.com/site/about/guidelines.xhtml

2.6.2 Intervention effectiveness – clinical outcomes

The primary outcome is the time from surgery to when the responsible consultant considers the participant clinically fit for discharge. We will not consider the actual discharge date in the primary analysis, as currently many elective cardiothoracic surgical patients remain in hospital longer than clinically indicated due to their social situation. Discussions with clinicians and commissioners suggest that a mean improvement of half a day would be clinically worthwhile.

The current mean duration post-surgery until clinically fit for discharge is 7 days, with a standard deviation of 1.5 days. To demonstrate an improvement of 0.5 days with 90% power and 5% significance with 1:1 randomisation between intervention and control arms would require a total of 382 participants (nQuery v7.0). We will allow for a 5% loss to follow-up, and 5% for deaths post randomisation inflating the final target sample size to 426 participants. Participants will be recruited across approximately 15 UK cardiothoracic centres.

2.7 INTERIM ANALYSIS

Futility will be assessed, and the trial could be stopped early for one of two main reasons:

2.7.1 Recruitment and Delivery

There are several threats to recruitment and delivery of this trial:

- Being unable to recruit and initiate sufficient centres.
- Centres being unable to recruit sufficient participants
- Centres being unable to deliver the OCTOPuS intervention

Therefore throughout the trial, we will review progress against criteria at three time points, grading trial progress as red, amber, or green each time (Supplementary material 2).

For peer review only - http://bmjopen.bmj.com/site/about/guidelines.xhtml

2.7.2 Physiological Effect of Intervention

It is believed that the OCTOPuS intervention will have its clinically relevant effects through improvement of clinical measures, including change in body weight, exercise, lipid profile and blood pressure. However, the main target of the intervention is to improve glycaemic management; if no physiological effect can be demonstrated on glycaemic measures, continuation of the trial would be considered futile. After the first 100 participants have had their surgery, we will assess the effect of the intervention on pre-operative HbA_{1c}. If there is no discernible effect (defined as a change of HbA_{1c} of <5 mmol/mol) we will ask the trial steering committee to review the trial's viability.

2.8 STATISTICAL ANALYSIS

Baseline participant demographics and characteristics will be summarised between the two arms[16]. The primary analysis will be conducted using ANCOVA adjusted for randomisation stratification factors on an intention to treat population. Continuous data will be presented as means and standard deviations and analysed using ANCOVA (or presented as medians and ranges and analysed using Mann-Whitney U tests if data are skewed). Binary data will be reported in terms of odds ratios and analysed using logistic regression modelling. Analysis of time-to-event outcomes will include presenting Kaplan-Meier graphs by arm and analysed using Cox proportional hazards regression (or competing risk regression as discussed below). A two-sided p-value of 0.05 or less will be used to declare statistical significance for all analyses and results will be presented with 95% confidence intervals. Subgroups will be investigated, including those with HbA_{1c} above or below 69 mmol/mol at presentation; type of diabetes; age above or below 75 years. The cut-off of 69 mmol/mol has been chosen as the level above which the Joint British Diabetes Societies recommend specific action to improve pre-operative glycaemic management. The cut-off for age has been chosen to reflect the different HbA_{1c} entry criteria for those above and below 75 years.

It is possible that a small proportion of participants will receive the intervention/usual care but will not actually undergo the planned surgery due to death, or clinically directed surgery cancellation. A small proportion may also undergo urgent revascularisation due to myocardial infarction after they have received their allocated treatment. A further group may undergo

For peer review only - http://bmjopen.bmj.com/site/about/guidelines.xhtml

surgery but die before they are fit for discharge and thus not meet the primary endpoint. It is expected that these events will occur in fewer than 5% of participants. These individuals will be excluded from the primary analysis but the prevalence of each of these outcomes will be monitored and recorded by treatment arm separately to assess if there is an excess of any of these outcomes in either group. A sensitivity analysis will be considered, looking at a competing risks model, where these outcomes and functional recovery are competing risks. This sensitivity analysis will also be performed if the total prevalence of these events exceeds 5%.

2.9 ECONOMIC EVALUATION

Quality adjusted life years (QALYs) will be estimated from EQ-5D-5L and mortality data, using the area-under-the-curve method. Similarly, costs will be estimated at the patient level. Mean between-group differences in QALYs and costs will be estimated using a regression-based approach, including adjustment for baseline covariates and interaction terms for pre-defined sub-groups, and allowing for clustering at hospital and/or practitioner level. Results will be presented as an Incremental Cost-Effectiveness Ratio (ICER) if appropriate. Non-parametric bootstrapping will be used to estimate confidence intervals around estimated cost differences and ICERs.

A simple modelling approach will also be used to estimate the costs and health impacts of surgical complications over a lifetime horizon. This extrapolation is necessary to reflect any mortality or lasting quality of life decrement associated with surgical complications. There will be no attempt to estimate the long-term impact of improved diabetes management related to the intervention, as it will be difficult to predict the duration over which any improvements will be maintained. This is likely to be a conservative assumption that will under-estimate the QALY gain and cost-effectiveness of intervention if it proves effective. Model parameters will be estimated from the trial and from other published sources. Long-term resource use, mortality and utility decrements associated with key surgical complications, will be identified by systematic review of HTAs, NICE guidelines and published literature.

For peer review only - http://bmjopen.bmj.com/site/about/guidelines.xhtml

For peer review only

2.10 QUALITATIVE & PSYCHOSOCIAL EVALUATION

2.10.1 Interviews

Fifty participants receiving the intervention will be recruited across all participating sites balanced for age, gender, HbA_{1c}, socioeconomic status and ethnicity. Baseline interviews will take place within 2 weeks of participants' commencing the intervention and follow-up interviews will be conducted with the same participants at 90 days post-surgery. Key personnel involved in the delivery will be interviewed once around 12 months after the start of trial in their centre.

Interview data analysis will include: (a) comparisons between participants' baseline and follow-up interviews to identify changes in their perceptions, experiences and diabetes self-management practices over time, and the reasons for these; (b) comparison of participant and health professional accounts to identify similarities and differences in their understandings and any impact on diabetes self-management practices; (c) cross-comparison of participants' accounts to identify common issues and experiences as well differences in diabetes self-management practices between subgroups of participants (e.g. men versus women, participants of different ages etc.), and the reasons for these.

2.10.2 Psychosocial Questionnaires

The following questionnaires will be completed by participants at baseline and at 3 months post-surgery:

- Diabetes Empowerment Scale (short form): an 8-item questionnaire assessing diabetes-related psychosocial self-efficacy.
- PAID5: a 5-item self-reported measure of diabetes related distress with high internal consistency.
- Patient Health Questionnaire (PHQ-2): ultra-brief depression screener, variant of PHQ-9. It is not used to establish a final diagnosis or to monitor depression severity but rather to screen for depression as a 'first step' approach.

For peer review only - http://bmjopen.bmj.com/site/about/guidelines.xhtml

- Brief Illness Perception Questionnaire (B-IPQ): an 8-item measure assessing cognitive illness representations, emotional representations, illness comprehensibility and perceived causal factors for illness.
- Summary of Diabetes Self-Care Activities scale (SDSCA): a 15-item self-report questionnaire of diabetes self-management that includes items assessing the following aspects of the diabetes regimen: general diet, specific diet, exercise, blood-glucose testing, foot care, and smoking.

The analysis of the questionnaire responses will aim to answer the following questions:

1. What effect does baseline score (categorised as high/low etc. as appropriate) have on study outcomes, i.e. days until considered fit for surgery?
2. What effect does the study intervention have on change in score assessed as a continuous variable from baseline to 90 days post-surgery?
3. Does the treatment work better or less well in people depending on their baseline score (categorised)?

3 SAFETY

Standard definitions and reporting procedures of adverse events, serious adverse events (SAEs), seriousness will be used (Supplementary material 3). For the purposes of this study, the following saes will not require reporting to Southampton Clinical Trials Unit:

- Hospitalisations for elective treatment of a pre-existing condition

Also, the following SAEs will not require reporting if they occur between 'Surgery' and 'Discharge':

- Arrhythmia, including atrial fibrillation
- Immediate postoperative surgical bleeding
- Pneumonia

Expectedness assessments are made against the list of expected events below:

- Minor musculoskeletal aches and pains
- Myocardial infarction
- Respiratory tract infection

For peer review only - http://bmjopen.bmj.com/site/about/guidelines.xhtml

4 PATIENT AND PUBLIC INVOLVEMENT

The trial has been developed in collaboration with the study patient and public involvement advisory group and local branch of Diabetes UK. The trial includes two patient representatives as a member of the Trial Steering Committee (TSC) and a member of the Trial Management group. Both individuals have been involved in the development of this protocol and have attended meetings regularly. To date, they have had an active role in assessing the study progress to date and both will be involved in resolving any issues that may arise.

For peer review only

5 ETHICS

Ethics approval was obtained by the South Central - Hampshire A Research Ethics Committee on 25 August 2020 (20/SC/0271). University Hospital Southampton NHS Foundation Trust will sponsor the study (RHM MED1718). The study is funded by the National Institute of Health Research *Health Technology Assessment* (*HTA*) Programme (16/25/12). The day-to-day management of the trial will be co-ordinated through the Southampton Clinical Trials Unit and oversight will be maintained by the Trial Steering Committee. The study will be conducted in accordance with WMA Declaration of Helsinki and as revised and recognised by governing laws and EU Directives.

All participants may withdraw at any time without providing a reason. Investigators will explain the value of remaining in study follow-up and allowing these data to be used for trial purposes. Where possible, those who have withdrawn from study treatment should remain in follow-up as per the trial schedule. If participants additionally withdraw consent for this, they will revert to standard clinical care. The study team will continue to collect standard follow-up data unless the participant explicitly states otherwise.

6 DISSEMINATION

Results will be disseminated through national and international conferences, scientific journals, newsletters, magazines and social media. Target audiences include diabetes

For peer review only - http://bmjopen.bmj.com/site/about/guidelines.xhtml

specialist teams, cardiac surgeons, primary care team and medical professionals or scientists overall as well as people with diabetes. This study addresses an important clinical question and is the first to assess whether early contact with a specialist diabetes team in the weeks leading up to surgery improves cardiothoracic surgical outcomes and reduces the excess morbidity and mortality experienced by people with diabetes. We further believe that the sample size and number of sites will mean that the results are sufficiently generalisable to broader cardiothoracic practice across the UK and internationally.

Author statement

RIGH is the chief investigator of the OCTOPuS study. He wrote the first draft of the protocol and will act as guarantor for the study. GD and ED will be the trial managers; GD further had significant contribution in editing and finalising the paper. KBK and LC wrote the qualitative sections of the protocol. KIT and AW wrote the statistical and data analysis sections of the protocol; AW was also involved in the original grant application and the design of the study. MP, PNJ and MG were involved in the design of the study. MP will lead the 'clinical' OCTOPuS intervention team and PNJ and MG will be members of the team. HP was involved in the design of the study and intervention. SL and SO were involved in the design of the study from the cardiothoracic perspective. JL wrote the health economics aspects of the protocol. JN is a patient representative on the trial management team and supported the development of the protocol. AC is associate director of the Clinical Trials Unit and was involved in the design of the study. KS was involved in the original grant application and the design of the study. All authors have critically revised the paper for intellectual content and approved the final draft. All authors agree to be accountable for all aspects of the work by ensuring that questions related to the accuracy or integrity of any part of the work are appropriately investigated and resolved.

Funding

The OCTOPuS project is funded by the UK National Institute for Health Research (NIHR) [Health Technology Assessment programme (grant number 16/25/12)]. The views expressed are those of the authors and not necessarily those of the NIHR or the Department of Health and Social Care.

For peer review only - http://bmjopen.bmj.com/site/about/guidelines.xhtml

For peer review only

Conflicts of interest

The authors have no competing interest to report.

Data statement

Participant data will be entered remotely at site and retained in accordance with current Data Protection Regulations. The PI will be responsible for ensuring the accuracy, completeness, and timeliness of the data entered. The participant data are pseudo-anonymised and the site retains a participant identification code list which is only available to site staff. Trained personnel with specific roles assigned will be granted access to the electronic case report forms (eCRF).

SCTU operate a transparent data sharing request process for results that are available in the public domain. As a minimum, anonymous data will be available for request from three months after publication of an article, to researchers who provide a completed Data Sharing request form that describes a methodologically sound proposal, for the purpose of the approved proposal and if appropriate a signed Data Sharing Agreement. Data will be shared once all parties have signed relevant data sharing documentation.

For peer review only

For peer review only - http://bmjopen.bmj.com/site/about/guidelines.xhtml

7 REFERENCES

1. Rao Kondapally Seshasai S, Kaptoge S, Thompson A, et al. Diabetes mellitus, fasting glucose, and risk of cause-specific death. *N Engl J Med* 2011;364(9):829-41. doi: 10.1056/NEJMoa1008862 [published Online First: 2011/03/04]
2. Einarson TR, Acs A, Ludwig C, et al. Prevalence of cardiovascular disease in type 2 diabetes: a systematic literature review of scientific evidence from across the world in 2007-2017. *Cardiovasc Diabetol* 2018;17(1):83. doi: 10.1186/s12933-018-0728-6 [published Online First: 2018/06/10]
3. Raza S, Blackstone EH, Sabik JF, 3rd. The diabetes epidemic and its effect on cardiac surgery practice. *J Thorac Cardiovasc Surg* 2015;150(4):783-4. doi: 10.1016/j.jtcvs.2015.07.037 [published Online First: 2015/08/25]
4. Biancari F, Giordano S. Glycated Hemoglobin and the Risk of Sternal Wound Infection After Adult Cardiac Surgery: A Systematic Review and Meta-Analysis. *Semin Thorac Cardiovasc Surg* 2019;31(3):465-67. doi: 10.1053/j.semtcvs.2019.02.029 [published Online First: 2019/03/10]
5. Nicolini F, Santarpino G, Gatti G, et al. Utility of glycated hemoglobin screening in patients undergoing elective coronary artery surgery: Prospective, cohort study from the E-CABG registry. *Int J Surg* 2018;53:354-59. doi: 10.1016/j.ijsu.2018.04.021 [published Online First: 2018/04/18]
6. Dhatariya K, Levy N, Kilvert A, et al. NHS Diabetes guideline for the perioperative management of the adult patient with diabetes. *Diabet Med* 2012;29(4):420-33. doi: 10.1111/j.1464-5491.2012.03582.x [published Online First: 2012/02/01]
7. Lazar HL, McDonnell M, Chipkin SR, et al. The Society of Thoracic Surgeons practice guideline series: Blood glucose management during adult cardiac surgery. *Ann Thorac Surg* 2009;87(2):663-9. doi: 10.1016/j.athoracsur.2008.11.011 [published Online First: 2009/01/24]
8. O'Neill F, Carter E, Pink N, et al. Routine preoperative tests for elective surgery: summary of updated NICE guidance. *BMJ* 2016;354:i3292. doi: 10.1136/bmj.i3292 [published Online First: 2016/07/16]
9. Older Adults: Standards of Medical Care in Diabetes-2019. American Diabetes Association. Diabetes Care 2019 Jan; 42 (Supplement 1): S139-S147. https://doi.org/10.2337/dc19-S012

For peer review only - http://bmjopen.bmj.com/site/about/guidelines.xhtml

10. https://www.nice.org.uk/guidance/ng125/evidence/full-guideline-pdf-6727105694

11. Garner JS, Jarvis WR, Emori TG, Horan TC, Hughes JM. CDC definitions for nosocomial infections, 1988. Am J Infect Control. 1988;16(3):128-40.

12. Biancari, F., Ruggieri, V.G., Perrotti, A., Svenarud, P., Dalén, M., Onorati, F., Faggian, G., Santarpino, G., Maselli, D., Dominici, C. and Nardella, S., 2015. European multicenter study on coronary artery bypass grafting (E-CABG registry): study protocol for a prospective clinical registry and proposal of classification of postoperative complications. *Journal of cardiothoracic surgery*, *10*(1), p.90.

13. Chakrapani Balijepalli, Eric Druyts, Gaye Siliman, Michel Joffres, Kristian Thorlund and Edward J Mills 2017 Hypoglycemia: a review of definitions used in clinical trials evaluating antihyperglycemic drugs for diabetes. Clin Epidemiol, 9: 291–296.

14. Hirst JA, Stevens RJ, Farmer AJ. Changes in HbA_{1c} level over a 12-week follow-up in patients with type 2 diabetes following a medication change. Barengo NC, editor. PLoS ONE. Public Library of Science; 2014;9(3):e92458.

15. Cohen O, Dankner R, Chetrit A, Luxenburg O, Langenauer C, Shinfeld A, et al. Multidisciplinary intervention for control of diabetes in patients undergoing coronary artery bypass graft (CABG). Cardiovasc Surg. 2003 Jun;11(3):195–200.

16. Moher D, Hopewell S, Schulz KF, Montori V, Gøtzsche PC, Devereaux PJ, et al. CONSORT 2010 Explanation and Elaboration: updated guidelines for reporting parallel group randomised trials. 2010;340:c869.

For peer review only

For peer review only - http://bmjopen.bmj.com/site/about/guidelines.xhtml

8 TABLES

Table 1. Summary of data collection during the OCTOPuS study at various time-points

Time-point	Screening	Consent	Baseline	Intervention	Support calls (every 2-6 weeks post-op)	Pre-Surgery assessments	Surgery	Discharge	Surgery +7 days	Post-discharge support call	Surgery +30 days	Surgery +90 days	End of study
Notes review	X												
Informed Consent		X											
Eligibility evaluation (incl. pregnancy test where appropriate)	X	X	X										
Medical History (incl. smoking status, diabetes and current medications)			X			X							
Physical Exam (incl. height, weight and			X (height will only be recorded			X							

peer review only

For peer review only - http://bmjopen.bmj.com/site/about/guidelines.xhtml

Time-point	Screening	Consent	Baseline	Intervention	Support calls (every 2-6 weeks post-op)	Pre-Surgery assessments	Surgery	Discharge	Surgery +7 days	Post-discharge support call	Surgery +30 days	Surgery +90 days	End of study
waist circumference)			at Baseline)										
Vital Signs (incl. BP)			X			X							
Biochemistry (incl HbA_{1c}, blood glucose and renal function)			X			X		X (only serum creatinine and renal function to capture acute kidney failure)				X (between 90-180 days post-op)	
Hypoglycaemia			X		X	X							
Infections and surgical complications								x			x	X	
Mortality						x					x	x	x
Intervention				X									

For peer review only

For peer review only - http://bmjopen.bmj.com/site/about/guidelines.xhtml

Time-point	Screening	Consent	Baseline	Intervention	Support calls (every 2-6 weeks post-op)	Pre-Surgery assessments	Surgery	Discharge	Surgery +7 days	Post-discharge support call	Surgery +30 days	Surgery +90 days	End of study
Intervention support phone call (incl. review of diary card and components of intervention utilised)					X					x			
Practitioner time (cost-effectiveness)			X	X	X					X			
NHS resource use questions (cost-effectiveness)						X							
Surgery (inc. time on ventilator/ITU)							X						
Blinded assessment								X					

For peer review only

For peer review only - http://bmjopen.bmj.com/site/about/guidelines.xhtml

Time-point	Screening	Consent	Baseline	Intervention	Support calls (every 2-6 weeks post-op)	Pre-Surgery assessments	Surgery	Discharge	Surgery +7 days	Post-discharge support call	Surgery +30 days	Surgery +90 days	End of study
Adverse Events			X	X	X	X			X	X	X	X	X
EQ-5D 5L			X						X		X	X	
Participant qualitative interview			X									X	
Psychosocial questionnaires			X									X	

For peer review only

For peer review only - http://bmjopen.bmj.com/site/about/guidelines.xhtml

9 FIGURE LEGENDS

Figure 1. OCTOPuS study flowchart.

For peer review only

For peer review only - http://bmjopen.bmj.com/site/about/guidelines.xhtml

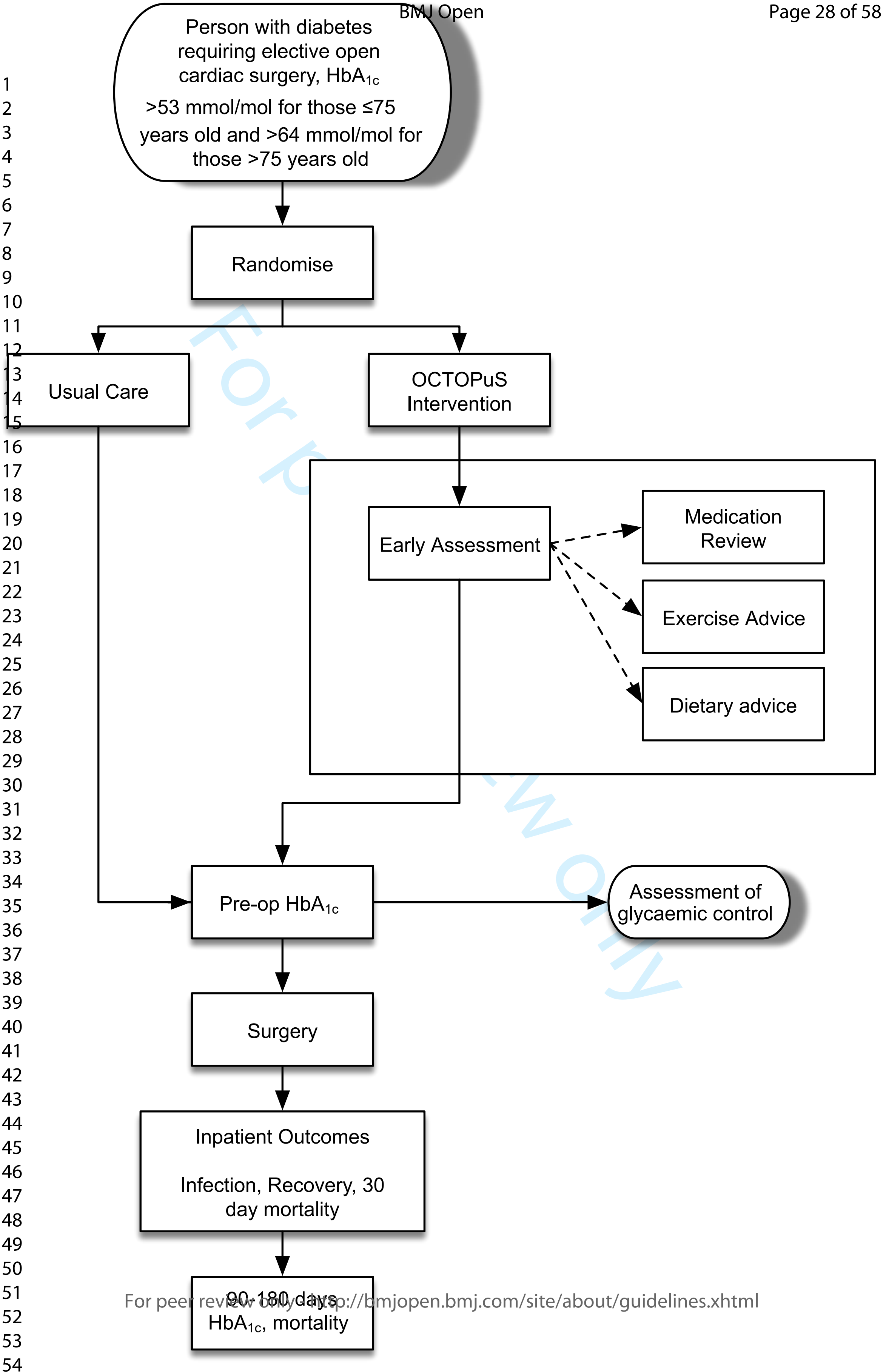
Person with diabetes requiring elective open cardiac surgery, HbA1c >53 mmol/mol for those ≤75 years old and >64 mmol/mol for those >75 years old
Randomise
Usual Care
OCTOPuS Intervention
Early Assessment
Medication Review
Exercise Advice
Dietary advice
Pre-op HbA1c
Assessment of glycaemic control
Surgery
Inpatient Outcomes
Infection, Recovery, 30 day mortality
90-180 days
HbA1c, mortality

Southampton Clinical Trials Unit

Optimising Cardiac Surgery ouTcOmes in People with diabeteS

OCTOPUS Intervention Manual

Version Number 6
Date: 9-Jun-2020

IRAS reference number: 283351

For peer review only

For peer review only - http://bmjopen.bmj.com/site/about/guidelines.xhtml

Welcome to the OCTOPuS Intervention Manual

The OCTOPuS intervention has been designed for people with diabetes who have been listed for cardiac surgery involving a sternotomy. The aim of the intervention is to improve diabetes control in the run up to surgery, with the goal of improving the surgical and post-surgical experience. Although the focus of the intervention is to improve glucose control, the intervention also includes the management of other aspects of diabetes, such as weight, that are known to affect surgical outcomes.

The intervention begins once an individual has been accepted for cardiac surgery and continues until the individual has had their cardiac surgery or the surgery is cancelled. Following discharge or if the surgery is cancelled, an individual's diabetes care will revert to their usual care prior to listing for surgery.

The OCTOPuS intervention will be tested in a randomised controlled trial, funded by the National Institute for Health Research Health Technology Assessment programme.

It is hoped that if the intervention is successful in improving surgical outcomes, it can be implemented in the National Health Service and adapted for other major surgery.

For peer review only

For peer review only - http://bmjopen.bmj.com/site/about/guidelines.xhtml

Change Control

Date	Version	Activity	Who
**19th Dec 2018**	2	Clarification on how many support calls are required.	Liz Dixon
**15th August 2019**	3	Clarification on timing of intervention and managing surgical queries.	Liz Dixon
**14th October 2019**	4	1. Additional support call post-surgery<br>2. Note regarding retinopathy	Giorgos Dritsakis, Richard Holt
**16th January 2020**	5	Changes regarding frequency of telephone calls and HbA1c measurement	Richard Holt
**9th June 2020**	6	Addition of remote initial consultation option	Richard Holt

For peer review only

For peer review only - http://bmjopen.bmj.com/site/about/guidelines.xhtml

Contents

1 ABOUT OCTOPUS AND THIS MANUAL 5

1.1 WHAT IS THE OCTOPUS INTERVENTION? 5
1.2 WHY IS THE OCTOPUS INTERVENTION NEEDED? 5
1.3 WHO IS THE OCTOPUS INTERVENTION FOR? 6
1.4 ABOUT THIS MANUAL 6

2 THE OCTOPUS INTERVENTION 7

2.1 THE OCTOPUS PRACTITIONER 7
2.2 GLUCOSE MANAGEMENT 7
2.2.1 Glucose monitoring 8
2.2.2 Lifestyle modification 9
2.2.3 Drug therapy for people with type 2 diabetes not currently receiving antidiabetes medication . 10
2.2.4 People with type 2 diabetes currently receiving oral antidiabetes medication 11
2.2.5 People with type 2 diabetes already on insulin 11
2.2.6 People with type 1 diabetes 11
2.2.7 Summary of antidiabetes medication options 12
2.2.8 Diabetes management during admission for cardiac surgery 14
2.3 LIPID MANAGEMENT 15
2.4 HYPERTENSION MANAGEMENT 15
2.5 WEIGHT MANAGEMENT 16
2.6 EXERCISE 16
2.7 SMOKING CESSATION 17
2.8 INVOLVEMENT OF SPOUSES, OR OTHER RELATIVES AND FRIENDS 17

3 DELIVERING THE OCTOPUS INTERVENTION 18

3.1 THE SCHEDULE OF EVENTS 18
3.1.1 The Initial Assessment 18
3.1.2 The Fortnightly Phone Call 20
3.1.3 Surgery and Beyond 21

APPENDIX 1: THE OCTOPUS RANDOMISED CONTROLLED TRIAL 22

APPENDIX 2: BIBLIOGRAPHY 23

Figures

Figure 1 - Diabetes medication flowchart for people with type 2 diabetes; based on the 2018 American Diabetes Association care standards....................12
Figure 2 - A high-level flowchart of the OCTOPuS Intervention 18

Tables

Table 1 - Framework for Initial Assessment 19
Table 2 - Framework for Fortnightly Phone Call 20

For peer review only - http://bmjopen.bmj.com/site/about/guidelines.xhtml

1 About OCTOPuS and this manual

1.1 What is the OCTOPuS Intervention?

The OCTOPuS intervention is a set of elements, designed to improve the management of a person with diabetes over the weeks preceding scheduled major surgery. The components of the OCTOPuS intervention represent current best clinical practice and are endorsed by NICE or other guidelines. Suitably qualified and trained clinicians will deliver OCTOPuS.

The planned randomised evaluation (see Appendix 1) has been designed to assess the use of the OCTOPuS intervention for people with inadequately controlled diabetes undergoing cardiothoracic surgery, and this manual assumes that setting.

1.2 Why is the OCTOPuS intervention needed?

There are approximately 4 million people living with diagnosed and undiagnosed diabetes mellitus in the UK. Since 1996, the number of people diagnosed with diabetes has increased from 1.4 million to around 3.5 million. Diabetes increases the risk of cardiovascular disease by approximately two fold after adjustment for other cardiovascular risk factors. Ischaemic heart disease is by far the leading cause of death in people with diabetes accounting for approximately two-thirds of all deaths in those aged >65 years. Coronary heart disease tends to be more diffuse and progresses more rapidly in people with diabetes, which may explain why up to 35% of those presenting for elective cardiac revascularisation have diabetes.

The increasing number of people with diabetes will increase the demand for cardiac surgery in the future. These individuals have longer lengths of hospital stay and higher re-admission rates, placing a large financial burden on the NHS.

Poor glycaemic control increases the risk of wound and chest infections, renal impairment and death, especially following cardiac surgery. The Joint British Diabetes Societies for in-patient care has provided recommendations to improve the management of adults with diabetes undergoing surgery. As poor peri-operative glycaemic control is associated with an increased risk of all surgical complications, the guidelines recommend improving glycaemic control to optimise surgical outcomes.

Hyperglycaemia, however, does not wholly explain poorer surgical outcomes of people with diabetes; other important risk factors, such as obesity, hypertension and dyslipidaemia, are also more common in people with diabetes. Furthermore, lack of knowledge and training of the nursing and medical teams might also contribute.

If the pre-operative intervention is successful in improving glycaemic control and addressing other risk factors, this may reduce the complication rate and improve the clinical outcomes. It may also prove cost effective and even cost saving.

For peer review only - http://bmjopen.bmj.com/site/about/guidelines.xhtml

1.3 Who is the OCTOPuS intervention for?

The intervention can be offered to any person with sub-optimally controlled diabetes, which is defined as an HbA_{1c} >53 mmol/mol (>7%) using a point-of-care test at the cardiothoracic outpatient appointment where the decision to proceed to surgery is made.

The intervention described here assumes there is a period of at least 10 to 12 weeks before the scheduled surgery, but patients may derive benefit from a shorter intervention. In some circumstances, surgery may be delayed beyond 12 weeks. In this instance the pre-operative OCTOPuS intervention should continue until the patient is admitted surgery.

OCTOPuS is not suitable for people with malignancy, women who are pregnant or those with other illnesses or conditions that would preclude engagement with the intervention.

1.4 When should this manual be used?

The OCTOPuS intervention should begin as soon as possible after an individual has been accepted for cardiac surgery. The intervention continues until the individual has had their cardiac surgery or the surgery is cancelled. Following discharge or if the surgery is cancelled, an individual's diabetes care will revert to their usual care prior to listing for surgery.

1.5 About this manual

This manual describes the OCTOPuS intervention. At the time of writing, the intervention is still under development. The latest version of the manual can be obtained from the SCTU website (www.southampton.ac.uk/ctu) or from the OCTOPuS trial manager (octopus@soton.ac.uk).

This section has described the background and justification for the intervention.

Section 2 describes the components of the intervention, with a discussion of how each component might be delivered individually.

Section 3 discusses how these components are brought together and delivered as a coherent intervention.

For peer review only

For peer review only - http://bmjopen.bmj.com/site/about/guidelines.xhtml

2 The OCTOPuS Intervention

In this section, we describe the individual components of the intervention. How these components are tied together and delivered to the patient is described in section 3.

The OCTOPuS intervention comprises several elements, which are brought together in a systematic way. It is the role of the OCTOPuS practitioner to work with the patient awaiting surgery to decide which elements of the programme are applicable to the individual.

The recommendations in this manual represent the views of the OCTOPuS research team and are presented after careful consideration of the evidence and currently available NICE and international guidelines. When making treatment decisions with the participants, OCTOPuS practitioners are expected to take this manual into account, alongside the individual needs, preferences and values of their trial participants. It is not mandatory to apply the recommendations in the manual, and the guideline does not override the responsibility to make decisions appropriate to the circumstances of the individual, in consultation with them and their families and carers or guardian.

Given the short duration of the intervention and limited number of contacts, it may not be feasible to implement all actions and changes suggested by this manual. The OCTOPuS practitioner should work with the patient to implement as much as possible, planning to bring the patient to the best clinical status prior to surgery, but not delaying the planned surgical procedure in order to deliver OCTOPuS.

The sections below provide guidance in how to decide which elements might benefit each individual and guide the decision-making process. In general, the management below should follow NICE guidance for the management of diabetes, hypertension, dyslipidaemia and obesity unless specifically described otherwise.

2.1 The OCTOPuS practitioner

The OCTOPuS practitioner is a clinically qualified health care worker with expertise in diabetes. They are most likely to be a diabetes nurse specialist, but might, for example, be a pharmacist, dietitian or physician. The OCTOPuS practitioner will receive additional specific training about the OCTOPuS intervention.

Once a plan has been agreed, the practitioner supports the patient through regular contact (at least fortnightly until optimised), encouragement and counselling, signposting, and referral to key services.

Each practitioner will work with several patients awaiting surgery, providing initial advice, then remote telephone follow-up over the 3 months or so until the cardiac procedure. The OCTOPuS practitioner will need to provide advice about medication regimens, direct patients to local services, to advocate on patient's behalf, and to provide a listening ear to the patient.

2.2 Glucose Management

All patients eligible for the intervention will have an HbA_{1c} of >53mmol/mol (7.0%) and may benefit from improved glucose control. The challenge is to improve control without inducing episodes of hypoglycaemia, or excessive weight gain. The decision to intensify therapy should be made on an individual basis; for example, a more relaxed target may be appropriate for a

For peer review only - http://bmjopen.bmj.com/site/about/guidelines.xhtml

person with significant co-morbidities or where the risk and consequence of hypoglycaemia are high. There is a risk of worsening retinopathy in people with existing retinopathy if there is a rapid decline in HbA1c. The OCTOPuS practitioner should enquire whether the participant has a history of retinopathy and whether their retinal screening is up-to-date. The OCTOPuS practitioner should consider retinopathy when discussing treatment changes.

Improving glucose control may require lifestyle intervention, medication change or a combination of these strategies. How they can be delivered will be determined by local policies and funding. If the OCTOPuS practitioner is able to prescribe then they may undertake any initial changes themselves; otherwise, they should make recommendations to the patient's GP or local specialist diabetes team.

The patient's diabetes management and medication should be considered at every OCTOPuS interaction. The process is outlined in this section and summarised in Figure 1 on page 13. The OCTOPuS practitioner should provide the patient with a Diabetes UK Information Prescription about glycaemic management at each visit, if appropriate, to support glycaemic management.

2.2.1 Glucose monitoring

If the patient is not already monitoring their blood glucose, the OCTOPuS practitioner should provide the patient with a capillary glucose monitor and sufficient strips for the duration of the intervention (approximately 100 strips). They should teach the patient how to monitor and interpret their glucose using a standardised education package. All participants with Type 2 diabetes should be given a copy of 'Your Guide to Type 2 Diabetes' education booklet produced by Diabetes UK to keep for their personal reference and use throughout the study. Where applicable, participants should be shown online software management systems such as the Accu-Chek 360° Diabetes Management System. Although glucose monitoring is not usually recommended for routine use in people not using insulin or sulfonylureas, this is an important component of the intervention as short-term improvements in glucose control may not be apparent from changes in HbA_{1c} because of the short duration of the intervention.

In addition to any glucose monitoring that the patient is already undertaking prior to the intervention, patients should be advised to check their glucose levels 4 times a day (before meals and before bed) on the 3 days prior to the next OCTOPuS contact. The OCTOPuS practitioner should provide the patient with a glucose and diet diary so that the results can be recorded prior to the consultation. The OCTOPuS practitioner should encourage the patient to record what they eat in the diary so that any relationship between glucose readings and reported diet can be discussed at the consultation.

As most consultations will be remote, e.g. by telephone or Skype, where possible, the results should be sent to the OCTOPuS practitioner before the consultation, e.g. by email or Diasend (where available).

Following discussion between the OCTOPuS practitioner and patient, the glucose targets should be individualised, taking into account the risk of hypoglycaemia. However, typical glucose targets would be:

For peer review only - http://bmjopen.bmj.com/site/about/guidelines.xhtml

- Fasting and pre-meal glucose values: 4.0-6.0 mmol/L
- Post-meal and before bed glucose values: 4.0-7.8 mmol/L (for people using insulin who are prepared to test after meals)

2.2.2 Lifestyle modification

Dietary and exercise advice should follow the 2018 Diabetes UK nutritional guidelines (https://www.diabetes.org.uk/professionals/position-statements-reports/food-nutrition-lifestyle/evidence-based-nutrition-guidelines-for-the-prevention-and-management-of-diabetes). Where necessary, a healthcare professional with specific expertise and competencies in nutrition should see the patient.

Encourage high-fibre, low-glycaemic-index sources of carbohydrate in the diet, such as fruit, vegetables, wholegrains and pulses; include low-fat dairy products and oily fish; and control the intake of foods containing saturated and trans fatty acids. Encourage the patients to avoid excessive alcohol consumption.

The following leaflets may be of benefit:

British Heart Foundation:
Food labelling guidance: https://www.bhf.org.uk/publications/healthy-eating-and-drinking/this-label-could-change-your-life

Weight Loss advice:
https://www.bhf.org.uk/publications/healthy-eating-and-drinking/facts-not-fads---your-simple-guide-to-healthy-weight-loss

Nutrition and Diet Resource
Weight loss advice: describes 80kcal portions of different food groups so that the participants can decide how many of each they can have i.e. 1500kcal
https://www.ndr-uk.org/item/81/WeightManagement/Weight-Loss-You-Can-See-with-guidelines.html
This is a paid resource but could be useful for more visual patients

Carbs and cals – a variety of recipes, carb values, and low calorie meal options:
https://www.carbsandcals.com/weight-loss/weight-loss

British Dietetic Association:
Basic diet sheets for glycaemic index, healthy eating and weight loss
https://www.bda.uk.com/foodfacts/GIDiet.pdf
https://www.bda.uk.com/foodfacts/HealthyEating.pdf
https://www.bda.uk.com/foodfacts/Want2LoseWeight.pdf

For individuals who are overweight or obese, weight loss should be encouraged (see below).

For peer review only - http://bmjopen.bmj.com/site/about/guidelines.xhtml

The OCTOPuS practitioner should encourage the patient to become more physically active (see below).

2.2.3 Drug therapy for people with type 2 diabetes not currently receiving anti-diabetes medication

Almost all patients will need anti-diabetes drug therapy because of the elevated HbA_{1c} and short window-of-opportunity to improve the glucose control. The OCTOPuS practitioner should use their clinical judgement in the choice of medication, which should be individualised in discussion with the patient. All prescribing advice should be within the drug licence and take account of the individual Summary of Product Characteristics.

In line with NICE and 2018 American Diabetes Association guidance, the first line treatment of choice is standard release metformin, unless contraindicated. Metformin should be used according to its licence. Slow release metformin can be considered if the standard released metformin is not tolerated.

Where the initial HbA_{1c} is ≥75 mmol/mol (9%), consideration should be given to starting dual therapy from the outset as recommended by the 2018 American Diabetes Association guidance.

If metformin is insufficient to achieve adequate glycaemic control (as judged by capillary glucose monitoring described above), a second agent should be added. Because of the short-time scale of the intervention, it is not possible to base treatment changes on the measurement of HbA_{1c} for most patients. As all patients will have existing atherosclerotic cardiovascular disease, in line with the 2018 American Diabetes Association guidance, the first treatment intensification should usually be with a drug that has proven cardiovascular benefit, e.g. an SGLT-2 inhibitor (e.g. empagliflozin or canagliflozin) or a GLP-1 receptor agonist (e.g. liraglutide), unless contraindicated. The second treatment intensification should be the other class of drug (i.e. if an SGLT2 inhibitor was the first intensification then the second should be GLP-1 receptor agonist or vice versa), unless contraindicated. OCTOPuS practitioners will need to take account of drug-specific and patient factors as well as local formulary requirements.

The 2018 American Diabetes Association guidance diverges from current NICE guidance as the latter has not yet been updated in light of the latest cardiovascular outcome trials. However, there is a need to avoid weight gain and hypoglycaemia in this group of patients and there is a need to avoid provoking cardiovascular events as occurred in the ACCORD study.

Where these drugs are contraindicated, alternative agents, such as DPP-4 inhibitors, pioglitazone or sulfonylureas, e.g. gliclazide, can be used according to NICE guidance.

If adequate glycaemic control is not achieved with three non-insulin therapies, insulin should be initiated according to NICE guidance (see below). Where the initial HbA_{1c} is ≥86 mmol/mol

For peer review only - http://bmjopen.bmj.com/site/about/guidelines.xhtml

(10%), consideration should be given to starting insulin therapy from the outset as recommended by the 2018 American Diabetes Association guidance.

2.2.4 People with type 2 diabetes currently receiving oral anti-diabetes medication

Lifestyle factors, such as diet and physical activity, should be explored but it is likely that drug therapy will need to be intensified.

The OCTOPuS practitioner should explore whether the patient is taking the medication as prescribed. It is known that fewer than 50% of people receiving oral anti-diabetes treatments, antihypertensive agents and statins persist with their medication 2 years after treatment initiation and up to 20% never start treatment. The barriers to adherence should be discussed with the patient.

Where drug therapy intensification is needed, this should be done as described in the previous section 2.2.3. If the individual is on other combinations of oral anti-diabetes agents, the OCTOPuS practitioner should consider whether to change these to treatments with proven cardiovascular benefit.

2.2.4.1 Commencement of Insulin in people with type 2 diabetes

If adequate glycaemic control (judged by capillary glucose monitoring or presenting HbA_{1c}) is not achieved with three non-insulin therapies, insulin should be initiated according to NICE guidance. Basal insulin alone is the most convenient initial insulin regimen, beginning at 10 units per day or 0.1–0.2 units/kg/day, depending on the degree of hyperglycaemia.

As hypoglycaemia is a major risk factor for cardiovascular events, the OCTOPuS practitioner should have a low threshold to initiate insulin analogues instead of NPH insulin because of the lower risk of hypoglycaemia seen with the use of insulin analogues.

The OCTOPuS practitioner will need to liaise with local health services to ensure that the patient is offered sufficient training to use the insulin effectively. Given the urgency of treatment, in many instances, this will need to be outside usual channels.

Intensification of insulin is usually by the addition of prandial insulin or switch to pre-mixed insulin. The options should be discussed with the patient and a management plan agreed with the patient.

2.2.5 People with type 2 diabetes already on insulin

The OCTOPuS practitioner should review the current insulin regimen, injection technique and sites with the patient. The OCTOPuS practitioner should offer advice about the doses and types of injection as necessary.

2.2.6 People with type 1 diabetes

It is likely that the OCTOPuS practitioner will see people on a variety of insulin regimens, including both multiple daily injection and insulin pump therapy. The OCTOPuS practitioner

For peer review only - http://bmjopen.bmj.com/site/about/guidelines.xhtml

should review the current insulin regimen, injection technique and sites with the patient. The OCTOPuS practitioner should offer advice about the doses and types of injection as necessary.

If the patient has not attended a structured education course, this should be offered where possible.

A detailed description of insulin therapy is beyond the scope of this manual and the OCTOPuS practitioner should refer to the NICE type 1 diabetes guidance.

2.2.7 Summary of anti-diabetes medication options

The OCTOPuS practitioner should provide the patient with a Diabetes UK Information Prescription about glycaemic management at each visit to support glycaemic management.

Figure 1 summarises the options available to improve glycaemic control prior to surgery. The OCTOPuS practitioner should discuss progress with the patient at every visit or fortnightly phone call and management plan adjusted accordingly.

For peer review only

For peer review only - http://bmjopen.bmj.com/site/about/guidelines.xhtml

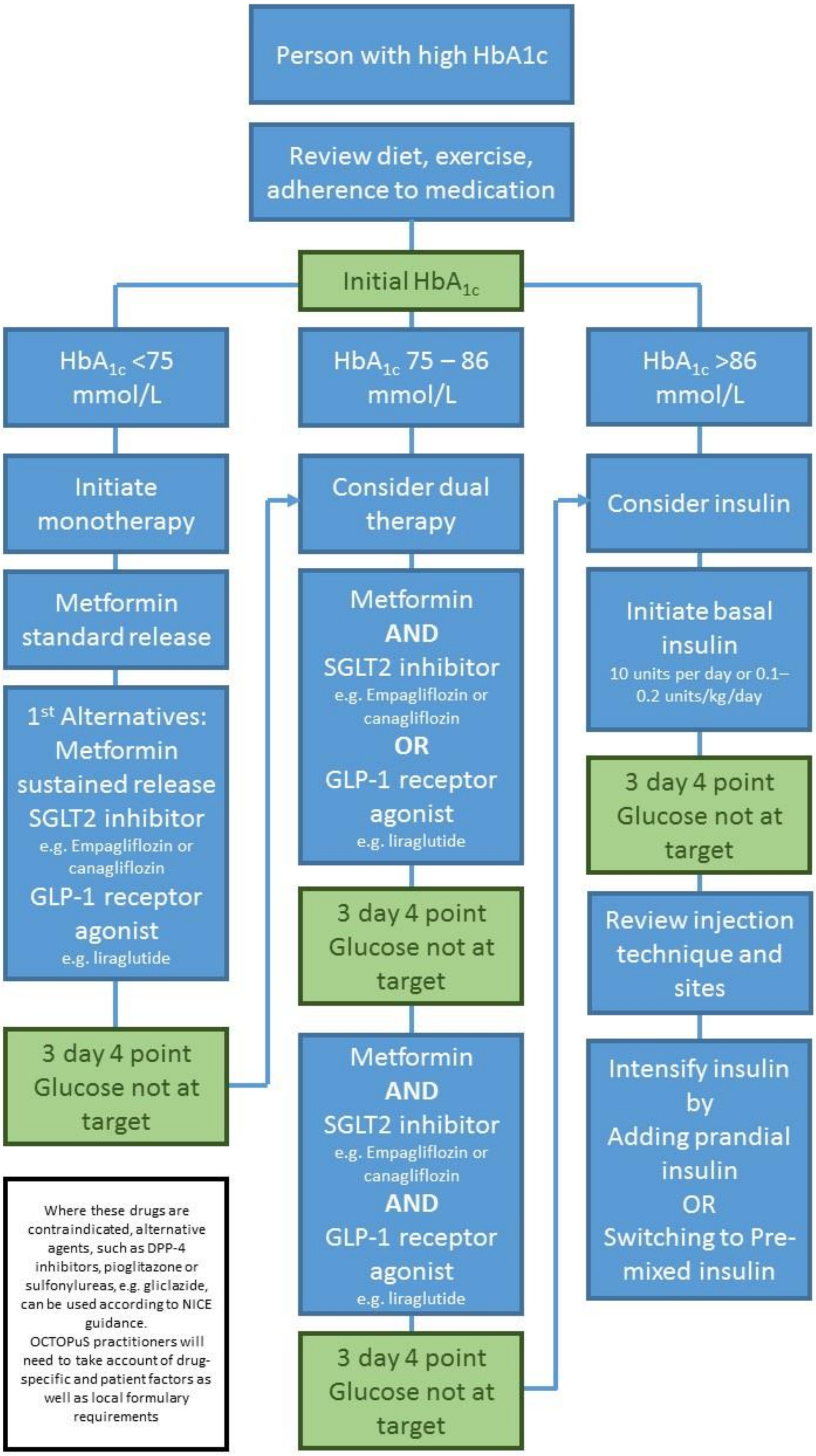

Figure 1 - Diabetes medication flowchart for people with type 2 diabetes; based on the 2018 American Diabetes Association care standards.

For peer review only - http://bmjopen.bmj.com/site/about/guidelines.xhtml

2.2.8 Diabetes management during admission for cardiac surgery

The OCTOPuS practitioner will need to provide advice about medication and glucose management when the patient is admitted for surgery. Where possible, the OCTOPuS practitioner should arrange to see the patient after admission but prior to surgery to provide advice about diabetes management during the admission.

The OCTOPuS practitioner may need to liaise with the diabetes in-patient team in the cardiothoracic centre, if not already a member of this team. Local protocols and JBDS guidance should be followed (http://www.diabetologists-abcd.org.uk/JBDS/Surgical_guidelines_2015_full_FINAL_amended_Mar_2016.pdf). There may be specific questions about cardiac surgery or management of diabetes during the operation that the OCTOPuS practitioner is unable to answer. If this is the case, the OCTOPuS practitioner should alert the cardiac surgeon so that the surgical team can answer these questions.

The OCTOPuS practitioner should advise the patient to continue to monitor their glucose, where appropriate. They should warn the patient of the risk of hypoglycaemia during fasting prior to admission. The OCTOPuS practitioner should advise the patient that oral anti-diabetes medications and GLP-1 receptor agonists need to be omitted on the day of surgery. The OCTOPuS practitioner should provide advice about adjustments to insulin doses. Suggested adjustments to insulin doses are as follows:

**Insulin**	**Day before procedure**	**Day of procedure**
Once daily (evening) (e.g. Lantus, Levemir, Tresiba, Abasalaglar Insulatard or Humulin I, Toujeo)	Take 80% of usual insulin dose at usual time.	Take 80% of usual insulin dose in the evening after the procedure
Once daily (morning) (e.g. Lantus, Levemir, Tresiba, Insulatard or Humulin I)	Take usual time.	Take 80% of usual insulin dose in the evening after the procedure
Twice daily (e.g. Novomix 30, Humulin M3, Humalog Mix 25 or 50, Lantus, Levemir)	Take usual time	Omit morning dose
Meal time injection	Take usual time	Omit all rapid insulin
Insulin pump	Please inform specialist pump team before admission for personalised advice. Continue with usual basal rates and continue to bolus depending on carbohydrate intake	Continue with usual basal rates and continue to bolus depending on carbohydrate intake

For peer review only - http://bmjopen.bmj.com/site/about/guidelines.xhtml

Following surgery but prior to discharge, the OCTOPuS practitioner should see the patient to discuss post-operative diabetes management. In some situations, it may be appropriate to discontinue insulin therapy. Any treatment changes and on-going management plans should be communicated to the patient's GP and specialist diabetes team, if necessary, following discharge after surgery. This information could be included in the discharge summary or communicated separately depending on local arrangements.

2.3 Lipid Management

Most patients will already be taking lipid-lowering therapy. However, if they are not taking a statin, the OCTOPuS practitioner should discuss the benefits of statin therapy and recommend that this is initiated if there are no contraindications or the person has previously not tolerated treatment with statins.

For those already taking lipid-lowering therapy, the OCTOPuS practitioner should review the patient's latest lipid profile. If a greater than 40% reduction in non-HDL cholesterol has not been achieved, the OCTOPuS practitioner should discuss adherence and timing of dose, optimise adherence to diet and lifestyle measures and consider increasing the statin dose if the participant is taking less than atorvastatin 80 mg. The OCTOPuS practitioner should consider the addition of ezetimibe. In some circumstances, these measures may be insufficient to control the lipid profile and in this situation, the OCTOPuS practitioner should considering recommending a referral to a specialist lipid clinic for consideration of PCSK9 inhibitors if this is available within the timeframe of the intervention.

Where changes have been recommended, the OCTOPuS practitioner should provide the patient with a Diabetes UK Information Prescription about lipid management.

2.4 Hypertension Management

Hypertension and endothelial dysfunction are common in people with diabetes. The OCTOPuS practitioner should measure the blood pressure or record the clinic blood pressure measurement as part of the initial assessment.

Preliminary data suggest that preoperative use of an antagonist of renin-angiotensin system (ACE inhibitor or angiotensin receptor blocker) in people undergoing CABG is associated with decreased in-hospital mortality. Unless contraindicated, the OCTOPuS practitioner should consider an antagonist of renin-angiotensin system (ACE inhibitor or angiotensin receptor blocker) for all patients after discussion with the local cardiothoracic surgical team. The dose should be titrated against the patient's blood pressure, which should be measured in the patient's general practice or by the patient at home. Further agents may be added in accordance with NICE guidance (CG127) as necessary. When an ACE inhibitor or angiotensin receptor blocker is added, the OCTOPuS practitioner should advise the measurement of urea and electrolytes and estimated glomerular filtration rate according to standard clinical

For peer review only

For peer review only - http://bmjopen.bmj.com/site/about/guidelines.xhtml

practice. At each contact, the OCTOPuS practitioner should advise the patients to withhold ACE inhibitors or angiotensin receptor blockers from 5 days prior to surgery.

Bear in mind that the surgical team may wish to commence a beta blocker prior to surgery, so if a second line agent is needed the OCTOPuS practitioner should consider commencing a beta blocker. This decision should be discussed with the local cardiothoracic surgical team.

Where changes have been recommended, the OCTOPuS practitioner should provide the patient with a Diabetes UK Information Prescription about blood pressure management.

2.5 Weight Management

Obesity and being overweight are associated with poor surgical outcomes. People with a BMI >25 Kg/m^2 are therefore likely to benefit from weight reduction, both to improve their diabetes control and surgical outcome.

The OCTOPuS practitioner should measure the patient's height and weight and calculate their BMI.

For recommendations on weight management, see the NICE guidelines on: preventing excess weight gain, weight management, and obesity. For most patients undertaking OCTOPuS, the major element of weight reduction will be through diet. Exercise is discussed in section 2.6 below.

The OCTOPuS practitioner should refer the patient or facilitate referral to a local NHS weight reduction programme or dietitian if this can be accessed quickly enough to achieve a worthwhile effect before surgery (e.g. the programme can be started within 4 weeks).

As described above, Diabetes UK and the British Heart Foundation both produce excellent leaflets about healthy eating and the OCTOPuS practitioner should provide the patient with a copy of these or let the patient know how to access them.

If an NHS option is not available, then other weight reduction options should be explored. This could include commercial programmes, such as Weight Watchers, or a self-managed diet.

2.6 Exercise

Physical activity has profound benefits for people with diabetes, including improved fitness, reduced insulin requirement and better glycaemic control, lower cardiovascular risk (lower blood pressure and improved lipid profile) and improved survival. People with diabetes should take at least 150 minutes of exercise per week spread over a minimum of 3 days with a mixture of aerobic exercise and resistance training.

However, care is needed in this group of patients as those awaiting cardiac revascularisation surgery may experience angina on exertion and have a limited capacity to provide oxygenated blood to cardiac muscle. Similarly, those awaiting valve surgery may not have significant capacity to exercise. Therefore, this component of the intervention will require the OCTOPuS practitioner to tailor any recommendation to the capacity of the individual.

For peer review only

For peer review only - http://bmjopen.bmj.com/site/about/guidelines.xhtml

It is also important to ascertain whether the individual has diabetes microvascular complications before starting to exercise. People with a history of active foot ulcers should avoid weight-bearing exercise and appropriate footwear should be worn.

However, where possible, the OCTOPuS practitioner should encourage patients to take gentle exercise, such as walking or dancing. The use of pedometers can support a gradual increase in physical activity. The best exercise is the one that the person enjoys. Practical advice should be given to help the person with diabetes find ways to become more physically active.

In the case of Type 1 diabetes, exercise can lead to unstable glucose levels during and immediately after exercise and a later risk of severe hypoglycaemia. Patients should be advised to avoid exercise of this intensity. As prior hypoglycaemia blunts the catecholamine response, people should be advised to avoid exercise within 24 hours of a severe hypoglycaemic episode and 1 hour of a self-treated episode. Exercise should also be avoided if blood ketone levels are increased. The OCTOPuS practitioner should consider whether a referral to a consultant diabetologist is required to address this complex area.

In type 2 diabetes, exercise does not usually cause hypoglycaemia and so carbohydrate supplementation is not required.

2.7 Smoking Cessation

All patients should be encouraged and supported to stop smoking, where possible. For recommendations on smoking cessation, see the NICE guidelines on: smoking: brief interventions and referrals, stop smoking services, and smoking: harm reduction.

2.8 Involvement of spouses, or other relatives and friends

The involvement of people important in the patient's life may lead to greater adherence to the components of the OCTOPuS intervention. Encouragement and support from friends and family will make the changes in medication, diet, and other activity, more sustainable over the few weeks that the intervention is delivered.

Therefore, if possible friends and relatives should be involved in the initial consultation, and consideration should be given to including them in the fortnightly phone calls.

For peer review only - http://bmjopen.bmj.com/site/about/guidelines.xhtml

3 Delivering the OCTOPuS Intervention

The elements described in section 2 are brought together in the OCTOPuS intervention.

3.1 The Schedule of Events

The intervention follows a series of events, shown in Figure 1. At each stage, the practitioner works with the patient to agree a set of goals and actions.

Figure 1 - A high-level flowchart of the OCTOPuS Intervention

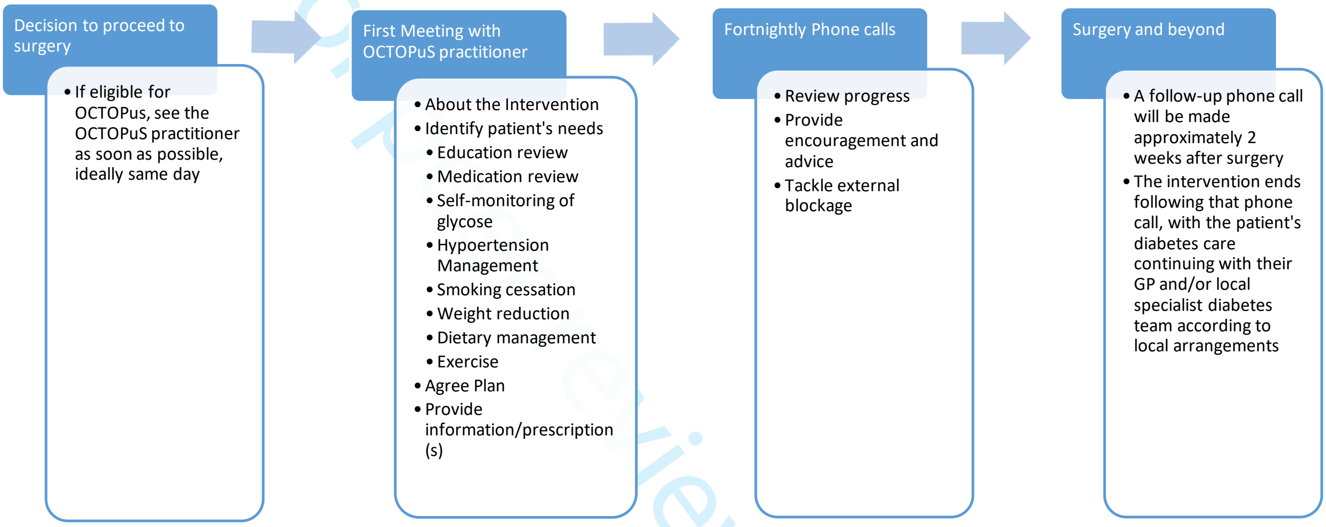

3.1.1 The Initial Assessment

The initial assessment is key to the OCTOPuS intervention. It is where the practitioner establishes a trust relationship, which will enable the patient to take the greatest advantage of the programme. We anticipate that the initial consultation will be usually be conducted face-to–face. However, alternative remote delivery options (video or telephone consultations) may be considered when these are necessary, for example, when the patient is unable to attend the hospital or to comply with COVID-19 social distancing.

For peer review only - http://bmjopen.bmj.com/site/about/guidelines.xhtml

Table 1 - Framework for Initial Assessment

Phase	Activity
**Assessment**	Explore the patient's understanding and experience of diabetes.<br>Explore any concerns the patient has relating to diabetes and their prospective surgery.<br>Establish whether there are relevant co-morbidities<br>Establish any significant people in the patient's life who could provide support in the run up to surgery.
**Explanation**	Explain the OCTOPuS intervention, and its goals, in the light of information elicited in the assessment phase.
**Measurement**	Make any clinical assessments which may be required, that weren't done in the most recent cardiovascular outpatient appointment<br>• HbA_{1c}<br>• Blood Pressure<br>• Height, Weight -> BMI
**Review**	Review the patient's situation in the areas set out in section 2 of this manual.<br>• Glucose Management<br>• Hypertension Management<br>• Weight Reduction<br>• Smoking Status<br>• Exercise<br>Agree a plan, where appropriate, with the patient for each of these elements
**Follow-up**	Schedule the first fortnightly phone call. You may like to schedule multiple calls for the complete period up to the planned surgery date.<br>Obtain permission from the patient to contact their GP or other services to support the patient's action plan. Liaise with local clinical team where necessary<br>Put into place any arrangements needed to support the patient's action plan

For peer review only

For peer review only - http://bmjopen.bmj.com/site/about/guidelines.xhtml

3.1.2 The Fortnightly Phone Call

At least every fortnight, the OCTOPuS practitioner will contact the patient until the patient's diabetes management is optimised or no further changes are possible. After this has been achieved, the frequency of the calls can be reduced at the discretion of the OCTOPuS practitioner to a minimum of every 6 weeks. We envisage this being by phone but other methods (face-to-face, Skype etc.) could be used by mutual agreement.

This is an opportunity for the practitioner to review progress against goals, and for the patient to raise any queries they might have.

The OCTOPuS practitioner should ensure that the patient has the contact details of the OCTOPuS team so that they can contact the team if necessary.

Table 2 - Framework for Fortnightly Phone Call

Phase	Activity
**Assessment**	Explore the patient's activity and progress against the goals agreed at the previous initial assessment or fortnightly phone call<br><br>Explore whether there has been any changes health, e.g. infection, that might affect the management plan.
**Measurement**	Make any clinical assessments, which may be required. This will generally involve the patients reporting over the phone or by alternative means of communication, such as email, DIASEND, Freestyle Libreview etc.<br>• Recent self-monitoring of blood glucose<br>• Weight<br>• Smoking status
**Listen**	To any problems or concerns that the patient raises

For peer review only

For peer review only - http://bmjopen.bmj.com/site/about/guidelines.xhtml

Phase	Activity
**Review**	Review the patient's situation in the areas set out in section 2 of this manual.<br>• Glucose Management – where surgery is delayed beyond 3 months, the OCTOPuS practitioner should arrange for a further HbA1c measurement every 3 months<br>• Hypertension Management<br>• Weight Reduction<br>• Smoking Status<br>• Exercise<br>Agree a plan, where appropriate, with the patient for each of these elements, in the light of the review and listening phases.<br>Liaise with local clinical team where necessary
**Follow up**	Schedule the next fortnightly phone call, if not already done.

3.1.3 Surgery and Beyond

Surgery will proceed according to local protocols.

Approximately two weeks after surgery, the OCTOPuS practitioner will contact the patient again. This phone call will follow the framework in Table 2 above. During the final support call, the OCTOPuS practitioner should undertake a review of the elements of the intervention and develop a future diabetes management plan.

All patients who have completed the OCTOPuS intervention and their surgical treatment should then return to routine diabetes care with their GP or local diabetes specialist team.

For peer review only

For peer review only - http://bmjopen.bmj.com/site/about/guidelines.xhtml

Appendix 1: The OCTOPuS Randomised Controlled Trial

The OCTOPuS trial has been funded by the NIHR HTA programme to evaluate the OCTOPuS intervention, and to determine whether it adds value to patient care.

Approximately 426 people with poorly controlled diabetes undergoing cardiac surgery will be randomised to either the OCTOPuS intervention or to usual care. The outcomes of interest include time from surgery until clinically for hospital discharge, 30 & 90 day mortality, wound infections, chest infections, renal impairment, HbA_{1c} pre-op and, 90 days post op, cost-effectiveness, procedures cancelled due to glycaemic control, quality of life, patient satisfaction & experience.

More information is available at https://www.journalslibrary.nihr.ac.uk/programmes/hta/162512/#/

For peer review only

For peer review only - http://bmjopen.bmj.com/site/about/guidelines.xhtml

Southampton Clinical Trials Unit

Appendix 2: Bibliography

1 Zheng J, Cheng J, Wang T, *et al.* Does HbA1c Level Have Clinical Implications in Diabetic Patients Undergoing Coronary Artery Bypass Grafting? A Systematic Review and Meta-Analysis. *Int J Endocrinol* 2017;**2017**:1537213–8. doi:10.1155/2017/1537213

2 Rollins KE, Varadhan KK, Dhatariya K, *et al.* Systematic review of the impact of HbA1c on outcomes following surgery in patients with diabetes mellitus. *Clinical Nutrition* 2016;**35**:308–16. doi:10.1016/j.clnu.2015.03.007

3 Boreland L, Scott-Hudson M, Hetherington K, *et al.* The effectiveness of tight glycemic control on decreasing surgical site infections and readmission rates in adult patients with diabetes undergoing cardiac surgery: A systematic review. *Heart Lung* 2015;**44**:430–40. doi:10.1016/j.hrtlng.2015.06.004

4 Cheng X, Tong J, Hu Q, *et al.* Meta-analysis of the effects of preoperative renin–angiotensin system inhibitor therapy on major adverse cardiac events in patients undergoing cardiac surgery. *Eur J Cardiothorac Surg* 2015;**47**:958–66. doi:10.1093/ejcts/ezu330

5 NICE. *Hypertension in adults: diagnosis and management*. 2011.

6 NICE. *Preventing excess weight gain*. NICE 2015.

7 NICE. *Weight management: lifestyle services for overweight or obese adults*. NICE 2014.

8 NICE. *Obesity: identification, assessment and management*. NICE 2014.

For peer review only

For peer review only - http://bmjopen.bmj.com/site/about/guidelines.xhtml

PROGRESS GRADING

Actions to be taken depending on progress grade

Grade	Action
Green	Continue trial, keeping an eye on accrual.
Amber	Working with governance committees (TSC, TMG, PPI Committees), seek root cause for under performance. Consider whether these can be mitigated through work with organisations or individuals within the study.
Red	Review the study with governance committees, taking steps as detailed under amber, but also explicitly considering recommending study closure.

Progress grading time points and criteria

Assessment Point	Green	Amber	Red
After 100 patients have had surgery (50 intervention and 50 control)	HbA_{1c} reduction in intervention group >5mmol/mol	HbA_{1c} reduction in intervention group <5mmol/mol	HbA_{1c} reduction in intervention group not consistent with physiological effect

For peer review only - http://bmjopen.bmj.com/site/about/guidelines.xhtml

SAFETY CONSIDERATIONS

Definitions

Adverse Event (AE): any untoward medical occurrence in a participant or clinical study participant which does not necessarily have a causal relationship with study treatment or participation. An AE can therefore be any unfavourable and unintended sign (including an abnormal laboratory finding), symptom, or disease temporally associated with the study treatment or participation (regardless of causality assessments).

Serious Adverse Event (SAE) is any untoward medical occurrence or effect that:

- Results in death
- Is life-threatening, i.e. the participant was at risk of death at the time of the event
- Requires hospitalisation (regardless of length of stay), or prolongation of existing hospitalisation (>30 days post- cardiothoracic surgery)
- Results in persistent or significant disability or incapacity
- Is a congenital anomaly or birth defect
- Other important medical events (if they jeopardise the participant or require an intervention to prevent one of the above consequences).

It is the responsibility of the PI or delegate to grade an event as 'not serious' (AE) or 'serious' (SAE).

Seriousness

A complete assessment of the seriousness must always be assessed by a medically qualified doctor who is registered on the delegation of responsibility log; this is usually the investigator. All SAEs must be reported immediately by the PI at the participating centre to the SCTU.

Causality & Expectedness

A complete assessment of the causality must always be assessed by a medically qualified doctor who is registered on the delegation of responsibility log; this is usually the investigator. The nature or severity should be considered when making the assessment of expectedness. If these factors are not consistent with the current information available then the AE should be recorded as 'unexpected'.

For peer review only

Reporting Procedures

All adverse events should be reported until the End of Study as defined in 3.3. SAEs should be reported to SCTU within 24 hours of site becoming aware of the event. Additional information should be provided as soon as possible if the event has not resolved at the time of reporting. The reporting requirement for all AEs and SAEs affecting participants applies for all events occurring up to 90 days following cardiac surgery.

All unresolved adverse events should be followed by the investigator until resolved, the participant is lost to follow-up, or the adverse event is otherwise explained. At the last scheduled visit, the investigator should instruct each participant to report any subsequent event(s) that the participant, or the participant's general practitioner, believes might reasonably be related to participation in this study. The investigator should notify the study sponsor of any death or adverse event occurring at any time after a participant has discontinued or terminated study participation that may reasonably be related to this study.

Medically significant pre-existing conditions (those which are present prior to informed consent) should not be reported as an AE unless the conditions worsens during the trial. The condition, however, must be reported on the Medical History eCRF. Any adverse events which occur after informed consent taken should be recorded on the AE eCRF as per safety reporting section.

All SAEs should be reported within 24 hours of the local site becoming aware of the event. The SAE Non-CTIMP Form asks for nature of event, date of onset, severity, corrective therapies given, outcome, causality (i.e. unrelated, unlikely, possible, probably, definitely) and expectedness. The responsible investigator should assign the causality and expectedness of the event with reference to the events listed in Section 6.4.1.

For peer review only

Reporting checklist for protocol of a clinical trial.

Based on the SPIRIT guidelines.

Instructions to authors

Complete this checklist by entering the page numbers from your manuscript where readers will find each of the items listed below.

Your article may not currently address all the items on the checklist. Please modify your text to include the missing information. If you are certain that an item does not apply, please write "n/a" and provide a short explanation.

Upload your completed checklist as an extra file when you submit to a journal.

In your methods section, say that you used the SPIRITreporting guidelines, and cite them as:

Chan A-W, Tetzlaff JM, Gøtzsche PC, Altman DG, Mann H, Berlin J, Dickersin K, Hróbjartsson A, Schulz KF, Parulekar WR, Krleža-Jerić K, Laupacis A, Moher D. SPIRIT 2013 Explanation and Elaboration: Guidance for protocols of clinical trials. BMJ. 2013;346:e7586

		Reporting Item	Page Number
**Administrative information**			
Title	#1	Descriptive title identifying the study design, population, interventions, and, if applicable, trial acronym	1
Trial registration	#2a	Trial identifier and registry name. If not yet registered, name of intended registry	2
Trial registration: data set	#2b	All items from the World Health Organization Trial Registration Data Set	n/a
Protocol version	#3	Date and version identifier	n/a
Funding	#4	Sources and types of financial, material, and other support	17
Roles and responsibilities: contributorship	#5a	Names, affiliations, and roles of protocol contributors	1, 17

For peer review only

Roles and responsibilities: sponsor contact information	#5b	Name and contact information for the trial sponsor	16
Roles and responsibilities: sponsor and funder	#5c	Role of study sponsor and funders, if any, in study design; collection, management, analysis, and interpretation of data; writing of the report; and the decision to submit the report for publication, including whether they will have ultimate authority over any of these activities	n/a
Roles and responsibilities: committees	#5d	Composition, roles, and responsibilities of the coordinating centre, steering committee, endpoint adjudication committee, data management team, and other individuals or groups overseeing the trial, if applicable (see Item 21a for data monitoring committee)	14
**Introduction**			
Background and rationale	#6a	Description of research question and justification for undertaking the trial, including summary of relevant studies (published and unpublished) examining benefits and harms for each intervention	4
Background and rationale: choice of comparators	#6b	Explanation for choice of comparators	7-8
Objectives	#7	Specific objectives or hypotheses	5
Trial design	#8	Description of trial design including type of trial (eg, parallel group, crossover, factorial, single group), allocation ratio, and framework (eg, superiority, equivalence, non-inferiority, exploratory)	5
**Methods: Participants, interventions, and outcomes**			
Study setting	#9	Description of study settings (eg, community clinic, academic hospital) and list of countries where data will be collected. Reference to where list of study sites can be obtained	10-11
Eligibility criteria	#10	Inclusion and exclusion criteria for participants. If applicable, eligibility criteria for study centres and individuals who will	5

For peer review only

		perform the interventions (eg, surgeons, psychotherapists)	
Interventions: description	#11a	Interventions for each group with sufficient detail to allow replication, including how and when they will be administered	7-8
Interventions: modifications	#11b	Criteria for discontinuing or modifying allocated interventions for a given trial participant (eg, drug dose change in response to harms, participant request, or improving / worsening disease)	7-8
Interventions: adherance	#11c	Strategies to improve adherence to intervention protocols, and any procedures for monitoring adherence (eg, drug tablet return; laboratory tests)	7-8
Interventions: concomitant care	#11d	Relevant concomitant care and interventions that are permitted or prohibited during the trial	7-8
Outcomes	#12	Primary, secondary, and other outcomes, including the specific measurement variable (eg, systolic blood pressure), analysis metric (eg, change from baseline, final value, time to event), method of aggregation (eg, median, proportion), and time point for each outcome. Explanation of the clinical relevance of chosen efficacy and harm outcomes is strongly recommended	9-10
Participant timeline	#13	Time schedule of enrolment, interventions (including any run-ins and washouts), assessments, and visits for participants. A schematic diagram is highly recommended (see Figure)	7-8
Sample size	#14	Estimated number of participants needed to achieve study objectives and how it was determined, including clinical and statistical assumptions supporting any sample size calculations	10-11
Recruitment	#15	Strategies for achieving adequate participant enrolment to reach target sample size	6
**Methods: Assignment of interventions (for controlled trials)**			
Allocation: sequence generation	#16a	Method of generating the allocation sequence (eg, computer-generated random numbers), and list of any factors for stratification. To reduce predictability of a random sequence, details of any planned restriction (eg, blocking) should be provided in a separate document that is unavailable to those who enrol participants or assign interventions	6

For peer review only

Allocation concealment mechanism	#16b	Mechanism of implementing the allocation sequence (eg, central telephone; sequentially numbered, opaque, sealed envelopes), describing any steps to conceal the sequence until interventions are assigned	n/a
Allocation: implementation	#16c	Who will generate the allocation sequence, who will enrol participants, and who will assign participants to interventions	n/a
Blinding (masking)	#17a	Who will be blinded after assignment to interventions (eg, trial participants, care providers, outcome assessors, data analysts), and how	n/a
Blinding (masking): emergency unblinding	#17b	If blinded, circumstances under which unblinding is permissible, and procedure for revealing a participant's allocated intervention during the trial	n/a
**Methods: Data collection, management, and analysis**			
Data collection plan	#18a	Plans for assessment and collection of outcome, baseline, and other trial data, including any related processes to promote data quality (eg, duplicate measurements, training of assessors) and a description of study instruments (eg, questionnaires, laboratory tests) along with their reliability and validity, if known. Reference to where data collection forms can be found, if not in the protocol	18
Data collection plan: retention	#18b	Plans to promote participant retention and complete follow-up, including list of any outcome data to be collected for participants who discontinue or deviate from intervention protocols	18
Data management	#19	Plans for data entry, coding, security, and storage, including any related processes to promote data quality (eg, double data entry; range checks for data values). Reference to where details of data management procedures can be found, if not in the protocol	18
Statistics: outcomes	#20a	Statistical methods for analysing primary and secondary outcomes. Reference to where other details of the statistical analysis plan can be found, if not in the protocol	12-13
Statistics: additional analyses	#20b	Methods for any additional analyses (eg, subgroup and adjusted analyses)	12-13

For peer review only

Statistics: analysis population and missing data	#20c	Definition of analysis population relating to protocol non-adherence (eg, as randomised analysis), and any statistical methods to handle missing data (eg, multiple imputation)	12-13
**Methods: Monitoring**			
Data monitoring: formal committee	#21a	Composition of data monitoring committee (DMC); summary of its role and reporting structure; statement of whether it is independent from the sponsor and competing interests; and reference to where further details about its charter can be found, if not in the protocol. Alternatively, an explanation of why a DMC is not needed	n/a
Data monitoring: interim analysis	#21b	Description of any interim analyses and stopping guidelines, including who will have access to these interim results and make the final decision to terminate the trial	n/a
Harms	#22	Plans for collecting, assessing, reporting, and managing solicited and spontaneously reported adverse events and other unintended effects of trial interventions or trial conduct	n/a
Auditing	#23	Frequency and procedures for auditing trial conduct, if any, and whether the process will be independent from investigators and the sponsor	n/a
**Ethics and dissemination**			
Research ethics approval	#24	Plans for seeking research ethics committee / institutional review board (REC / IRB) approval	2
Protocol amendments	#25	Plans for communicating important protocol modifications (eg, changes to eligibility criteria, outcomes, analyses) to relevant parties (eg, investigators, REC / IRBs, trial participants, trial registries, journals, regulators)	n/a
Consent or assent	#26a	Who will obtain informed consent or assent from potential trial participants or authorised surrogates, and how (see Item 32)	6
Consent or assent: ancillary studies	#26b	Additional consent provisions for collection and use of participant data and biological specimens in ancillary studies, if applicable	n/a
Confidentiality	#27	How personal information about potential and enrolled participants will be collected, shared, and maintained in order to protect confidentiality before, during, and after the trial	16

For peer review only

Declaration of interests	#28	Financial and other competing interests for principal investigators for the overall trial and each study site	18
Data access	#29	Statement of who will have access to the final trial dataset, and disclosure of contractual agreements that limit such access for investigators	18
Ancillary and post trial care	#30	Provisions, if any, for ancillary and post-trial care, and for compensation to those who suffer harm from trial participation	n/a
Dissemination policy: trial results	#31a	Plans for investigators and sponsor to communicate trial results to participants, healthcare professionals, the public, and other relevant groups (eg, via publication, reporting in results databases, or other data sharing arrangements), including any publication restrictions	16-17
Dissemination policy: authorship	#31b	Authorship eligibility guidelines and any intended use of professional writers	n/a
Dissemination policy: reproducible research	#31c	Plans, if any, for granting public access to the full protocol, participant-level dataset, and statistical code	n/a
**Appendices**			
Informed consent materials	#32	Model consent form and other related documentation given to participants and authorised surrogates	n/a
Biological specimens	#33	Plans for collection, laboratory evaluation, and storage of biological specimens for genetic or molecular analysis in the current trial and for future use in ancillary studies, if applicable	n/a

The SPIRIT Explanation and Elaboration paper is distributed under the terms of the Creative Commons Attribution License CC-BY-NC. This checklist was completed on 02. March 2021 using https://www.goodreports.org/, a tool made by the EQUATOR Network in collaboration with Penelope.ai

For peer review only
