# Supplementary material for: The Optimising Cardiac Surgery ouTcOmes in People with diabeteS (OCTOPuS) randomised controlled trial to evaluate an outpatient pre-cardiac surgery diabetes management intervention: a study protocol
Source: BMJ Open. 2021 Jun 9;11(6):e050919. doi: 10.1136/bmjopen-2021-050919 (PMC8191627; doi:10.1136/bmjopen-2021-050919)
Supplement: Reviewer comments [file bmjopen-2021-050919.reviewer_comments.pdf]

PEER REVIEW HISTORY

BMJ Open publishes all reviews undertaken for accepted manuscripts. Reviewers are asked to complete a checklist review form (**http://bmjopen.bmj.com/site/about/resources/checklist.pdf**) and are provided with free text boxes to elaborate on their assessment. These free text comments are reproduced below.

ARTICLE DETAILS

**TITLE (PROVISIONAL)**	The Optimising Cardiac Surgery ouTcOmes in People with diabeteS (OCTOPuS) randomised-controlled trial to evaluate an out-patient pre-cardiac surgery diabetes management intervention: a study protocol
**AUTHORS**	Holt, Richard; Dritsakis, Giorgos; Barnard-Kelly, Katharine; Thorne, Kerensa; Whitehead, Amy; Cohen, Lauren; Dixon, Elizabeth; Patel, Mayank; Newland-Jones, Philip; Green, Mark; Partridge, Helen; Luthra, Suvitesh; Ohri, Sunil; Salhiyyah, Kareem; Lord, Joanne; Niven, John; Cook, Andrew

This article was not externally reviewed at BMJ Open. Protocols that have been independently assessed prior to submission to BMJ Open are usually fast-tracked to publication on the grounds that further substantial changes will not be possible. This independent assessment will usually be external, independent review for both a substantial grant award from a non-commercial or government funder and ethics approval.
